# A reappraisal of the morphology and systematic position of the theropod dinosaur *Sigilmassasaurus* from the “middle” Cretaceous of Morocco

**DOI:** 10.7717/peerj.1323

**Published:** 2015-10-20

**Authors:** Serjoscha W. Evers, Oliver W.M. Rauhut, Angela C. Milner, Bradley McFeeters, Ronan Allain

**Affiliations:** 1 Department of Earth Sciences, University of Oxford, Oxford, United Kingdom; 2 Department für Geo- und Umweltwissenschaften, Ludwig-Maximilians-Universität München, Munich, Germany; 3 Bayerische Staatssammlung für Paläontologie und Geologie, Staatliche Naturwissenschaftliche Sammlungen Bayerns (SNSB), Munich, Germany; 4 Department für Geo- und Umweltwissenschaften and GeoBioCenter, Ludwig-Maximilians-University, Munich, Germany; 5 Department of Earth Sciences, Natural History Museum, London, United Kingdom; 6 Department of Earth Sciences, Carleton University, Ottawa, Canada; 7 Département Histoire de la Terre, Centre de Recherches sur la Paléobiodiversité et les Paléoenvironnements (CR2P), Muséum National d’Histoire Naturelle, Université Pierre et Marie Curie, Paris, France

**Keywords:** Africa, Spinosauridae, Kem Kem, Vertebral morphology

## Abstract

*Sigilmassasaurus brevicollis* is an enigmatic theropod dinosaur from the early Late Cretaceous (Cenomanian) of Morocco, originally based on a few isolated cervical vertebrae. Ever since its original description, both its taxonomic validity and systematic affinities were contentious. Originally considered to represent its own family, Sigilmassasauridae, the genus has variously been suggested to represent a carcharodontosaurid, an ornithischian, and, more recently, a spinosaurid. Here we describe new remains referrable to this taxon and re-evaluate its taxonomic status and systematic affinities. Based on the new remains, a re-evaluation of the original materials, and comparisons with other spinosaurids, the holotype of *Sigilmassasaurus brevicollis* is identified as an anterior dorsal, rather than a cervical vertebra, and differences between elements referred to this taxon can be explained by different positions of the elements in question within the vertebral column. Many characters used previously to diagnose the genus and species are found to be more widespread among basal tetanurans, and specifically spinosaurids. However, the taxon shows several autapomorphies that support its validity, including the presence of a strongly rugose, ventrally offset triangular platform that is confluent with a ventral keel anteriorly in the mid-cervical vertebral centra and a strongly reduced lateral neural arch lamination, with no or an incomplete distinction between anterior and posterior centrodiapophyseal laminae in the posterior cervical and anterior dorsal vertebrae. We argue furthermore that *Spinosaurus maroccanus*, also described on the basis of isolated cervical vertebrae from the same stratigraphic unit and in the same paper as *Sigilmassasaurus brevicollis*, is a subjective synonym of the latter. Both a detailed comparison of this taxon with other theropods and a formal phylogenetic analysis support spinosaurid affintities for *Sigilmassasaurus*. However, we reject the recently proposed synonymy of both *Spinosaurus maroccanus* and *Sigilmassasurus brevicollis* with *Spinosaurus aegyptiacus* from the Cenomanian of Egypt, as there are clear differences between the vertebrae of these taxa, and they do not share any derived character that is not found in other spinosaurids. Together with a comparison with other spinosaurid vertebral material from the Kem Kem, this suggests that more than one taxon of spinosaurid was present in the Kem Kem assemblage of Morocco, so the referral of non-overlapping material from this unit to a single taxon should be regarded with caution.

## Introduction

Despite several new discoveries in recent decades, the Cretaceous dinosaur fossil record of Africa is still rather poor. Cretaceous African theropods are mainly known from the ‘Middle’-Cretaceous (Aptian–Cenomanian) of northern Africa (Rauhut, 2008). The first theropods of that age came from the Baharyia oasis of Egypt, and were described in a series of papers by Stromer (1915; 1931; 1934), who erected the new taxa *Spinosaurus aegyptiacus* Stromer, 1915, *Carcharodontosaurus* Stromer, 1931 (to include the species “*Megalosaurus*” *saharicus* Depéret & Savornin (1927); Stromer, 1931) and *Bahariasaurus ingens* Stromer, 1934. Stromer (1934) also described additional theropod remains that could not be referred to any known or new taxon, amongst which several vertebrae and limb bones were considered to derive from an animal related to *Spinosaurus* and consequently informally denominated as “*Spinosaurus* B.” Since Stromer’s time especially, the roughly contemporaneous sediments of the Kem Kem area of Morocco have yielded Cenomanian theropod remains from Africa (Cavin et al., 2010). The first theropod remains from these layers were briefly mentioned, though neither described in detail nor figured, by Lavocat (1954), who noted similarities of the remains with *Carcharodontosaurus* and *Elaphrosaurus* Janensch, 1920. Later, Buffetaut (1989) described a fragmentary spinosaurid maxilla from the Kem Kem area that he referred to *Spinosaurus* cf. *aegyptiacus* (in spite of lacking overlap with the type material), but it was not until 1996, when more diagnostic material from these beds was reported. Russell (1996) described isolated dinosaur bones from this area, for which he created a new species of *Spinosaurus*, *Spinosaurus maroccanus* Russell, 1996, and a new genus and species of uncertain affinities, *Sigilmassasaurus brevicollis* Russell, 1996. Both taxa were based on isolated vertebrae. Russell furthermore described fragmentary material that he assigned to abelisaurids and carcharodontosaurids. In the same year, Sereno et al., 1996 described an almost complete skull they referred to *Carcharodontosaurus*, as well as another new taxon, *Deltadromeus agilis* Sereno et al., 1996. The latter was originally considered to be a basal coelurosaur, but it is currently generally regarded as a ceratosaur (Carrano & Sampson, 2008). Further finds suggested the presence of *Spinosaurus* (or a closely related taxon, as the material in question has no shared autapomorphic features with the holotype) and abelisaurids in the Kem Kem beds (e.g., Dal Sasso et al., 2005; Mahler, 2005), but the theropod fauna from this unit remains poorly known. Very fragmentary remains suggest the presence of two distinct carcharodontosaurids in the Kem Kem compound assemblage (Cau, Dalla Vecchia & Fabbri, 2012; Cau, Dalla Vecchia & Fabbri, 2013), and the presence of two taxa of spinosaurs has also been indicated recently (Richter, Mudroch & Buckley, 2013).

The taxonomic and systematic status of the theropod dinosaur *Sigilmassasaurus brevicollis* is currently under debate (see Sereno et al., 1996; Brusatte & Sereno, 2007; Evers, Rauhut & Milner, 2012; McFeeters et al., 2013; Ibrahim et al., 2014a; Allain, 2014). Russell (1996) erected the genus *Sigilmassasaurus*, within a new family Sigilmassasauridae on the basis of material acquired from an England-based fossil dealer, who had acquired the fossils from Moroccan locals in the Tafilalt region of Morocco. Russell (1996) recognized that the specimens resembled cervical vertebrae from the Cenomanian Bahariya Oasis of Egypt, described by Ernst Stromer as “*Spinosaurus* B” (Stromer, 1934) and concluded that the material belonged to the same taxon. He also found the material sufficiently different from *Spinosaurus* to justify the erection of the new family and genus. Russell thus created a new taxon, *Sigilmassasaurus brevicollis*, although he left some vertebrae in open nomenclature as *Sigilmassasaurus sp.*, noting that they might be older than the type and referred material of this species.

Russell (1996) also noted that the “*Spinosaurus* B” material was different from that referred to *Carcharodontosaurus saharicus* by Stromer (1934), thus precluding the possibility that *Sigilmassasaurus* material belonged to *Carcharodontosaurus.* Because the material described by Stromer (1934) was destroyed during World War II (Rauhut, 2005), Stromer’s plates and texts remain the only source for comparison of *Sigilmassasaurus* with “*Spinosaurus* B.”

The first opposing views were published shortly after the establishment of the genus *Sigilmassasaurus*. Sereno et al. (1996) proposed an ‘overlap’ of Stromer’s “*Spinosaurus* B” with *Carcharodontosaurus* material. In the same study, and also in a later paper (Brusatte & Sereno, 2007), the authors illustrate vertebral material very similar to *Sigilmassasaurus* vertebrae as belonging to different species of *Carcharodontosaurus*, although the association with *Carcharodontosaurus* skull material is questionable in each case. In 1998, Sereno and colleagues (1998) formally argued that *Sigilmassasaurus brevicollis* is a junior synonym of *Carcharodontosaurus saharicus*. Consequently, they treated the *Sigilmassasaurus* cervicals as *Carcharodontosaurus* material.

Some workers have pointed out that no *Carcharodontosaurus* cranial material has actually been found in articulation, or even in direct association with cervicals similar to those of *Sigilmassasaurus* (Canale, Novas & Haluza, 2008), and that *Sigilmassasaurus* vertebrae strongly differ from those of definitive carcharodontosaurids from South America (e.g., Novas et al., 2005; Canale, Novas & Haluza, 2008). Canale, Novas & Haluza (2008) noted that the vertebrae resemble those of *Iguanodon* and suggested a possible phylogentic position within Ornithischia. On the other hand Mahler (2005), in a passing comment, suggested that *Sigilmassasaurus* is the same as *Spinosaurus maroccanus*, and anatomical evidence for spinosaur affinities of the former taxon was presented by Evers & Rauhut (2012), Evers, Rauhut & Milner (2012), and Allain (2014). In their review of basal tetanuran theropods, Holtz, Molnar & Currie (2004) retained *Sigilmassasaurus* as a valid taxon and classified it as Tetanurae *incertae sedis*.

In a reevaluation of the holotype and referred material, McFeeters et al. (2013) assigned all the cervical vertebrae described by Russell (1996) plus some other material from the same beds and from other African localities to *Sigilmassasaurus brevicollis* (though some as *S*. cf. *brevicollis*). However, due to lack of diagnostic characters, McFeeters et al. (2013) removed the dorsal and caudal vertebrae originally referred by Russell to *Sigilmassasaurus* from this taxon. These authors identified *Sigilmassasaurus* as a valid theropod taxon based on several autapomorphic features. Possible affinities with Ornithopoda were precluded due to the presence of pneumatic features synapomorphic to Saurischia (McFeeters et al., 2013; *contra* Canale, Novas & Haluza, 2008). In a phylogenetic analysis based on a modified version of the matrix of Carrano, Benson & Sampson (2012), McFeeters et al. (2013) recovered *Sigilmassasaurus* in a polytomy with diverse megalosauroids, metriacanthosaurids and coelurosaurs at the base of Tetanurae, but outside the clade Allosauria.

Ibrahim et al. (2014a) recently described an allegedly associated partial skeleton of a spinosaurid from the Kem Kem beds and argued that both *Sigilmassasaurus brevicollis* and *Spinosaurus maroccanus* represent junior synonyms of *Spinosaurus aegyptiacus*, although no detailed justifications for these referrals were given. This conclusion has been challenged even more recently on the basis of the report of the complete cervical series of the spinosaurid *Ichthyovenator* Allain et al., 2012 from the Savannakhet Basin (Allain, 2014), based on a phylogenetic analysis that retained *Sigilmassasaurus* as belonging to Spinosauridae (Allain, 2014).

The main objective of this study is to provide a revised overview of the anatomy and systematics of *Sigilmassasaurus*, based on vertebral material directly comparable with the holotype. Previously unpublished *Sigilmassasaurus* material housed in the Bayerische Staatssammlung für Paläontologie und Geologie in Munich, Germany, and the Natural History Museum, London, United Kingdom, is described in detail. The material includes vertebrae from middle neck positions, and well-preserved specimens from posterior cervical and anterior dorsal positions. Other *Sigilmassasaurus* vertebrae have been recently described in detail (McFeeters et al., 2013) and are here compared with the new material. Finally, we describe and discuss material referred to *Spinosaurus maroccanus*, as far as it is relevant to give a comprehensive overview of *Sigilmassasaurus*.

## Geological and Palaeontological Context

In recent times, the Cretaceous Moroccan vertebrate assemblage has been informally referred to as the ‘Kem Kem compound assemblage’ (Cavin et al., 2010), since many Moroccan vertebrate fossils, including the holotype vertebra of *Sigilmassasaurus brevicollis*, have been found in the Kem Kem region of Morocco (Russell, 1996). However, the Cretaceous outcrops in Southeastern Morocco producing material referred to this assemblage extend beyond the Kem Kem area, and extend into the alluvial plain north of the Kem Kem area, which was called the ‘Tafilalt’ in Russell (1996). The outcrop area thus spans over parts of both of these regions of southeastern Morocco, and are geographically located in the eastern part of the Anti-Atlas area, south to the High Atlas and west to the Guir Hamada (Cavin et al., 2010).

Unfortunately, most material from this region lacks detailed locality information. This is in part because descriptions of localities of expeditions to remote areas before the late 20th century often lack precise locality data. Another, and even more important factor contributing to the lack of data, is the establishment of a market for vertebrate fossils coming from Morocco. Moroccan locals collect and excavate material without recording scientifically relevant data. These fossils are usually purchased and then resold by fossil dealers who operate on a global scale.

Yet another factor regarding the unsatisfactory geological context of Moroccan vertebrate remains is that the stratigraphy of the region has been subject to different approaches of systematization. In a recent paper, Cavin et al. (2010) tried to synthesize a stratigraphic concept for the area with information from earlier attempts and first hand field data. Several authors have recognized a succession of three sedimentologically distinct units in Cretaceous rocks of Southern Morocco (e.g., “trilogie mésocrétacée” of Choubert (1948); Sereno et al. (1996), who unite the bottom two units of Choubert’s systemization to the informal ‘Kem Kem beds’ but distinguish between a lower unit and an upper unit; and Dubar (1949), who formally erected three formations, which are (from bottom to top) the Ifezouane Formation, the Aoufous Formation, and the Akrabou Formation). It has been proposed that the Cretaceous of southern Morocco was deposited in two different, though maybe sporadically communicating basins (e.g., Choubert, 1948). Cavin et al. (2010), however, advocated that the deposition took place in a single sedimentary basin, and that the series displays a continuous time interval between the Early Cenomanian and the Middle Turonian. Accordingly, Cavin et al. (2010) use the formerly named formations by Dubar (1949) as a reference sequence for the entire southern Moroccan Cretaceous deposits.

Following this stratigraphic scheme, the informal ‘Kem Kem beds’ are equivalent to the bottom formations, i.e., the Ifezouane and Aoufous Formations. The term “Kem Kem compound assemblage” is useful to describe the vertebrate assemblage as such (Cavin et al., 2010), because until now only the Ifezouane and Aoufous Formations have produced such material, and in most of the cases material cannot be demonstrated to be derived from either formation with certainty.

The Ifezouane Formation lies unconformably on Paleozoic baserocks and consists of detritic, cross-stratified sandstones (Cavin et al., 2010). It decreases from south to north and has a maximum thickness of 250 m (Choubert, 1948). The Ifezouane Formation bears a lot of disarticulated vertebrate remains (Cavin et al., 2010). The Aoufous Formation is composed of marls, common gypsum layers, and inter-deposited detritic, clayey sandstones. It is 100–200 m in thickness and northern localities seem to be richer in fossils (Cavin et al., 2010). The Akrabou Formation lies conformably on the Aoufous Formation and comprises several marine transgressions (Ettachfini & Andreu, 2004; Ettachfini, 2008). The first of these can be dated to the lower part of the Upper Cenomanian on the basis of the occurrence of the ammonite *Neolobites vibrayeanus* (Cavin et al., 2010, and references within).

The Kem Kem compound assemblage has traditionally been considered “Infracénomanien” in age (i.e., at the base of the Cenomanian). Cavin et al. (2010) noted that there is indeed no evidence for pre-Cenomanian fossils at the base of the Cretaceous series. Also they pointed out that the Kem Kem compound assemblage is very similar to other North-African assemblages: The shark assemblage resembles that of the Bahariya Formation of Egypt (Sereno et al., 1996), which is dated safely to be Early Cenomanian (Catuneanu, Khalifa & Wanas, 2006), and the same is true for the dinosaur assemblage (though see below).

This shows that there is still work to be done to constrain the Cretaceous sediments of Morocco into a clear time frame, but it also suggests that the Kem Kem assemblage is Cenomanian, and most likely Early Cenomanian in age.

Besides various non-dinosaurian groups, the vertebrate remains from the Ifezouane and Aoufous Formations (the Kem Kem compound assemblage) include an array of dinosaurs, including abelisaurids, dromeosaurids, spinosaurs, carcharodontosaurids, ornithischian footprints (Sereno et al., 1996; Belvedere et al., 2013; Ibrahim et al., 2014b) and sauropods Lavocat, 1954; Cavin et al., 2010; Mannion & Barrett, 2013, and references therein Wilson & Allain, 2015). Only in few cases, vertebrate material has been unequivocally presented to be associated (Lavocat, 1954). The abundance of theropod material within the Kem Kem compound assemblage is remarkable (Läng et al., 2013). It has been suggested that the overabundance of theropod material in the ‘Kem Kem beds’ might actually be due to collector biases and commercial trade (McGowan & Dyke, 2009), or time averaging (Dyke, 2010). However, the unusually high percentage of theropod remains has also been found in more recent studies using systematic field approaches (Läng et al., 2013; Benyoucef et al., 2015) and holds true in other North African assemblages said to be of similar age as the ‘Kem Kem beds’ (Benyoucef et al., 2015). These studies suggest, that the high proportion of theropods is indeed indicative of an unbalanced ratio in the paleoenvironment (Läng et al., 2013). Läng et al. (2013) state that a preservational bias against (worn) teeth of herbivorous teeth is unlikely to alone explain the overabundance of theropod teeth, and that paleobiological explanations should be considered. Published biological and paleoecological explanations for this imbalance include attraction of predators to specific ecological settings such as streams (Russell, 1996), unusual food chains in (semi-) aquatic environments (Russell, 1996; Läng et al., 2013), or niche partitioning among predatory dinosaurs (Fanti et al., 2014).

## Material and Methods

### Material

We primarily describe new and previously undescribed specimens housed in the collections of the Bayerische Staatssammlung für Paläontologie und Geologie (BSPG) in Munich and the Natural History Museum (NHMUK) in London. Material referable to *Sigilmassasaurus* includes two mid-cervical vertebral centra (BSPG 2011 I 117 & 118), four posterior cervical (BSPG 2006 I 53 & 56, BSPG 2011 I 115 & 116) and two anterior dorsal (BSPG 2006 I 54 & 55) and, tentatively, one anterior mid-dorsal vertebra (BSPG 2013 I 95) and a mid-cervical vertebral neural arch (NHMUK PV R 16427) and three anterior dorsal vertebrae (NHMUK PV R 16434, 16435 & 16436). We frequently refer to the material, originally described by Russell (1996), which is housed in the Canadian Museum of Nature (CMN). All of this material was purchased by the respective institutions from fossil dealers, mainly from Moussa Minerals and Fossils, Cambridge. Thus, unfortunately, there is no detailed information on localities or association of individual specimens, but all certainly come from the Kem Kem beds in south–eastern Morocco (see Cavin et al., 2010).

Material housed in the BSPG was examined first hand by two of us (SWE, OWMR); material at the NHMUK (including *Baryonyx* Charig & Milner, 1986) was examined first hand by four of us (SWE, OWMR, ACM, RA); material housed at the University of Chicago (including the holotype and referred material of *Suchomimus* Sereno et al., 1998, vertebrae originally referred to *Carcharodontosaurus iguidensis* Brusatte & Sereno, 2007, and casts of the ‘neotype’ of *Spinosaurus aegyptiacus*) were examined first hand by two of us (SWE, OWMR); material from the Canadian Museum of Nature was examined first hand by two of us (BMF, ACM). Comparative material of *Ichthyovenator* has been collected in 2012 by one of us (RA) from the type locality of the taxon and will be described in detail in a forthcoming publication. Other comparative material was either examined first hand, or based on the published literature. Anatomical nomenclature follows Wilson (1999) for vertebral laminae and Wilson et al. (2011) for vertebral fossae.

### Computer tomography (CT) scanning

Two vertebrae were scanned with a Siemens medical computer tomography (CT) scanner at the Klinikum rechts der Isar in Munich. The scanning was done by Dr. Martin Dobritz. Scanning parameters were the following: 120 V Volatage, 175 mA X-ray tube current, 1,000 ms exposure time, 0.4 mm slice thickness. The data was examined using the freely available open source software OsiriX (Rosset, Spadola & Ratib, 2004) and 3D Slicer (http://www.slicer.org; Pieper, Halle & Kikinis, 2004; Pieper et al., 2006).

The DICOM data of the scans is deposited at figshare: BSPG 2006 I 54, 10.6084/m9.figshare.1471654; BSPG 2011 I 115, 10.6084/m9.figshare.1471659.

### Phylogenetic analysis

We used a modified version of the Carrano, Benson & Sampson (2012) data matrix (Data S2) to evaluate the phylogenetic relationships of *Sigilmassasaurus*. Cervical and dorsal characters were re-coded for all operational taxonomic units (OTUs), and several character definitions were modified. We also deleted a few of the original characters and added a number of new characters. *Sigilmassasaurus* was added to the list of OTUs, and material herein referred to *Sigilmassasaurus* was removed from the hypodigm of *Carcharodontosaurus*. Vertebral characters for *Carcharodontosaurus* are based on 1922 X 46 instead (Stromer, 1931). For *Baryonyx*, scorings are based on our new interpretation for the axial placement of preserved vertebral elements. Additionally, *Ichthyovenator* was added to the matrix, and the codings for *Spinosaurus aegyptiacus* were modified to be based on the holotype material only, and scored an additional OTU for the partial snout MSNM V4047, which was previously referred to *Spinosaurus* cf. *aegyptiacus* by Dal Sasso et al. (2005). For a full character description and a list of character codings for all taxa, see (Data S1).

The analysis was performed using TNT 1.1 (Goloboff, Farris & Nixon, 2008). *Eoraptor* Sereno et al., 1993 was chosen as the outgroup taxon. A heuristic search was carried out using 0 random seed for starting Wagner trees, and 10.000 replicates. The tree bisection reconnection (TBR) algorithm was applied, with 10 trees saved per replication. The collapse trees after search option was chosen. Minimum tree length was 1.041, and a strict consensus tree was calculated from 3.070 most parsimonious trees (see discussion).

## Systematic Paleontology

**Table:** 

Dinosauria Owen, 1842
Saurischia Seeley, 1887
Theropoda Marsh, 1881
Tetanurae Gauthier, 1986
Megalosauroidea Fitzinger, 1843
Spinosauridae Stromer, 1915
*Sigilmassasaurus* Russell, 1996
“*Spinosaurus* B”—Stromer, 1934: 8–18, 20–23, pl. 1, Tafel I, Figs. 2A–2C; partim
*Sigilmassasaurus brevicollis* Russell, 1996
*Spinosaurus maroccanus* Russell, 1996–Russell, 1996: 355–360, Figs 4 and 9; partim
*Sigilmassasaurus brevicollis* Russell, 1996–Russell, 1996: 361–360, Figs. 10, 11A, 13A, 13D and 13I; partim
*Sigilmassasaurus* sp. – Russell, 1996: 369–371, Figs. 14C, 14F, 14G and 15
*Sigilmassasaurus brevicollis* Russell, 1996–McFeeters et al., 2013: Figs. 1–7
*Spinosaurus aegyptiacus* Stromer, 1915–Ibrahim et al., 2014a, partim
*Holotype*: CMN 41857, a first dorsal vertebra.
*Referred material*: BSPG 2006 I 53, 54, 55, 56; BSPG 2011 I 115, 116, 117, 118; BSPG 2013 I 95; MNN IGU11 (see McFeeters et al., 2013; NHMUK PV R 16427, 16434, 16435; CMN 41774, 41790, 41850, 41856, 41857, 41858; P.P.No 481 see McFeeters et al., 2013); CMN 50791 (holotype of *Spinosaurus maroccanus*, see Russell, 1996); SGM–DIN 3, 5 (see McFeeters et al., 2013).
*Type locality and horizon*: Kem Kem area, probably close to the town of Taouz (K Martyn, pers. comm. to OR, 2013), Ifezouane or Aoufous Formation, Cenomanian (Cavin et al., 2010).

*Occurrences*: ‘Kem Kem beds,’ Ifezouane and/or Aoufous Formation, Cenomanian, south–eastern Morocco. We only refer material from these units to *Sigilmassasaurus maroccanus*, as there is insufficient data to exclude with certainty that similar material from other areas and geological untis might represent different species. However, very similar material, such as at least parts of the specimen described as “*Spinosaurus* B” by Stromer (1934) from the Cenomanian of Egypt or a vertebra referred to *Carcharodontosaurus* by Brusatte & Sereno (2007) from the Cenomanian of Niger can be referred to a spinosaurid close to *Sigilmassasaurus*.

**Figure 1:**
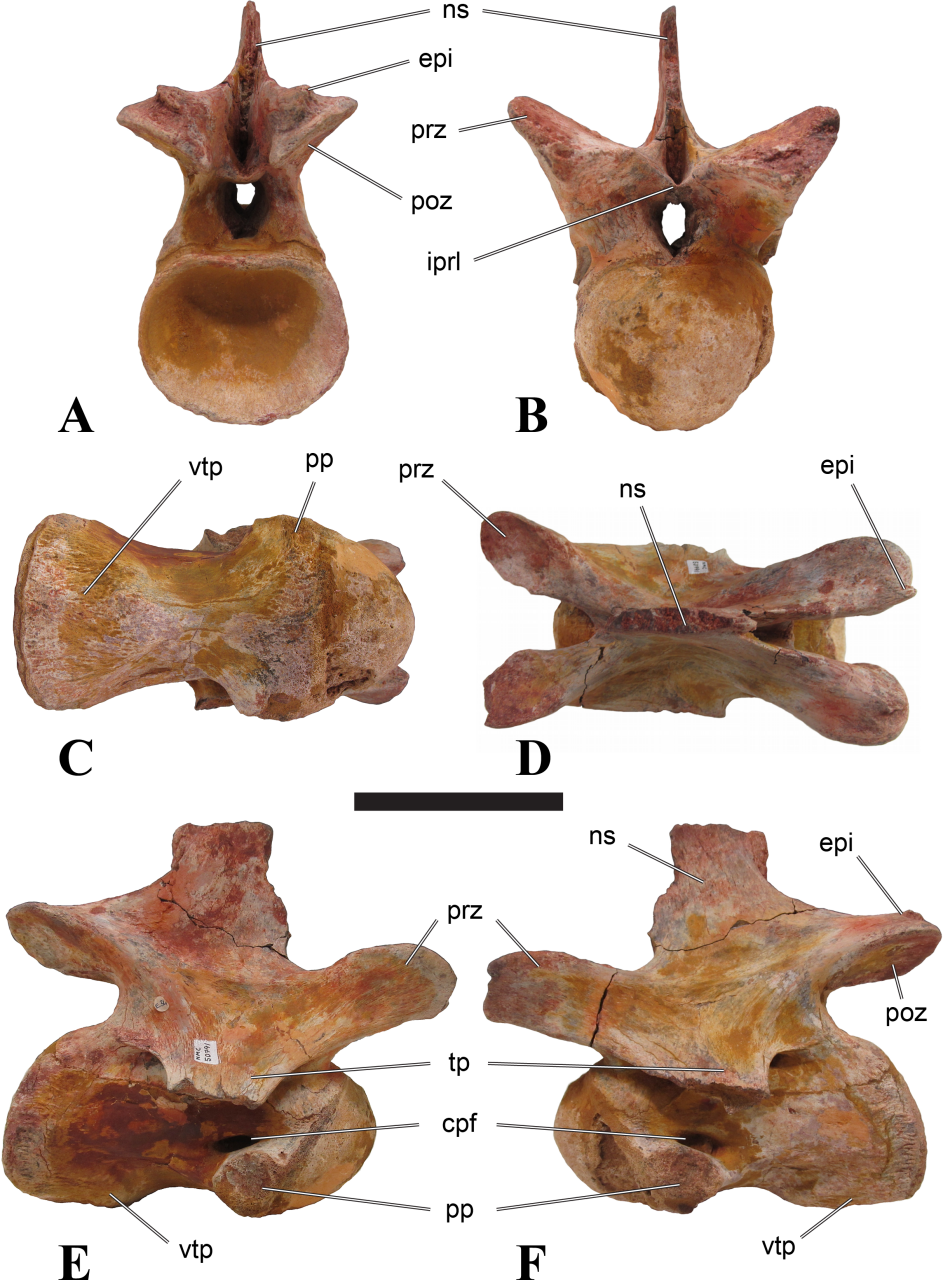
CMN 50791, mid-cervical vertebra (C6) of *Sigilmassasaurus brevicollis*. (A) posterior view; (B) anterior view; (C) ventral view; (D) dorsal view; (E) right lateral view; (F) left lateral view. Abbreviations: cpf, central pneumatic foramen; epi, epipophyses; iprl, interprezygapophyseal lamina; ns, neural spine; poz, postzygapophysis; pp, parapophysis; prz, prezygapophysis; tp, transverse process; vtp, ventral triangular plateau. Scale bar equals 10 cm.

**Figure 2:**
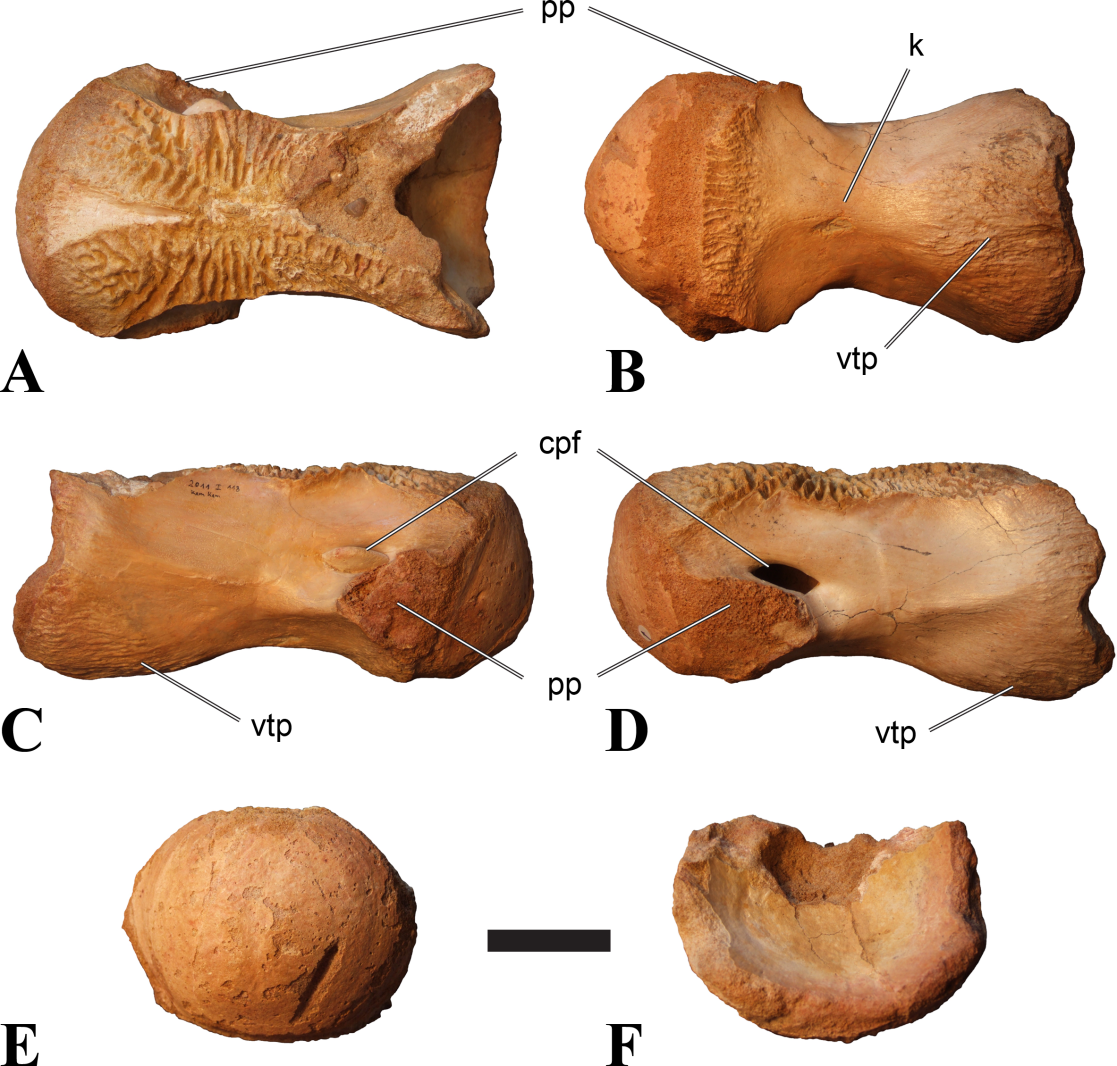
BSPG 2011 I 118, mid-cervical vertebra (C5) of *Sigilmassasaurus brevicollis*. (A) posterior view; (B) anterior view; (C) ventral view; (D) dorsal view; (E) right lateral view; (F) left lateral view. Abbreviations: cpf, central pneumatic foramen; k, keel; pp, parapophysis; vtp, ventral triangular plateau. Scale bar equals 5 cm.

**Figure 3:**
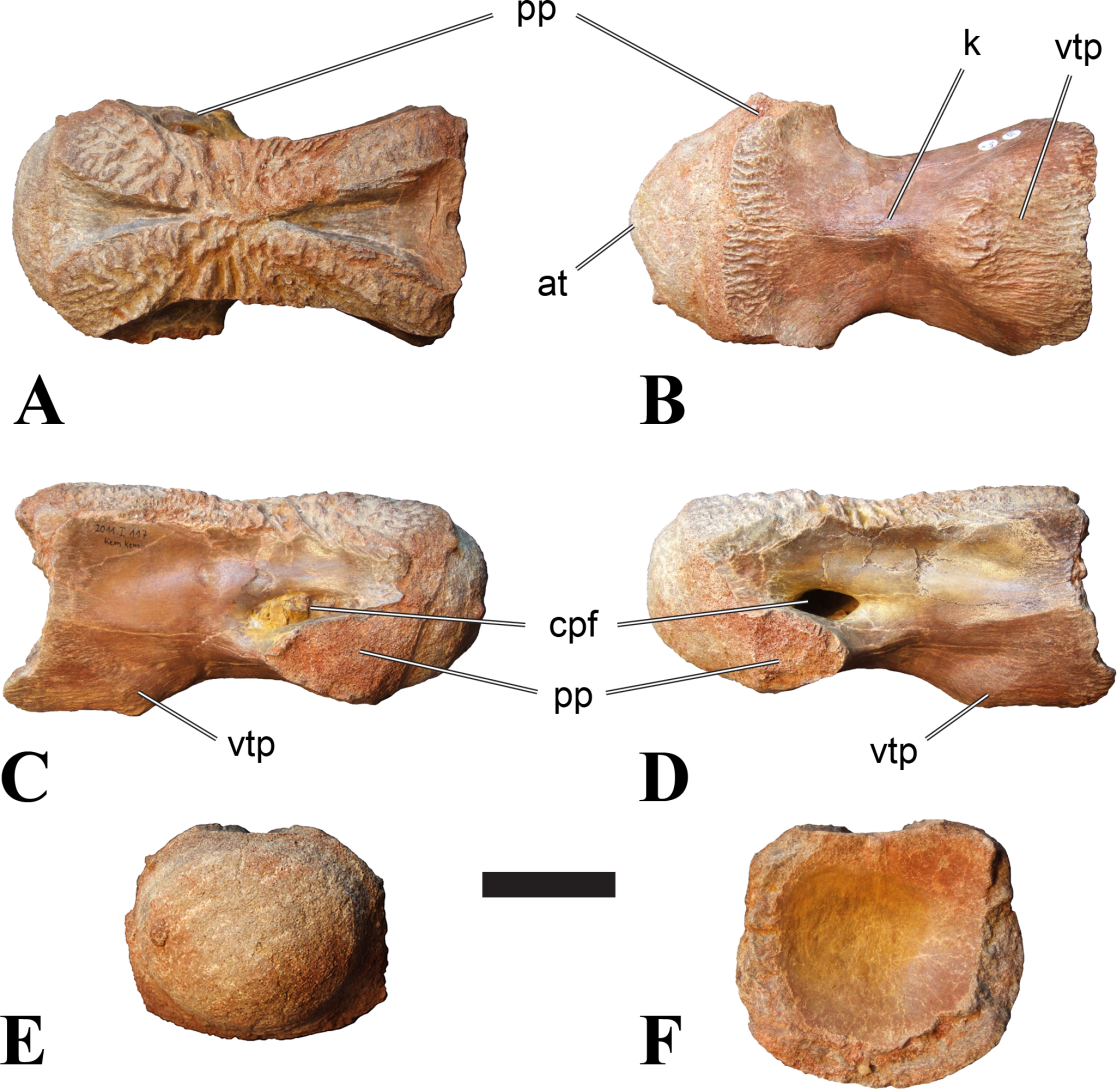
BSPG 2011 I 117, mid-cervical vertebra (C6) of *Sigilmassasaurus brevicollis*. (A) posterior view; (B) anterior view; (C) ventral view; (D) dorsal view; (E) right lateral view; (F) left lateral view. Abbreviations: at, anterior median tuberosity; cpf, central pneumatic foramen; k, keel; pp, parapophysis; vtp, ventral triangular plateau. Scale bar equals 5 cm.

**Figure 4:**
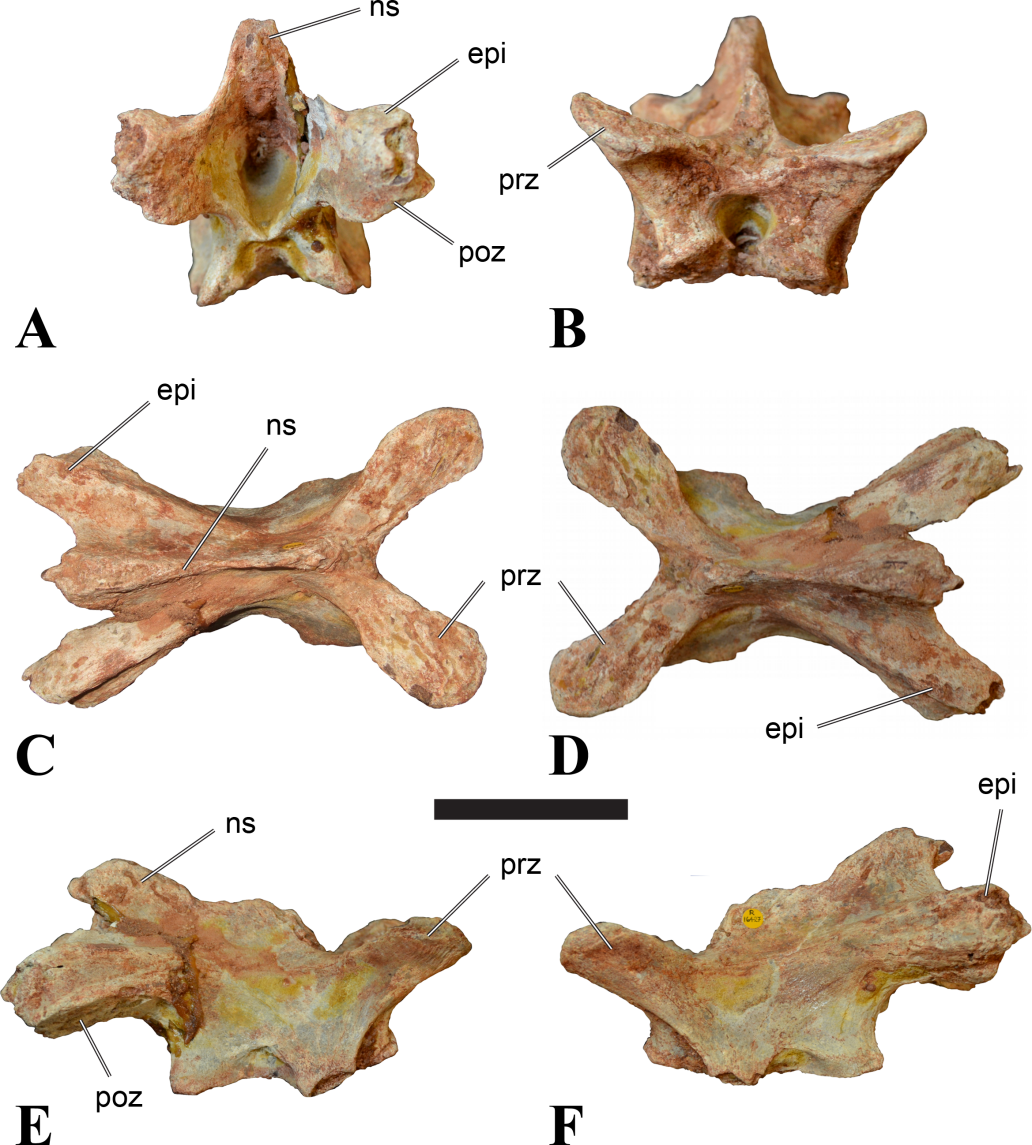
NHMUK PV R 16427, mid-cervical neural arch (C4) of *Sigilmassasaurus brevicollis*. (A) posterior view; (B) anterior view; (C) ventral view; (D) dorsal view; (E) right lateral view; (F) left lateral view. Abbreviations: epi, epipophyses; ns, neural spine; poz, postzygapophysis; prz, prezygapophysis. Scale bar equals 5 cm.

**Figure 5:**
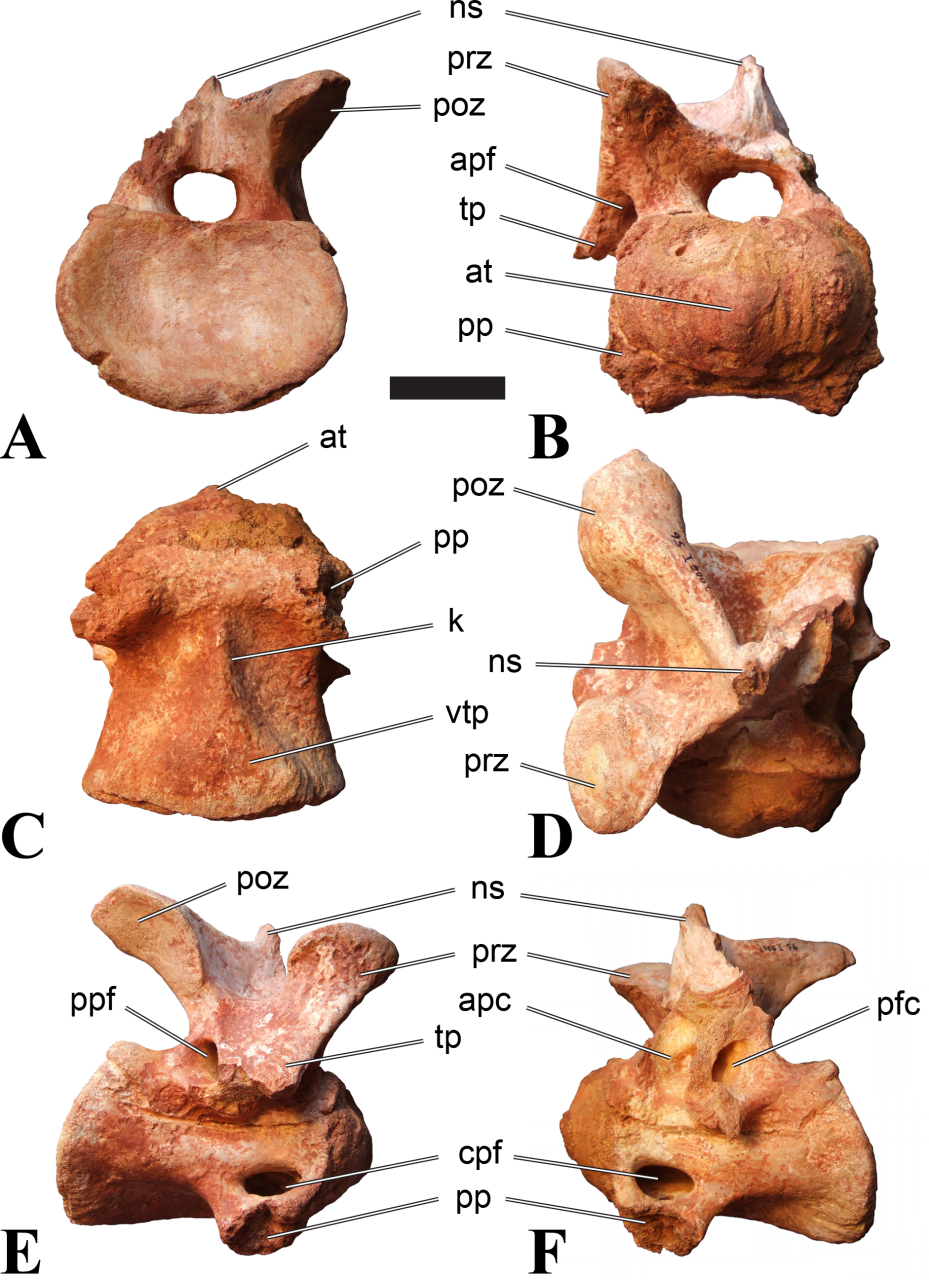
BSPG 2006 I 56, posterior cervical vertebra (C8) of *Sigilmassasaurus brevicollis*. (A) posterior view; (B) anterior view; (C) ventral view; (D) dorsal view; (E) right lateral view; (F) left lateral view. Abbreviations: apc, anterior pneumatic chamber of the transverse process; apf, anterior pneumatic foramen of the prezygodiapophyseal fossa; at, anterior medial tuberosity; cpf, central pneumatic foramen; k, keel; ns, neural spine; poz, postzygapophysis; pp, parapophysis; ppc, posterior pneumatic chamber of the transverse process; ppf, posterior pneumatic foramen of the postzygodiapophyseal fossa; prz, prezygapophysis; tp, transverse process; vtp, ventral triangular plateau. Scale bar equals 5 cm.

**Figure 6:**
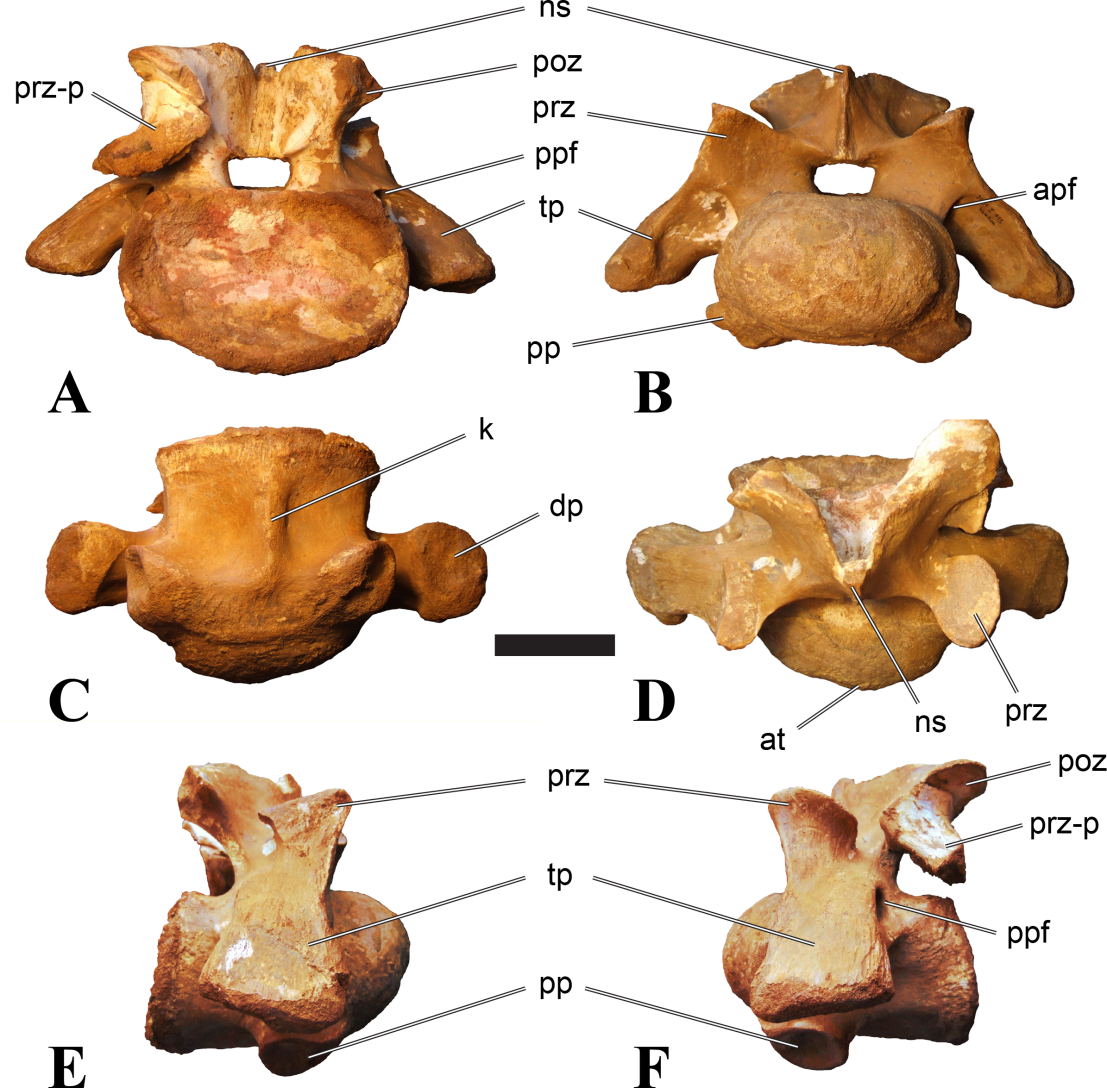
BSPG 2011 I 115, posterior cervical vertebra (C9) of *Sigilmassasaurus brevicollis*. (A) posterior view; (B) anterior view; (C) ventral view; (D) dorsal view; (E) right lateral view; (F) left lateral view. Abbreviations: apf, anterior pneumatic foramen of the prezygodiapophyseal fossa; at, anterior medial tuberosity; dp, diapophysis; k, keel; ns, neural spine; poz, postzygapophysis; pp, parapophysis; ppf, posterior pneumatic foramen of the postzygodiapophyseal fossa; prz, prezygapophysis; prz-p, prezygapophysis of posteriorly adjacent vertebra; tp, transverse process. Scale bar equals 5 cm.

**Figure 7:**
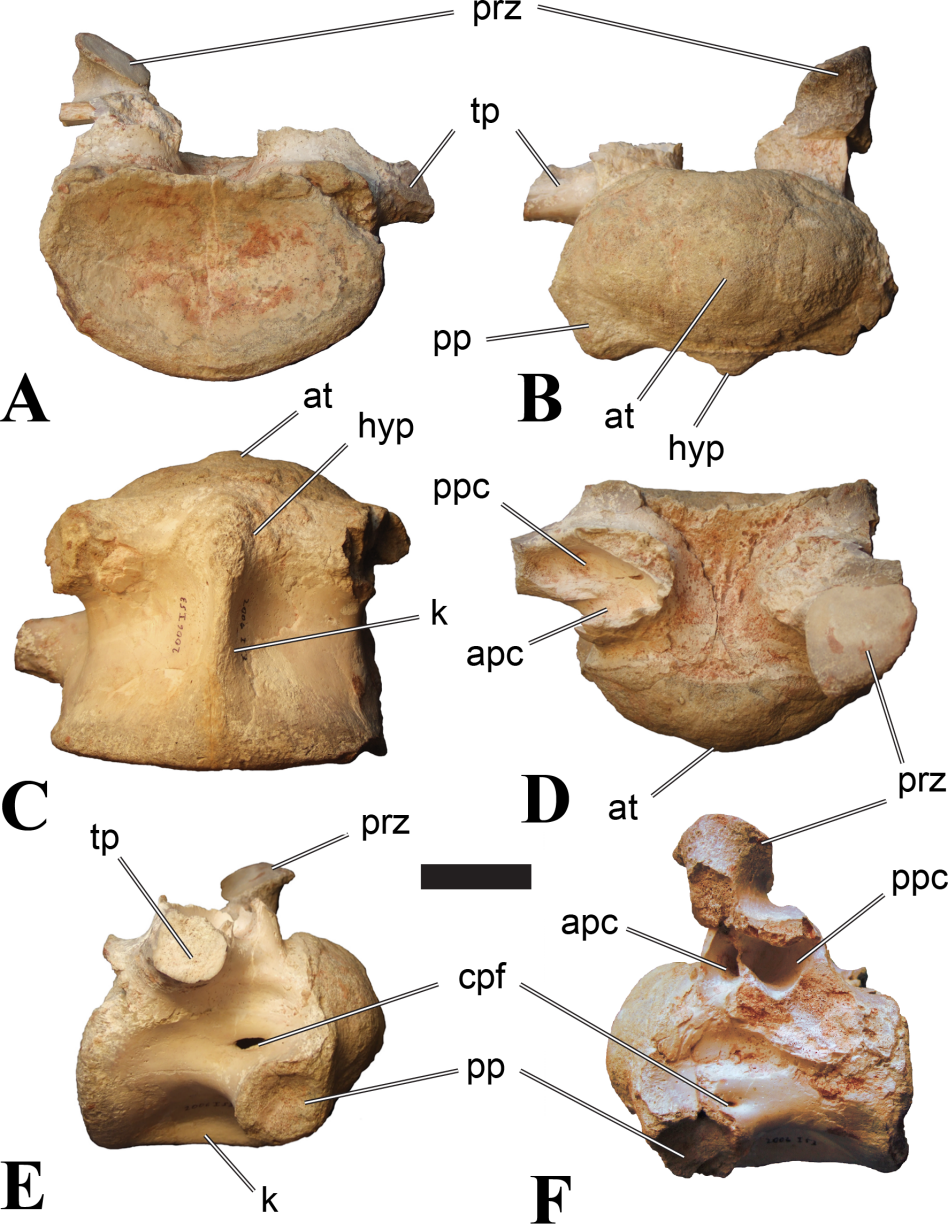
BSPG 2006 I 53, posterior cervical vertebra (C10) of *Sigilmassasaurus brevicollis*. (A) posterior view; (B) anterior view; (C) ventral view; (D) dorsal view; (E) right lateral view; (F) left lateral view. Abbreviations: apc, anterior pneumatic chamber of the transverse process; at, anterior medial tuberosity; cpf, central pneumatic foramen; hyp, hypapophysis; k, keel; pp, parapophysis; ppc, posterior pneumatic chamber of the transverse process; prz, prezygapophysis; tp, transverse process. Scale bar equals 5 cm.

*Emended diagnosis*: Very large spinosaurid theropod dinosaur. The taxon can be diagnosed on the basis of the following autapomorphies: mid-cervical vertebrae with offset, transversely convex, strongly rugose triangular platform at the posterior end of the ventral side that is confluent with a ventral keel anteriorly; anteriorly broad centroprezygapophyseal lamina with no or strongly reduced centroprezygapophyseal fossa already in anterior mid-cervical vertebrae; reduced neural arch lamination with no or incomplete distinction between anterior and posterior centrodiapophyseal laminae in posterior cervicals and first dorsal; small elongate fossa on either side of the base of the neural spine in last cervical and first dorsal vertebrae. Furthermore, *Sigilmassasaurus brevicollis* differs from most other theropods in the combination of the following characters: anterior articular surface of posterior cervical and anterior dorsal vertebrae more than 1.5 times wider than high and wider than length of centrum (also in *Ichthyovenator*); well-developed anterior tubercle present on the anterior articular surface in posterior cervical and anterior dorsal vertebrae; interzygapophyseal laminae absent in posterior cervicals and anteriormost dorsals, resulting in ventrally open spinopre- and spinopostzygapophyseal fossae (also in *Ichthyovenator*); posterior cervical and anterior dorsal vertebrae with massive transverse processes with deeply penetrating pneumatic openings at the base anteriorly and posteriorly (also in D1 in *Ichthyovenator*); epipophyses weakly developed in mid-cervicals and absent in posterior cervicals; posterior cervical and anteriormost dorsal vertebrae with anteroposteriorly short, posteriorly inclined, low and spike-like neural spines (modified from Russell, 1996; McFeeters, 2011; Evers, 2012; McFeeters et al., 2013).

*Comments*: The elongate shape of the holotype vertebra of *Spinosaurus maroccanus* is very unlike the ‘typical’ *Sigilmassasaurus* morphology of a cervical or anterior dorsal vertebra with extraordinarily broad intercentral articulations and relatively short centrum. However, we present evidence that there is a continuum between the morphology of the holotypes of *Sp. maroccanus* and *S. brevicollis* (see discussion below) as seen in *Ichthyovenator* (Allain, 2014; R Allain, 2015, unpublished data). General trends in the axial sequence of theropod dinosaurs show a reduction in length–width relations, among other features, which explain the observed differences between *Sp. maroccanus* and *Sigilmassasaurus* morphotypes. Furthermore, the vertebrae share a number of characters that are unique or otherwise unusual in theropod dinosaurs, supporting the synonymy of the two taxa. However, we do not accept the recent synonymization of both of these names with *Spinosaurus aegyptiacus* (Ibrahim et al., 2014a; see discussion below).

Both *Spinosaurus maroccanus* and *Sigilmassasaurus brevicollis* were described in the same paper (Russell, 1996). Although the former was mentioned first in the respective paper, both names are available, as the ICZN does not formally recognize page priority. As our analysis indicates that the species comprising *Sigilmassasaurus brevicollis* and *Spinosaurus maroccanus* is not referable to the genus *Spinosaurus*, we used the other available generic name, which is *Sigilmassasaurus*. Without page priority, the species epithet *maroccanus* has no priority over the species epithet *brevicollis*, and in spite of the latter being unfortunate because descriptively inadequate according to our neck reconstruction, we decided to keep the name *Sigilmassasaurus brevicollis* for taxonomic simplicity.

## Description

### Axial positioning of the vertebrae

Establishing the position of the isolated vertebrae described here within the axial skeleton of *Sigilmassasaurus* proved difficult. This is partly due to the lack of descriptions of complete presacral vertebral columns in other known spinosaurids, and partially owing to the fact that the material at hand represents different individuals of different ontogenetic stages, making it difficult to evaluate the influence of individual and ontogenetic variation. Furthermore, the preservation of the elements is variable. Nevertheless, the relative axial positions of *Sigilmassasaurus* specimens were established on the basis of general trends in changes of vertebral morphology observed in tetanurans and in comparison with a complete, but so far undescribed cervical vertebral column of the spinosaur *Ichthyovenator* (Allain, 2014). Specific morphological features change along the axial series as functional consequences of their position, and can therefore be observed in most taxa.

In most saurischian dinosaurs, the border between the cervical and dorsal vertebral series is marked by a notable dorsal shift of the parapophyses from the anteroventral end of the centrum onto the mid-height of the centrum or even the neurocentral suture, as for example in *Acrocanthosaurus* Stovall & Langston, 1950 (Harris, 1998) and *Sinraptor* Currie & Zhao (1993). Madsen (1976) figured the last vertebra with an anteroventrally placed parapophysis as the first dorsal vertebra in *Allosaurus* Marsh 1877 (see also Schachner et al., 2011), but he argued that there are only nine cervicals, which is in contrast to other basal tetanurans, in which ten cervicals are present, as indicated by specimens with articulated or directly associated ribs (e.g., Currie & Zhao, 1993; Rauhut et al., 2012). In such specimens, the change in rib morphology from cervical to dorsal ribs coincides with a dorsal shift of the parapophysis. Interestingly, *Ichthyovenator* represents an exception to this rule, as the parapophysis in the first dorsal vertebra is dorsoventrally extended, with its ventral extent still placed on the anteroventral edge of the centrum (BK 10-25), and the great similarity of the first dorsal vertebra with the type vertebra of *Sigilmassasaurus brevicollis* indicates that this is also the case in *Sigilmassasaurus*.

In most theropods, the transverse processes project ventrolaterally in anterior cervicals and become more elevated posteriorly until they reach a horizontal or even dorsolateral orientation in anterior dorsals (e.g., *Allosaurus:* Madsen, 1976; *Neovenator* Hutt et al., 1996: Brusatte, Benson & Hutt, 2008; *Acrocanthosaurus:* Stovall & Langston, 1950, *Monolophosaurus* Zhao & Currie, 1993: Zhao & Currie, 1993; Zhao et al., 2010, or *Majungasaurus* Lavocat, 1955: O’Connor, 2007). Russell (1996) used the transverse process elevation as the main indicator for his axial positioning of the holotype and referred material of *Sigilmassasaurus*, and we agree on the usefulness of this character.

The development of ventral keels is highly variable in theropods. In many basal theropods, ventral keels are weakly developed or even completely absent in mid-cervicals, but become more prominent towards the cervico–dorsal transition (e.g., *Baryonyx*: Charig & Milner, 1997; *Sinraptor*: Currie & Zhao, 1993). Most carcharodontosaurs, like *Acrocanthosaurus* or *Mapusaurus* Coria & Currie, 2006, also have strongly keeled posterior cervicals and anterior dorsals (Stovall & Langston, 1950; Coria & Currie, 2006). Hypapophyses, if present, are variable in theropods. They are most often found on one or two anterior-most dorsals (e.g., *Allosaurus:* Madsen, 1976; *Sinraptor:* Currie & Zhao, 1993), but are present in the last cervical vertebrae of *Piatnitzkysaurus* Bonaparte, 1986 (Bonaparte, 1986) and *Condorraptor* Rauhut, 2005 (Bonaparte, 1986). In this study, we accordingly interpret the ventral keel and hypapophysis of *Sigilmassasaurus* to develop progressively in an anteroposterior fashion in the cervical series, until they level off in dorsal vertebrae.

To achieve a sigmoidal curvature of the neck, as it is typical for large theropods, such as *Sinraptor* (Currie & Zhao, 1993), the articular facets of vertebrae have to be of specific orientation relative to the long axis of their respective centrum (Sereno, 1991). An anteroventral inclination in anterior cervicals is reversed in more posterior cervicals towards the base of the neck, to form the typical S-shape of the neck, as in *Monolophosaurus* (Zhao & Currie, 1993; Zhao et al., 2010) or *Sinraptor* (Currie & Zhao, 1993). Thus, the relative inclination of the articular surfaces can also be used to establish the region of the neck represented by single elements.

The width–height ratio of articular facets differs greatly in *Sigilmassasaurus* specimens. In some taxa with intercentral articulations wider than high, the centra become relatively wider posteriorly in the vertebral sequence. This is exemplified by *Baryonyx*, and to some extent present in *Eustreptospondylus* Walker, 1964 and *Cryolophosaurus* Hammer & Hickerson, 1994 (Charig & Milner, 1997; Sadleir, Barrett & Powell, 2008; Smith et al., 2007). In accordance with this development, higher width–height ratios in *Sigilmassasaurus* are interpreted as a character indicative of a more posterior position within the cervical region. In the dorsal vertebrae, a reversal of this trend is evident, with more posterior dorsals tending to show round articular facets.

In synthesis, several morphological changes can be used to identify (at least relative) vertebral positions in theropod dinosaurs. In *Sigilmassasaurus*, the anterior-to-posterior trends include a progressive change in orientation of the anterior articular facet from a vertical inclination to a dorsal inclination, an intensified development of the ventral keel and hypapophysis, and an ascending value of width–height ratio, and a progressively achieved dorsal elevation of transverse processes. Inverse trends along the cervical series include the elongation of the shape of the central pneumatic foramina, the overall centrum-length, and the inclination of the neural spine. These trends were applied to the *Sigilmassasaurus* material described within this study. For *Sigilmassasaurus* specimens examined, the distinct morphological trends explained above consistently allow interpretations on the axial placement. Comparisons with the vertebral column of *Ichthyovenator* allow allocation of the exact position of many, but not all of the isolated vertebrae. In the following description, we thus distinguish between mid-cervical, posterior cervical, and anterior dorsal vertebrae.

Thus, according to these criteria, we identified vertebrae BSPG 2011 I 117, BSPG 2011 I 118, CMN 50791 (holotype of *Sp. maroccanus*), and NHMUK PV R 16427 as mid-cervical vertebrae; BSPG 2006 I 53, BSPG 2006 I 56, BSPG 2011 I 115, BSPG 2011 I 116, CMN 41774, CMN 41790, and CMN 41856 as posterior cervical vertebrae; and BSPG 2006 I 54, BSPG 2006 I 55, BSPG 2013 I 95, CMN 41850, CMN 41857 (holotype of *S. brevicollis*), CMN 41858, NHMUK PV R 16434, NHMUK PV R 16435, and NHMUK PV R 16436 as anteriormost dorsal vertebrae. Measurements of the vertebrae described below are shown in Table 1.

**Table 1:** Measurements of vertebral specimens from the Kem Kem compound assemblage in the collections of the BSPG and NHMUK.

Specimen	Identification	Centrum length (mm)	Centrum length without anterior ball (mm)	Anterior centrum width (mm)	Anterior centrum height (mm)	Posterior centrum width (mm)	Posterior centrum height (mm)
BSPG 2011 I 118	*Sigilmassasaurus*, C5(?)	212	176^a^, 164^b^	106	88	119	100^c^
BSPG 2011 I 117	*Sigilmassasaurus*, C6	184	161^a^, 150^b^	90	76	108^c^	89
BSPG 2006 I 56	*Sigilmassasaurus*, C8	119	91^a^, 95^b^	86	61	110	74
BSPG 2011 I 115	*Sigilmassasaurus*, C9	123	76^a^, 94^b^	123	71	136	87
BSPG 2006 I 53	*Sigilmassasaurus,* C10	123	82^a^, 101^b^	128	73	146	88
BSPG 2011 I 116	*Sigilmassasaurus*, C10	123	79^a^, 95^b^	134	80	150	94
BSPG 2006 I 54	*Sigilmassasaurus*, D1	139	95^a^, 102^b^	143	82	155	92
BSPG 2006 I 55	*Sigilmassasaurus*, D1	81	58^a^, 68^b^	85	44	86	53
NHMUK PV R 16343	*Sigilmassasaurus,* D1	113	82^a^, 75^b^	146	76	135^c^	73
NHMUK PV R 16436	*Sigilmassasaurus,* D2	132	93^a^, 103^b^	124	70	126^c^	96
NHMUK PV R 16435	*Sigilmassasaurus,* D3	150	118^a^, 120^b^	126	81	NA	80^c^
BSPG 2013 I 95	?*Sigilmassasaurus*, ant. dorsal	168	135	110	93	120^c^	110
BSPG 2006 I 57	Spinosauridae indet., C6	198	159^a^, 140^b^	91	85	98^c^	100
BSPG 2013 I 97	Spinosauridae indet., C6 or 7	140	125^a^, 105^b^	64	51	69	63
NHMUK PV R 36637	Spinosauridae indet., ?C8	120	95^a^, 101^b^	115^c^	60^c^	115	76

**Notes.**

a length along the dorsal side.

b length along the ventral side.

c estimated element damaged.

Annotations NA not available due to degree of damage

Measurements of the anterior articular surface exclude parapophyses and hypapophysis.

### Mid-cervical vertebrae

Vertebrae identified as mid-cervicals are BSPG 2011 I 117, BSPG 2011 I 118, CMN 50791 (type of *S. maroccanus*; Fig. 1), and NHMUK PV R 16427. BSPG 2011 I 117 and BSPG 2011 I 118 are centra isolated from their neural arches. In both vertebrae, the ornamentation of the areas for neural arch articulations is largely intact, suggesting the neural arches and centra had not yet fused at time of death. Accordingly, the animals probably died before somatic adulthood. This is quite remarkable, because BSPG 2001 I 118 is a particularly large specimen reaching 160 mm in length, excluding the anterior condyle. NHMUK PV R 16427 is an isolated neural arch. The isolated centra, as well as the isolated neural arch all share important features with CMN 50791.

Centra of mid-cervical vertebrae of *Sigilmassasaurus* are longer than wide, and only slightly wider than high. The anterior articular condyles face slightly anteroventrally in respect to the long axis of the vertebra. Transverse processes are strongly ventrally inclined, epipophyses and ventral keels on the centrum are weakly developed but present, while hypapophyses are completely absent.

Both centra BSPG 2011 I 117 and 118 (Figs. 2 and 3), as well as CMN 50791 display a different centrum morphology than the ‘typical’ vertebrae referred to *Sigilmassasaurus brevicollis* (e.g., holotype CMN 41857). The centra are elongate, and the length (without anterior condyle) corresponds to 167% of the width of the anterior facet in BSPG 2011 I 117, and equals 162% of the width in BSPG 2011 I 118. Also the width–height ratios are low with 1.15 (anterior facet) and 1.21 (posterior facet) in BSPG 2011 I 117, and 1.33 (anterior facet) and 1.26 (posterior facet) in BSPG 2011 I 118. These measurements compare well to those of the holotype of *S. maroccanus* (CMN 50791), in which the length corresponds to c. 175% of the anterior width and the width–height ratio of the anterior face is approximately 1.16.

BSPG 2011 I 117 and 118, and CMN 50791 are strongly opisthocoelous and share anterior articular facets that are ventrally inclined at about an angle of 17–20° from the perpendicular to the long axis of the centra, and are slightly displaced dorsally from the level of the posterior surface. This displacement is more marked in BSPG 2011 I 118 than in the other two elements. The posterior facets lack the distinct, notably reniform outlines of the holotype of *Sigilmassasaurus brevicollis*, but the dorsal edge of the posterior articular facet is slightly concave, contributing to a weakly reniform outline. In BSPG 2011 I 118 the dorsal part of the posterior articular facet is eroded so that the shape cannot be reconstructed with certainty. Both isolated centra display a low median tuberosity on the condyle of the anterior side of the vertebrae, which is better developed in BSPG 2011 I 117 than in BSPG 2011 I 118. In the latter specimen, the median tuberosity has a vertically oriented depression, which is not evident in other specimens. No median tuberosity could be confirmed for CMN 50791.

An elongate but narrow groove is incised into the surface on the left side of the anterior condyle of BSPG 2011 I 118. It is 3–4 mm deep and 40 mm long. The surface of the groove is rough, and the edges seem to have experienced some erosion. A sandgrain is tightly embedded in one edge of the mark, indicating that this structure was present prior to fossilisation. This groove differs from the definite chisel marks seen in another specimen (BSPG 2006 I 56, see below), which show quadratic outlines, smooth surfaces, and sharp, uneroded edges. An alternative interpretation supported here is that the feature represents a post-mortem bite mark.

Ventral keels are present in the anterior half of the ventral sides of BSPG 2011 I 117 and 118, but developed only as a shallow ridge. A prominent feature of the ventral side is an elevated triangular platform of bone in its posterior half, which anteriorly merges into the keel, and posteriorly connects to the rim of the posterior articular facet. The ventral keel is better developed in BSPG 2011 I 117, where it continues anteriorly from the platform as a broad, rounded ridge to a pronounced rugose area at the anterior end of the centrum, whereas it is only marked as a slightly raised, broad ventral area in BSPG 2011 I 118, where it fades anteriorly just posterior to a similar rugose patch. Both the triangular ventral platform and a broad, weakly developed ventral keel are also evident in CMN 50791. This condition is very different from that seen in posterior cervicals or cervicodorsal vertebrae of *Sigilmassasaurus*, but an intermediate condition is seen in BSPG 2006 I 56, which also bears a prominent posteroventral triangular plateau, but has a more pronounced keel (see below for detailed description). The triangular elevated plateau is an important feature indicating that the presented specimens are congeneric, since such a well-developed and strongly offset plateau that merges with a low keel anteriorly is unknown in other theropods, and can thus be regarded as an autapomorphy of *Sigilmassasaurus*. The only other theropod with a similar feature is *Ichthyovenator*, in which a ventral platform, which is, however, not continuous anteriorly with a keel, is present in the Ce7 and Ce8 (BK10 21; BK10-22). Usually, the ventral surface of mid-cervical theropod vertebrae is either concave, as in *Sinraptor* (Currie & Zhao, 1993), or a narrow keel runs along the entire ventral side of the centrum, as in keeled vertebrae of *Neovenator* (Brusatte, Benson & Hutt, 2008). Importantly, the triangular plateau is neither found in *Spinosaurus aegyptiacus* (Stromer, 1915), *Baryonyx* (Charig & Milner, 1997), *Suchomimus* (S Evers & O Rauhut, pers. obs., 2015), nor in carcharodontosaur vertebrae, e.g., *Giganotosaurus* (MUCPv–CH–1), *Tyrannotitan* (Novas et al., 2005) (MPEV–PV 1157), or *Acrocanthosaurus* (Harris, 1998).

A hypapophysis is absent in BSPG 2011 I 117, BSPG 2011 I 118, and CMN 50791. Anteriorly, the keel fades towards a slightly elevated transverse connection of the parapophyses that occupies the anterior margin of the centrum. Both this transverse area and the triangular plateaus in BSPG 2011 I 117 and 118, as well as in CMN 50791, bear an intensely rugose surface structure. The rugosity is composed of numerous small ridges and furrows, which align in a longitudinal pattern. Although this texture is developed most prominently in BSPG 2011 I 117 and 118, the rugosities can also be observed in specimens BSPG 2006 I 55, BSPG 2011 I 115, and BSPG 2011 I 116, NHMUK PV R 16435 (other specimens either show only weak rugosities, or respective areas on the centrum are too damaged to determine the character). The rugosities are furthermore evident in the holotype of *Sigilmassasaurus brevicollis* (see Russell, 1996; McFeeters et al., 2013), although the structures have not been mentioned in previous works. A vertebra referred to as *Carcharodontosaurus iguidensis*, which we consider to represent *Sigilmassasaurus* or a closely related form, also shows the same pattern (Brusatte & Sereno, 2007). These rugosities are more strongly developed than in other theropod dinosaurs, and are thought to be related to hypaxial muscle attachment (Snively & Russell, 2007).

The parapophyses of BSPG 2011 I 117 and CMN 50791 seem to be mainly laterally directed, while the orientation has a more ventral component in BSPG 2011 I 118. The latter is hard to constrain, however, because the parapophyses are heavily eroded in this vertebra. In all vertebrae, the parapophyses are placed at the anterior rim and positioned ventrally on the centrum. Because of the ventral tilt of the anterior facet, the parapophyses appear to be placed somewhat more posteriorly on the centrum, but their position is actually consistent with that of the parapophyses observed in other cervical and the anteriormost dorsal vertebrae of *Sigilmassasaurus*. The articular facets of the parapophyses are large and semioval in outline, tapering posteriorly. Posteriorly, the parapophyses are connected to the lateral side of the centrum by a short but stout ridge. This ridge is best developed in BSPG 2011 I 117, but is shared among all three vertebrae. In continuation to this posterior parapophyseal ridge a notable edge marks the sharp transition from lateral surface and ventral surface of the centrum. This edge extends from the ridge posteriorly and slightly dorsally, so that it meets the posterior end at about the mid-height of the centrum. About halfway between the parapophysis and the posterior end of the centrum there is a low, rounded tubercle on this edge; this tubercle is also present in all three vertebrae.

The lateral pneumatic foramina are located posterodorsal to the parapophyses. They are symmetrically developed and are anteroposteriorly elongate. In BSPG 2011 I 117, the better preserved foramen on the left side is 25 mm long and maximally 10 mm high; these measurements are 28 mm and 10 mm, respectively, in BSPG 2011 I 118. In BSPG 2011 I 117 and 118, both the anterior and the posterior rim of the foramen taper to a sharply angled point; in CMN 50791 this is only the case in the anterior margin, whereas the posterior margin is narrow, but rounded. A very shallow, triangular depression is present posterior to these pneumatic foramina. Anteriorly, the pneumatic foramen also incises into the dorsal surface of the base of the parapophysis. In both BSPG 2011 I 117 and 118, the pneumatic foramina lead into large internal cavities, with a smaller, anteroposteriorly elongate ventral depression just adjacent to the foramen being separated from a larger medial cavity by a low, rounded ridge. The surface directly above the pneumatic foramina is slightly swollen. This feature was also observed by McFeeters et al. (2013) in the holotype material of *Sigilmassasaurus*, and has been described as a lateral bulge. Between this bulge and the neurocentral suture the lateral surface is very gently concave dorsoventrally.

In dorsal view, the centra are medially constricted. The narrowest part of the centrum is at about two thirds of centrum length as measured from the posterior end in BSPG 2011 I 117; in BSPG 2011 I 118, the neurocentral suture bulges slightly laterally in this area, so that the narrowest part in dorsal view is just behind mid-length of the centrum. The articular surfaces for the neural arch pedicles meet in the center of the vertebrae in BSPG 2011 I 117 and 118. In CMN 50791, which preserves the neural arch, both neural arch pedicles also meet on the floor of the neural canal, resulting in a ventrally narrow neural canal. In BSPG 2011 I 117, the posterior part of the neural canal floor is a trench-like depression in the centrum due to the dorsally elevated articular surfaces for the neural arch, with steep lateral boundaries that progressively open posteriorly and result in a “V”-shaped pattern. One neurovascular foramen penetrates the surface of the bone. In BSPG 2011 I 118, the dorsal surface posterior to where the articular surfaces of the neural arch meet is eroded, and neurovascular foramina are not evident. Anteriorly, the floor of the neural arch also widens in a V-shaped fashion, but its margins are less notably raised.

The neural arch of CMN 50971 shares many features with the isolated neural arch NHMUK PV R 16427 (Fig. 4). The neural canal opening widens dorsally in both specimens. This is also to be expected for BSPG 2011 I 117 and 118, based on the narrowing floor of the neural canal.

The bases of the transverse processes are preserved in both specimens. The processes are more ventrally than laterally directed, so that they overhang the lateral side of the neural arch in lateral view. Their anterior margin is placed far anteriorly on the neural arch, as it is ‘typical’ for anterior to mid-cervical vertebrae. Whereas the transverse process is anteroposteriorly narrow at its base in NHMUK PV R 16427, with the posterior margin being placed at about the mid-length of the neural arch, it is anteroposteriorly longer in CMN 50791, ending at approximately two-thirds of the length of the neural arch. The best-developed lateral lamina is the prezygodiapophyseal lamina (prdl) in both elements, which expands from the ventromedial edge of the prezygapophysis in slightly concave arch posteroventrally towards the anterior margin of the transverse process. Anteriorly, the short, but stout centroprezygapophyseal lamina (cprl) and the prezygodiapophyseal lamina border a transversely narrow, triangular prezygapophyseal centrodiapophyseal fossa of the transverse processes. On the posterior side of the transverse processes, a short posterior centrodiapophyseal lamina (pcdl) is present. In NHMUK PV R 16427, this lamina is developed only as a sharply defined ridge that extends from the posterior border of the transverse process in a gently concave arch first posterodorsally and then posteroventrally and ends on the lateral side of the neural arch pedicle some one-fourth of the length of the latter anterior to its posterior margin. In CMN 50791, the lamina is slightly better developed and extends from the transverse process traight posteriorly to end on the base of the neural arch pedicle a short way anterior to the posterior margin of the latter. In both cases, this lamina defines a deep posterior fossa underneath the transverse process. A true postzygodiapophyseal lamina (podl) is absent in both neural arches, but there is a low, broad ridge that curves anteroventrally from the lateral margin of the postzygapophysis that corresponds to this lamina. This ridge fades approximately halfway to the transverse process into the lateral side of the neural arch in NHMUK PV R 16427, whereas it reaches the posterior margin of the process as a slight lateral swelling in CMN 50791, thus defining a broad, triangular, but very shallow postzygapophyseal–centrodiapophyseal fossa (pocdf).

The prezygapophyses project far anteriorly in both specimens, with their articular surfaces completely extending beyond the anterior end of the centrum in CMN 50791. In both neural arches, the prezygapophyses are placed well lateral to the neural canal, but their angle of divergence is quite different. In NHMUK PV R 16427, both prezygapophyses diverge in an angle of approximately 90°, whereas the divergence is considerably narrower, at c. 65° in CMN 50791. The articular surfaces of the prezygapophyses are oval in outline, being longer than wide. They are slightly wider posteriorly in CMN 50791 and wider anteriorly in NHMUK PV R 16427. The facets face dorsomedially, and stand at an angle of approximately 130° towards each other in both specimens in anterior view. NHMUK PV R 16427 lacks a well-defined interprezygapophyseal laminae; the prezygapophyses are connected to each other anterior to the neural spine by a broad ridge, rather than a lamina. CMN 50791 bears two small flanges in front of the neural spine that project from the medial surface of each prezygapophysis over the opening of the neural canal and meet in an open suture. Centroprezygapophyseal fossae are poorly developed in both neural arches; the space between the centroprezygapophyseal lamina, the medial rim of the prezygapophysis and the rim of the neural canal is only very slightly concave, quite unlike the deep and well-defined fossae seen in many theropods in this area, including several specimens of indeterminate spinosaurids (see below).

The postzygapophyses are long and narrow in both CMN 50791 and NHMUK PV R 16427. In the former, they overhang the centrum posteriorly for about half the length of their articular surfaces. As the centrum is not preserved, nothing can be said about a possible overhang of the postzygapophyses in NHMUK PV R 16427, but the end of the postzygapophyses are only slightly posterior to the posterior tip of the neural spine, whereas they protrude far posterior from the spine in CMN 50791. The articular facets are oval in outline, being longer than wide, and stand at an angle of slightly more than 110° towards each other. Epipophyses are present on the dorsal surfaces of the postzygapophyses and are situated at the medial side of the latter. They are developed as robust, laterally inclined ridges with bluntly rounded posterior ends that slightly overhang the postzygapophysis posteriorly in NHMUK PV R 16427. In CMN 50791 the epipophyseal ridges are lower, more erect and stand in continuation of the spinopostzygapophyseal laminae. They taper posteriorly and end just above the posterior end of the zygapophyses. The epipophyses in these vertebrae are generally not as strongly developed as in *Spinosaurus aegyptiacus* (Stromer, 1915) and material we consider to represent a spinosaurid from Morocco, for which a generic classification cannot currently be made (BSPG 2006 I 57, CMN 41768, CMN 50790). A weakly developed laterodorsal edge extends anteriorly from the epipophyses over the lateral surface of the neural canal. This edge, which corresponds in position to the prezygoepipophyseal lamina in other theropods, is better developed in NHMUK PV R 16427, but does not reach the prezygapophysis anteriorly.

In NHMUK PV R 16427, the neural spine has a weak posterodorsal inclination. The neural spine is low in NHMUK PV R 16427, and only minor portions in the posterior part seem to be missing due to breakage. The neural spine is anterodorsally elongate, and its edge is thinnest in its mid-part. It has an unusual shape, its anterior half being especially low (only about half as high as the neural arch excluding the spine), with a straight dorsal margin, whereas the posterior half raises posterodorsally. The anterior margin of the neural spine is slightly thickened and has a slightly depressed anterior facing surface that seems to be a ligament groove. In this groove, parts of an ossified ligament attachment remain as a low, longitudinal ridge. Posteriorly, the spine broadens transversely, and there seems to have been a robust dorsal projection, which is broken off. CMN 50791 also shows an anterior facing groove in the lower part of the neural spine, which is laterally bound by spinoprezygapophyseal laminae (sprl) and also represents a ligament groove. CMN 50791 has an upright, rather elongate, transversely narrow and thus blade like neural spine, the dorsal end of which is broken off.

The posterior margin of the neural spine is flanked by right and left spinopostzygapophyseal laminae (spol). Between the laminae, a spinopostzygapophyseal fossa (spof) covers the posterior aspect of the spine. The fossa is narrow over its entire length in CMN 50791, but expands transversely between the postzygapophyses in NHMUK PV R 16427, and is also deepest in this area. The ventromedial aspects of the postzygapophyses have flange like medial laminae, which in some *Sigilmassasaurus* specimens partly close the neural canal dorsally (or the spof ventrally). In NHMUK PV R 16427, these flanges meet and appear to form an interpostzygapophyseal lamina. However, although the flanges meet in the midline, they are not fused; there is a clear suture between right and left flange. CMN 50791 also exhibits the interpostzygapophyseal lamina, but the flanges are fused.

The absolute positioning of these four elements is problematic, especially since only the type vertebra retains both centrum and neural arch. The most anterior element of these specimens is NHMUK PV R 16427. Several lines of evidence indicate that it is more anteriorly positioned than CMN 50791, including the better developed epipophyses, the more strongly diverging prezygapophyses, the shorter postzygapophyses, the anteroposteriorly shorter base of the transverse processes, and the weakly developed lateral lamination (see e.g., Charig & Milner, 1997). Furthermore, the neural spine shows a conspicuous step, with a rectangular anterior portion and a further dorsally expanded posterior part. This morphology is also found in an anterior mid-cervical (probably C4) of *Baryonyx* (Charig & Milner, 1997): Fig. 20C; though these authors identified the element as C5) and in C4–C6 of *Ichthyovenator* (BK10-18 to BK10-20). Thus, we tentatively identify this neural arch as C4. Nevertheless, the general similarity between NHMUK PV R 16427 and CMN 50791 indicate that they represent the same taxon. Characters supporting this hypothesis are the weakly developed epipophyses in both elements and an unusual arrangement of the laminae on the anterior end of the neural arch. In *Baryonyx* (NHMUK PV R 9951; Charig & Milner, 1997) and *Ichthyovenator* (BK 10-18, BK 10-21), the centroprezygapophyseal lamina is short and laterodorsally directed and meets the prezygodiapophyseal lamina from ventral in an almost right angle; together with the intraprezygapophyseal lamina these two laminae thus define the lateroventral, lateral, and dorsal borders of the centroprezygapophyseal fossa. In NHMUK PV R 16427 and CMN 50791, the centroprezygapophyseal lamina is less laterally directed and joins the prezygodiapophyseal lamina in a sharp angle to form a robust joint lamina that meets the prezygapophysis from ventral. Since the intraprezygapophyseal lamina is furthermore reduced to stout, low ridges, this very robust prezygapophyseal stalk lacks a clearly defined centroprezygapophyseal fossa. Some features, such as the unfused postzygapophyseal flanges, suggest that the specimen NHMUK PV R 16427 represents a younger individual than CMN 50791. This is also supported by the fact that the neural arch is isolated from its centrum, because separation of arch and centrum does not seem to be due to breakage.

CMN 50791 probably represents C6 (though an identification as C7 cannot be completely excluded). This interpretation is supported by the relative elongation of the vertebral centrum, with the ventral length-posterior height ratio (length measurements excluding anterior condyles) being approximately 1.45, which is very similar to C6 of *Ichthyovenator*. In contrast, the fifth cervical is considerably shorter (ratio of c. 1.05) and the seventh cervical relatively longer (c. 1.75) in the latter taxon. Further support comes from the lateral neural arch lamination. In *Ichthyovenator*, C6 lacks a postzygodiapophyseal lamina, and there is only a slight lateral swelling that extends from the anterior rim of the postzygapophysis anteroventrally and ends on the neural arch above the posterior half of the transverse process. In contrast, the swelling is more strongly developed in C7 and extends to the posterior end of the transverse process. CMN 50791 corresponds to the situation in C6.

As for the isolated centra BSPG 2011 I 117 and 118, the former corresponds very well to the morphology seen in the centrum of CMN 50791 and might thus represent the same vertebral position. The less pronounced ventral keel and the stronger offset of the articular facets in BSPG 2011 I 118 indicate that this centrum, although larger in overall size than BSPG 2011 I 117, represents a more anterior element. Thus, a positon as C5 seems likely.

### Posterior cervical vertebrae

In most posterior cervical vertebrae, with the exception of BSPG 2006 I 56 (see below), the width of the articular facets exceeds both the height and length of the centra, and transverse processes are elongate, stout and ventrally inclined. They become elevated progressively in more posterior positions, but without reaching a horizontal orientation, as it is the case in dorsal vertebrae.

Posterior cervical vertebrae are very similar to the ‘typical’ *Sigilmassasaurus* morphology, although the holotype of *S. brevicollis* is most probably a first dorsal vertebra (see below). Here, we recognize seven vertebrae as posterior cervicals: BSPG 2006 I 53, BSPG 2006 I 56, BSPG 2011 I 115, BSPG 2011 I 116, CMN 41774, CMN 41790, and CMN 41856. As noted above, the following descriptions will mainly be based on the new specimens from the collections of the BSPG; for detailed descriptions of the CMN specimens see McFeeters et al. (2013).

BSPG 2006 I 53 is a large partial vertebra, which preserves most of the centrum but the distal part of its left parapophysis. Parts of the neural arch are preserved, such as the left prezygapophysis, and the medial part of its transverse process. BSPG 2006 I 56 preserves the right pre- and postzygapophyses and the base of the right transverse process. The neural spine is broken but partly preserved, but all processes of the left side of the neural arch are broken away. There is a large piece of bone missing on the right side of the dorsal part of the anterior condyle. A mark with a smooth surface and squared outline penetrates the bone in this area. The area of broken bone is lighter in color than other broken parts, which suggest the damage is relatively fresh. Overall, the morphology of the mark is consistent with the size and shape of a small chisel or hammer, and we propose the damage occurred during excavation of the specimen. This condition is unlike the mark on the anterior condyle of BSPG 2011 I 118 (see above), which seems to be bite mark. Another chisel-mark can be seen in the floor of the pneumatic invasion connected with the left central pneumatic foramen of the same specimen. Here, a squared, smooth-walled mark with decreasing depth is evident. BSPG 2011 I 115 is virtually complete, with only half of the right prezygapophysis and the tip of the right postzygapophysis being missing. However, this specimen also shows a chisel mark; it has a squared hole virtually identical in appearance to the one described for BSPG 2006 I 56. The abundance of anthropogenic damage to the specimens described in this study shows that specimens often seem to be excavated without appropriate caution. In BSPG 2011 I 116, the right prezygapophysis is eroded and most of the left transverse process is missing. CMN 41774 lacks its left postzygapophysis; CMN 41790 preserves only parts of the left transverse process, left prezygapophysis and neural spine. CMN 41856 lacks both postzygapophyses and most of its spine.

We can tentatively identifiy the positions of these vertebrae mainly on the morphology of the centrum (especially the development of the ventral keel) and the orientation of the transverse processes, and in comparison with the complete cervical vertebral column of *Ichthyovenator* (BK10-16–BK10-24). BSPG 2006 I 56 is the most anterior of these posterior cervicals, and would thus correspond to C8, followed by BSPG 2011 I 115, CMN 41774 and CMN 41856, which represent C9. BSPG 2006 I 53 and BSPG 2011 I 116 are ultimate cervicals (C10). CMN 41790 can clearly also be referred to *Sigilmassasurus* (McFeeters et al., 2013), but its morphology does not correspond exactly to any of these other vertebrae, possibly due to restorations of this element carried out prior to its purchase by the CMN (B McFeeters, pers. obs., 2013). This element is most similar to BSPG 2006 I 56 and BSPG 2011 I 115 and might thus also represent a vertebra from the transition between the mid-cervicals to the posterior cervicals.

BSPG 2006 I 56 is an important specimen, as it shows transitional features between the mid-cervical vertebrae described above, and the ‘typical’ *Sigilmassasaurus brevicollis* morphology observed in the holotype (CMN 41857) and referred material (Fig. 5). The specimen is strongly opisthocoelous, with the anterior articular facet exhibiting a rimmed edge, as it is found in many megalosauroids (Carrano, Benson & Sampson, 2012). The posterior articular facet is reniform. As in other ‘typical’ *Sigilmassasaurus* vertebrae, the anterior condyle bears a median tuberosity and intercentral articulations are wider than high (the width–height ratio of the anterior facet is 1.67, the one of the posterior facet is 1.52), but in contrast to these the centrum is longer than it is wide. The width–height ratio is intermediate between proposed mid-cervical vertebrae like BSPG 2011 I 118 with a posterior width–height ratio of 1.26, and ‘typical’ *Sigilmassasaurus* vertebrae like BSPG 2006 I 54 with a posterior width–height ratio of 1.71. The length–width ratio is also intermediate (BSPG 2011 I 118: 1.83; BSPG 2006 I 56: 1.14; BSPG 2011 I 116: 0.8; ratios based on dorsal central length including the anterior condyle and width of the posterior articular facets). The posterior articular end is set at an angle of slightly less than 90° towards the long axis of the centrum, so that the latter slopes anterodorsally when the posterior end is oriented vertically. Furthermore, the dorsal side of the centrum is slightly shorter than the ventral side, so that the anterior articular surface is angled slightly dorsally in comparison to the posterior end.

The parapophyses are massive processes as in both the holotypes of *Sp. maroccanus* and *S. brevicollis*, but more lateroventrally directed than in the former. The ventral keel is more pronounced than in mid-cervical vertebrae, but less prominent than in ‘typical’ *Sigilmassasaurus* vertebrae. The keel is similar in shape and extent to the structure found in BSPG 2011 I 117, but more sharply defined. Importantly, the keel fades posteriorly into a transversely broad and ventrally elevated triangular platform, as in the proposed mid-cervical vertebrae, including the holotype of *Sp. maroccanus* (CMN 50791). The triangular platform is less prominent, and intermediate in size between mid-cervical vertebrae and posterior cervicals, as for instance BSPG 2011 I 116. In contrast to the mid-cervicals and many of the other posterior cervicals, this vertebra lacks the intense rugose pattering on the platform and along the anterior rim; since this vertebra is rather small in comparison with most of the elements dealt with here, this is probably due to immaturity. The ventral side lateral to the keel faces mainly ventrally, in contrast to the gently dorsolaterally sloping ventral side in the mid-cervical vertebrae. In lateral view, the ventral side is straight, which also contrasts with the mid-cervicals, in which the ventral margin is anteroposteriorly concave anterior to the posterior platform. Due to the shortening of the centrum and the resulting relative elongation of the parapophysis, the lateral pneumatic foramen is placed dorsal to the latter, rather than posterodorsal, as in the mid-cervicals. The pneumatic foramina are large and anteroposteriorly elongate, less so than in the mid-cervicals, yet more elongate than in more posteriorly positioned specimens. The foramina are oval in outline, with less angled anterior end posterior margins, and lead into large internal cavities, as in the mid-cervicals. Posteroventrally, the pneumatic foramen is bordered by a stout, rounded ridge extending from the parapophysis posterodorsally. This ridge continues on the lateroventral side of the centrum as a rounded edge that separates the lateral from the ventral side, very similar in position and orientation to that seen in mid-cervicals, but less marked. The lateral bulge above the pneumatic foramen and the corresponding dorsal depression between this bulge and the neurocentral suture are also present, but relatively smaller than in the mid-cervicals.

Although the neural arch of this vertebra is preserved in articulation with the centrum, the neurocentral suture is open and clearly visible. The attachment of the neural arch is extensive and reaches down to almost half the height of the lateral side of the centrum. As in the mid-cervical vertebrae, the left and right pedicles meet in the midline at the floor of the neural canal, though the contact is relatively more anterior than in the former. Posteriorly, there is a large, posteriorly opening triangular area between the pedicles that houses three larger foramina in its anterior part, similar to the situation in BSPG 2011 I 117. The neural canal is very large, being slightly wider than high.

The relatively steeply ventrally inclined remains of the broken transverse processes are consistent with the interpretation that BSPG 2006 I 56 occupies the anterior-most position of all vertebrae considered in this section and further confirm its intermediate position between the mid-cervical vertebra that represents the holotype of *Sp. maroccanus* and the unambiguously posterior cervical position of the other vertebrae. The broken bases of the transverse processes show that a single, very robust centrodiapophyseal lamina was present on each side, as in other *Sigilmassasaurus* specimens. This lamina extends ventrolaterally from the base of the neural arch to the ventral side of the transverse process and is broad and anteroposteriorly convex ventrally, without any indication of a separation of an anterior and posterior centrodiapophyseal lamina and thus without any centrodiapophyseal fossa. The prezygapophyses are widely spaced, strongly divergent, and not interconnected by an interprezygapophyseal lamina. They sit on anterodorsally expanded stalks and are elongate oval in outline, their anterior end being approximately flush with the anterior margin of the anterior convexity of the centrum. The articular surface is very slightly convex anteroposteriorly. Due to the shortness of the neural arch, the posterolateral rim of the prezygapophyses is placed at the level of the anterior end of the neural spine and overhangs the anterior margin of the transverse process; it forms a posterolaterally expanded lip on the neural arch. Prezygapophyseal stalks are anteriorly broad, have a laterally positioned cprl and lack centroprezygapophyseal fossae, as in both the holotypes of *Sp. maroccanus* and *S. brevicollis*. The centroprezygapophyseal lamina meets the robust prezygodiapophyseal lamina approximately half way between the centrum and the prezygapophysis, and together the two laminae form the robust stalk, which meets the prezygapophysis from lateroventral. A small, but deep, cone-shaped recess is present between the two laminae underneath the anterior side of the transverse process and opens anterolateroventrally. Posteriorly, the transverse process has a sharp posterior margin that corresponds to the lower part of the postzygodiapophyseal lamina, which is otherwise interrupted between the lateral margin of the postzygapophysis and this margin. Together with the centrodiapophyseal lamina and the short and laterally oriented centropostzygapophyseal lamina, this margin defines an oval, very deep recess below the posterior base of the transverse process, which opens posterolaterally.

The postzygapophyses are large and anteroposteriorly elongate. The articular facet is slightly concave anteroposteriorly and overhangs the centrum posteriorly for approximately half its length. The facet has a straight to slightly concave medial margin, a strongly convex anterolateral margin, and an angular posterior margin, being pointed posteriorly in its medial third. Epipophyses are missing, as in the holotype of *S. brevicollis*, but the base of the spinopostzygapophyseal lamina on the dorsal surface of the postzygapophysis has a slightly swollen appearance, which is interpreted as the remnant of reduced epipophyses. The postzygapophyses lack an interpostzygapophyseal lamina, but small, medially projecting flanges are present at the base of their stalks, as observed also in posterior cervical and cervicodorsal vertebrae. A prezygoepipophyseal lamina or ridge is not present, but the area between the lateral margins of the pre- and postzygapophyses is dorsoventrally convex, whereas there is a very shallow depression on the dorsolateral surface of the base of the transverse process and a smaller, more marked depression dosally between the neural spine, the spinopostzygapophyseal lamina and the roof of the neural arch.

The neural spine is anteroposteriorly very short and spike-like, as seen in many *Sigilmassasaurus* specimens. However, the spine generally seems to be subject to relatively great positional variance (see neural spine descriptions across axial positions). No spinoprezygapophyseal laminae are present, but the anterolateral margin of the spine is connected to the medial side of the prezygapophyses by a stout, diverging edge, resulting in a broad, anteriorly facing, triangular surface at the base of the spine. No medial ridge for the attachment of the interspinal ligament is present in this area, though this might be due to poor preservation. Posteriorly, the spinopostzygapophyseal laminae are well-developed and stout, but low, extending almost horizontally from the dorsal surface of the postzygapophysis anteromedially to the posterolateral margin of the neural spine. Between the laminae, a low ridge extends over the posterior surface of the spine, but there is no deep spinopostzygapophyseal fossa, which is at least partially be due to the lack of an interpostzygapophyseal lamina.

In summary, there is substantial anatomical evidence that BSPG 2006 I 56 is a specimen in transition between the elongate morphology of proposed mid-cervicals of *Sigilmassasaurus brevicollis*, and the extreme broad appearance of posterior cervicals. The trends described and the transitional morphology of BSPG 2006 I 56 are not particularly unusual for theropod vertebrae. Several other taxa go through major morphological transitions throughout the axial and especially the cervical series, as for example *Baryonyx* and *Ichthyovenator* (see discussion).

Posterior cervical vertebrae of *Sigilmassasaurus* are strongly opisthocoelous, and a conspicuous rim surrounds the articular facets in these vertebrae. The anterior articular facet has a distinct centrally placed, bump-like elevation, the anterior median tuberosity. The median tuberosity is expressed to a different degree in the specimens; in BSPG 2006 I 53 and BSPG 2006 I 56 it is very pronounced, while it is more subtle in BSPG 2011 I 115, where it is best seen in ventral view.

All of these vertebrae show very broad intercentral articulations, with width–height ratios of the anterior articular facets exceeding 1.7. The vertebrae are also wider than they are long. In BSPG 2006 I 53, the width of the anterior articular facet exceeds the length of the centrum (without the anterior condyle) by 72.5%. The centrum is slightly anterodorsally angled if the posterior articular end is held vertically in C9 (e.g., BSPG 2011 I 115, Fig. 6), but only very slightly so in the last cervical vertebra. In all vertebrae, the dorsal side of the vertebral centrum is slightly shorter than the ventral side, so that the anterior facet is inclined anterodorsally to similar, but slightly variable extent across the specimens, which we account to different axial positions within the posterior cervical series and individual variation. The posterior articular facets of the centra are strongly concave and reniform in outline. The edge of the posterior articular facet is straight in ventral view, but dorsally the rim slightly curves towards the opening of the neural canal. This, again, is variable in its extent across specimens.

**Figure 8:**
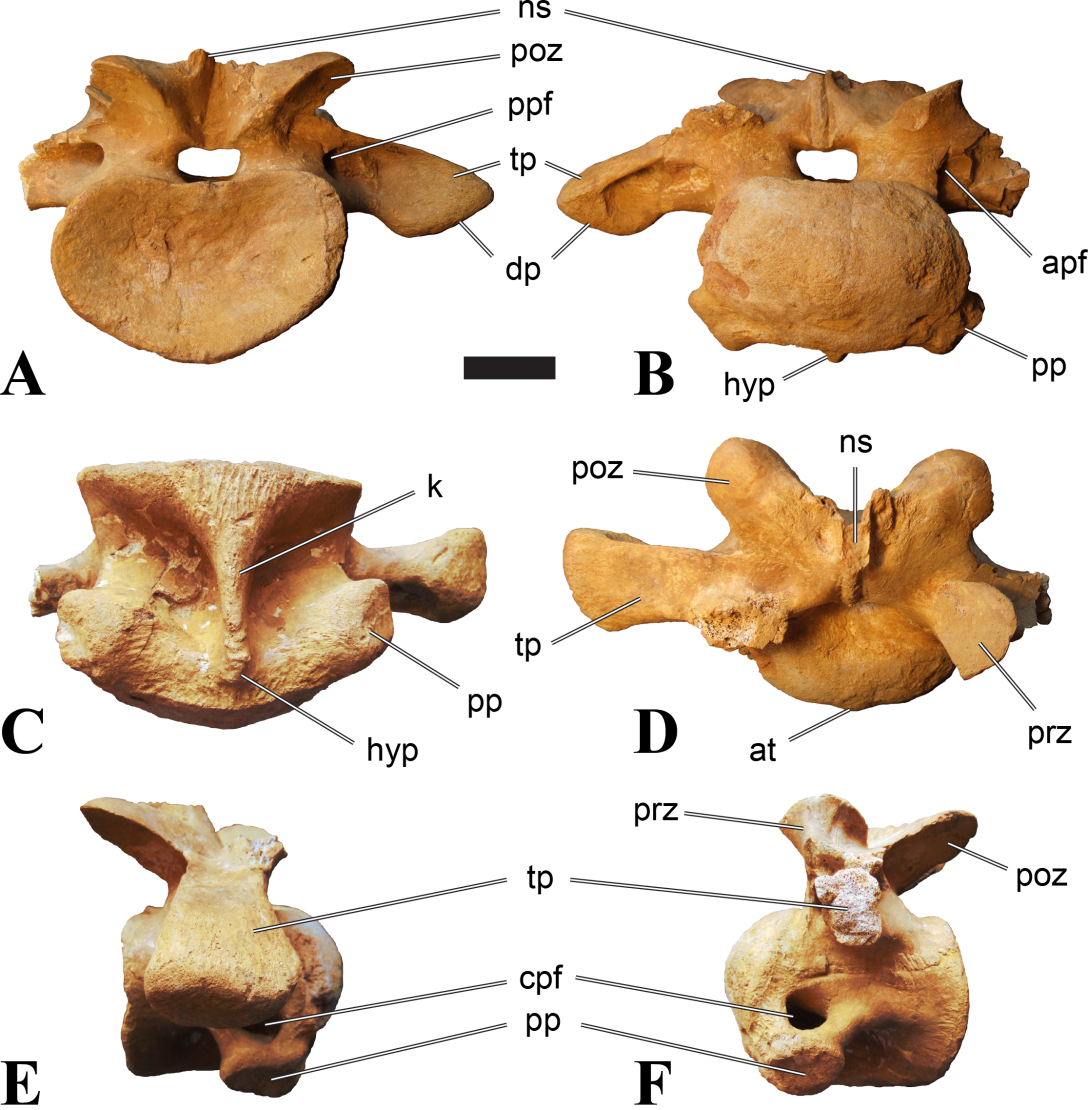
BSPG 2011 I 116, posterior cervical vertebra (C10) of *Sigilmassasaurus brevicollis*. (A) posterior view; (B) anterior view; (C) ventral view; (D) dorsal view; (E) right lateral view; (F) left lateral view. Abbreviations: apf, anterior pneumatic foramen of the prezygodiapophyseal fossa; at, anterior medial tuberosity; cpf, central pneumatic foramen; dp, diapophysis; hyp, hypapophysis; k, keel; ns, neural spine; poz, postzygapophysis; pp, parapophysis; ppf, posterior pneumatic foramen of the postzygodiapophyseal fossa; prz, prezygapophysis; tp, transverse process. Scale bar equals 5 cm.

Well-developed ventral keels are present in all posterior cervical vertebrae and expand posteriorly into a small triangular platform. There is a progressive change from less prominent keels in more anterior specimens (such as BSPG 2006 I 56) to very pronounced structures in more posterior elements, although there also seems to be some individual variation in the development of this structure. BSPG 2011 I 115, CMN 41774 and CMN 41856 have low but distinct keels with a straight ventral edge. In BSPG 2006 I 115, the keel is positioned slightly off the midline and placed more on the right side of the specimen. Anteriorly, the keel becomes progressively lower and merges into the broad, rugose area between the parapophyses, which are notably ventrolaterally directed in these vertebrae. In anterior view, the keels might be visible as small, triangular ventral prominences. The probable last cervical vertebrae BSPG 2006 I 53 (Fig. 7) and 2011 I 116 (Fig. 8) have relatively high keels that become deeper anteriorly, but lack a distinct hypapophysis. Instead, the keel lowers more or less abruptly between the parapophyses, which, in these vertebrae are connected by a thick, rounded transverse ridge anteriorly. In these specimens, the keel is visible in anterior view and extends ventrally to or even beyond the level of the ventral margin of the parapophyses. There is some variation in the development of the ventral keel in these vertebrae: whereas it is deeper in BSPG 2011 I 116, it is more robust and becomes especially robust anteriorly in the slightly larger BSPG 2006 I 53. Likewise, the ventral edge of the keel is straight in lateral view in BSPG 2006 I 53, but slightly convex in BSPG 2011 I 116.

**Figure 9:**
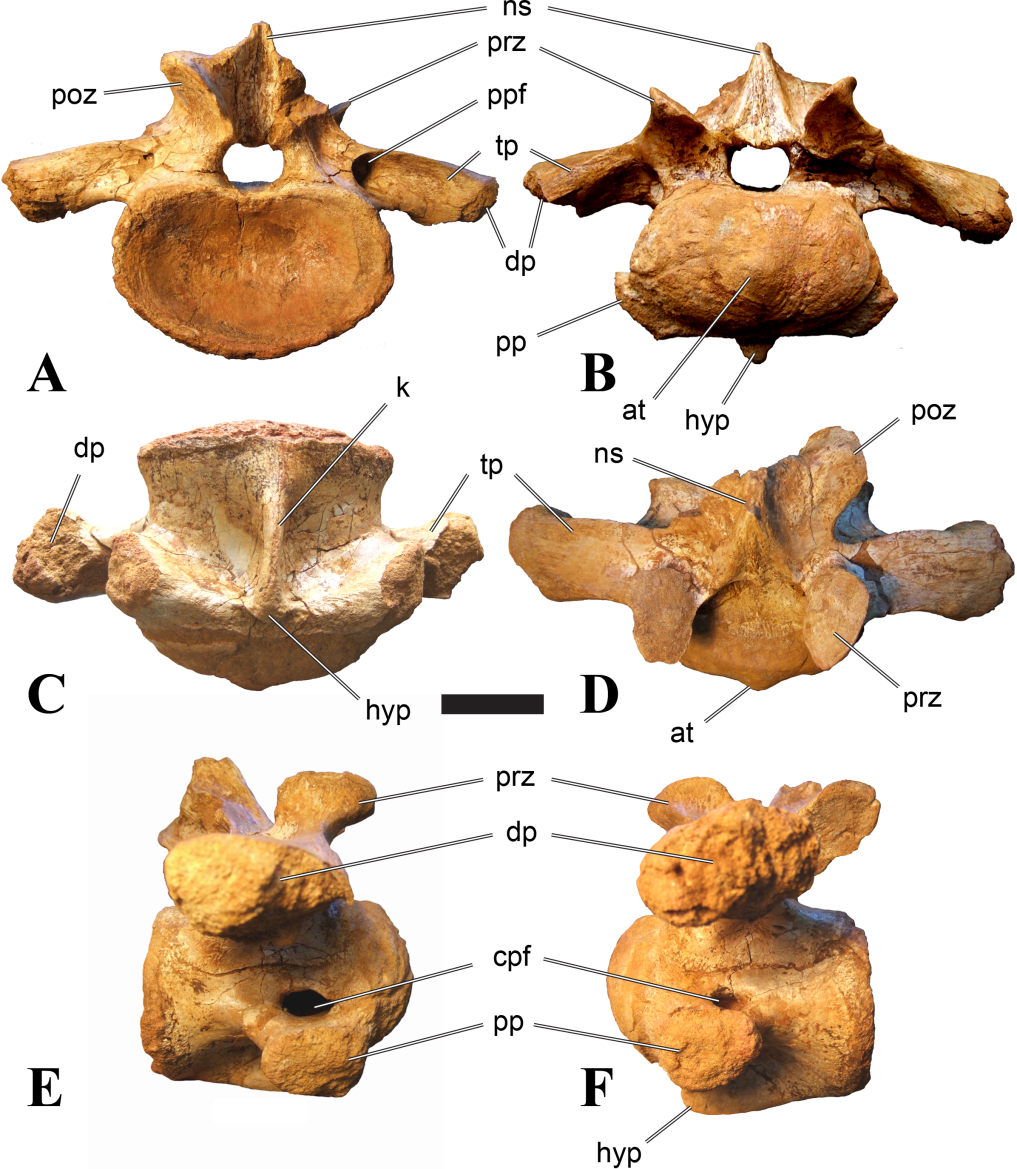
BSPG 2006 I 54, anterior dorsal vertebra (D1) of *Sigilmassasaurus brevicollis*. (A) posterior view; (B) anterior view; (C) ventral view; (D) dorsal view; (E) right lateral view; (F) left lateral view. Abbreviations: at, anterior medial tuberosity; cpf, central pneumatic foramen; dp, diapophysis; hyp, hypapophysis; k, keel; ns, neural spine; poz, postzygapophysis; pp, parapophysis; ppf, posterior pneumatic foramen of the postzygodiapophyseal fossa; prz, prezygapophysis; tp, transverse process. Scale bar equals 5 cm.

**Figure 10:**
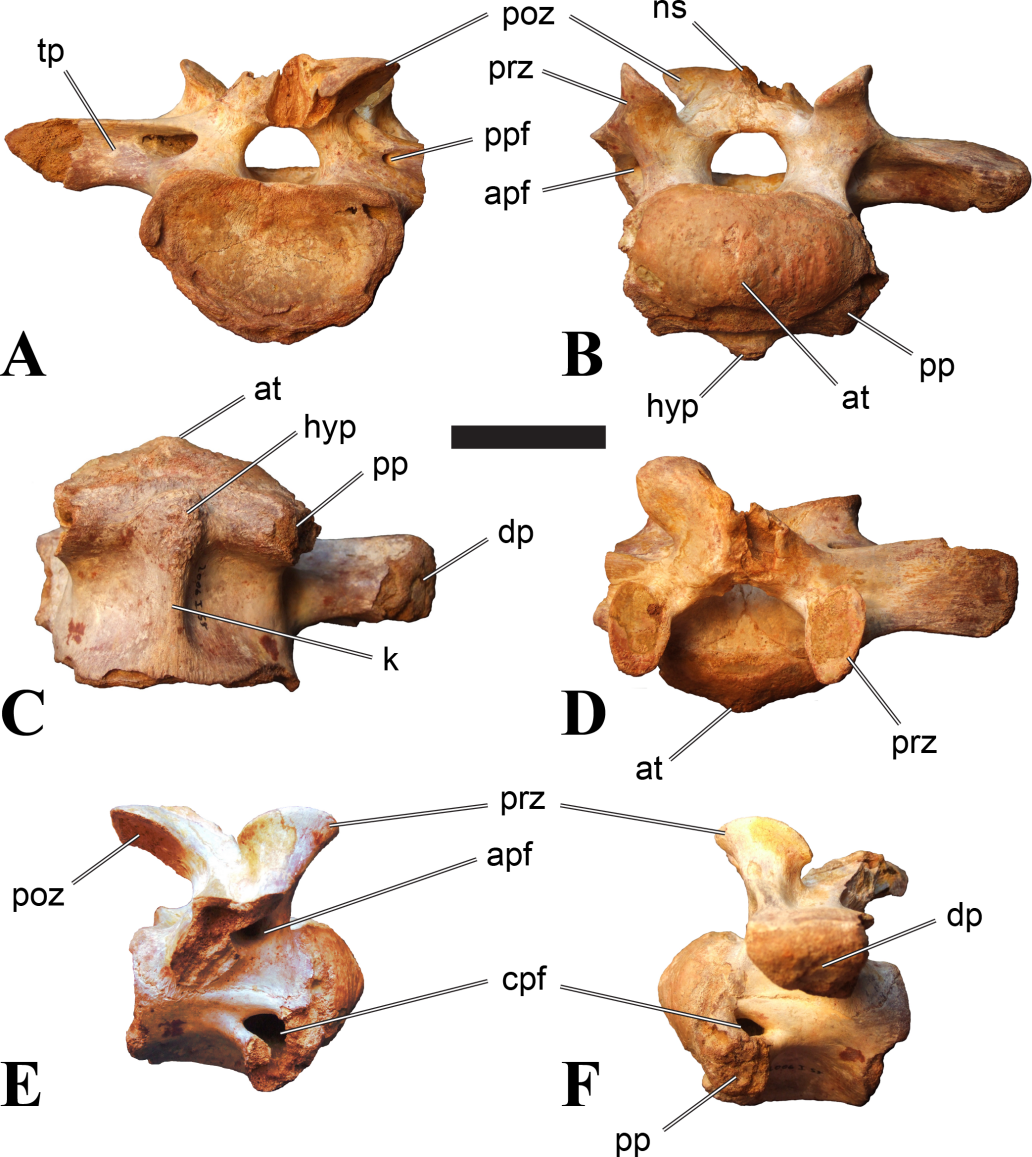
BSPG 2006 I 55, anterior dorsal vertebra (D1) of *Sigilmassasaurus brevicollis*. (A) posterior view; (B) anterior view; (C) ventral view; (D) dorsal view; (E) right lateral view; (F) left lateral view. Abbreviations: apf, anterior pneumatic foramen of the prezygodiapophyseal fossa; at, anterior medial tuberosity; cpf, central pneumatic foramen; dp, diapophysis; hyp, hypapophysis; k, keel; ns, neural spine; poz, postzygapophysis; pp, parapophysis; ppf, posterior pneumatic foramen of the postzygodiapophyseal fossa; prz, prezygapophysis; tp, transverse process. Scale bar equals 5 cm.

**Figure 11:**
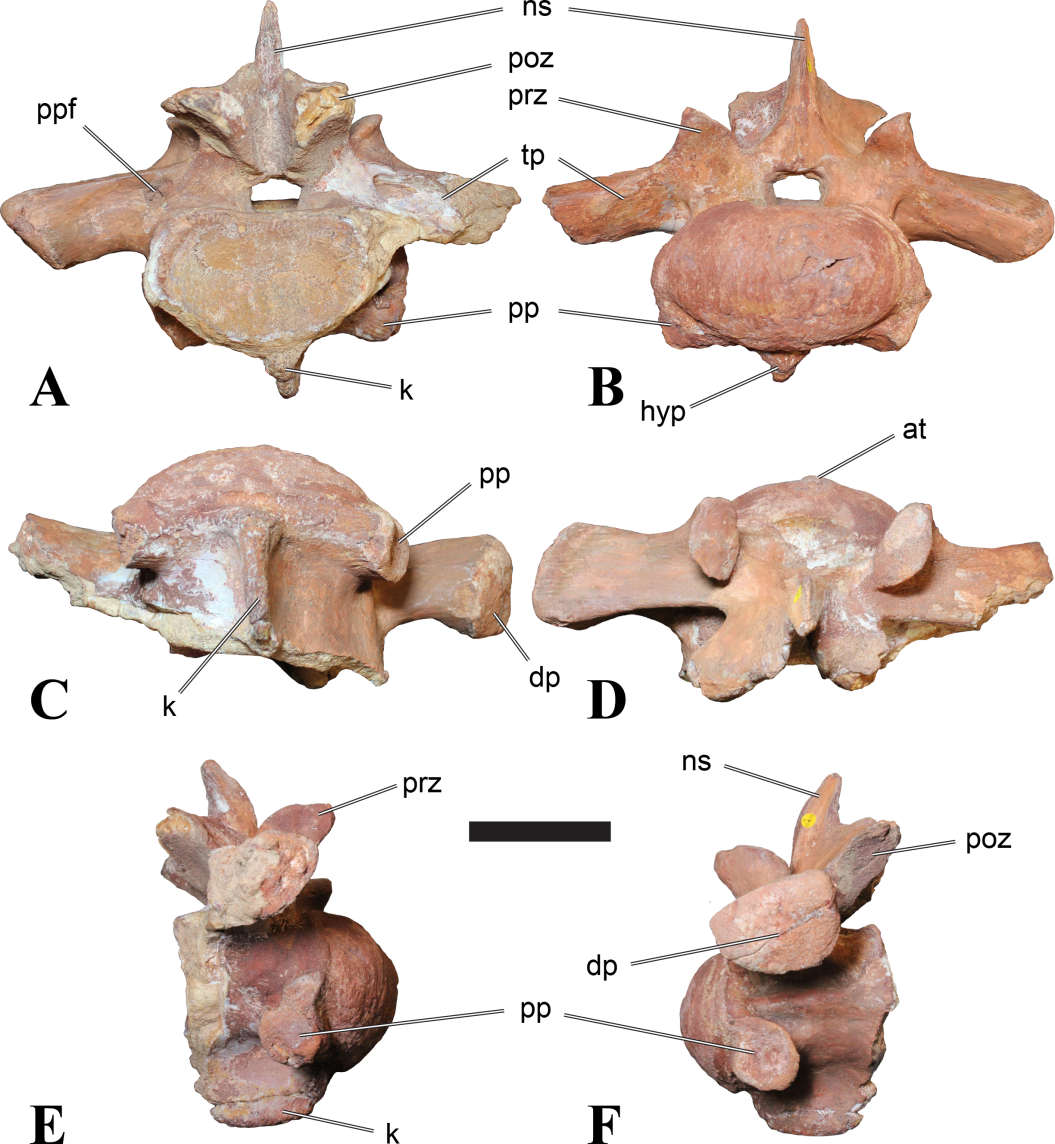
NHMUK PV R 16434, anterior dorsal vertebra (D1) of *Sigilmassasaurus brevicollis*. (A) posterior view; (B) anterior view; (C) ventral view; (D) dorsal view; (E) right lateral view; (F) left lateral view. Abbreviations: at, anterior medial tuberosity; dp, diapophysis; k, keel; ns, neural spine; poz, postzygapophysis; pp, parapophysis; ppf, posterior pneumatic foramen of the postzygodiapophyseal fossa; prz, prezygapophysis; tp, transverse process. Scale bar equals 5 cm.

A broad but shallow fossa is found on the ventral side of the centrum, and expands between the ventral keel medially, the parapophysis and transverse ridge anterolaterally, and the posterior connection of the parapophysis posterolaterally. The fossa is deeply excavated in more posterior positioned vertebrae, and this is emphasized by the high ventral keel. The depth decreases with lower keels in anterior-more posterior cervicals.

The parapophyses are robust processes that are situated on the anteroventral part of lateral side of the centrum and project ventrolaterally. Their anterior surface is confluent with the rim around the anterior facet. The extent to which the parapophyses point ventrolaterally differs with the different vertebral positions. The strongest ventrolateral orientation is found in the possible C9, such as BSPG 2011 I 115, in which the divergence of the parapophyses is slightly less than 90°, but also in the CMN specimens (McFeeters et al., 2013). In the probably ultimate cervical BSPG 2011 I 116, the angle of divergence between the parapophyses is slightly wider at 95–100°. The parapophyses are relatively long and project further than for example in *Baryonyx* (Charig & Milner, 1997), but not further than in *Allosaurus* (e.g., UMNH VP 8358, 8365, 8488, 8489, 10192; all posterior cervical vertebrae).

Parapophyseal articular facets are generally concave, both anteroposteriorly and dorsoventrally. The concavity is more pronounced in C9 than in C10. The outline of the parapophyseal facets is oval to triangular in the presumed C9. One edge of the triangle points posteriorly and continues onto the centrum as a thick ridge that borders the lateral pneumatic foramen ventrally. In these vertebrae, the articular facets of the parapophyses are longer anteroposteriorly than high dorsoventrally. BSPG 2011 I 116 shows an intermediate state in the outline of the facet between the more triangular shape seen in BSPG 2011 I 115 and the high oval shape in the first dorsal vertebra, in that the facet is generally oval, but has a slightly posterodorsally expanded corner. As in the more anterior vertebrae, the posterior end of the parapophysis is connected to the centrum by a broad ridge, from which a rounded edge separating the lateral from the ventral side extends posteriorly. This edge becomes broader and more rounded and thus less marked in more posterior elements.

A single, undivided lateral pneumatic foramen opens directly above the parapophysis in all specimens. Foramina can be symmetrically developed, as in BSPG 2011 I 116, or asymmetrically as in BSPG 2011 I 115, in which the right foramen is much smaller in size. In most vertebrae, the pneumatic foramina are large and oval to triangular (pointed posteriorly) in outline, but BSPG 2006 I 53 has only a small, slit-like foramen on the right side and a foramen that is no larger than a neurovascular foramen on the left side. Features related to skeletal pneumaticity seem to be highly variable on an intraspecific level. The number of foramina is for example known to vary between individuals of *Acrocanthosaurus* (Harris, 1998) and within the cervical vertebral column in *Aerosteon* Sereno et al., 2008. The transition from an anteroposteriorly elongate, oval shape to a more oval to subcircular shape can be observed in other megalosauroid theropods, such as *Eustreptospondylus* (Sadleir, Barrett & Powell, 2008), as well.

A lateral bulge that runs parallel and ventral to the neurocentral suture is only present in BSPG 2006 I 53 among the posterior cervical vertebrae. This bulge divides the lateral depression on the centrum into a dorsal part on or above the neurocentral suture, and a ventral part that is more or less entirely occupied by the lateral pneumatic foramen. McFeeters et al. (2013) noticed the same feature on the holotype of *Sigilmassasaurus brevicollis* and termed it lateral bulge of the centrum. It is well-developed and marked by an especially deep dorsal depression on the neurocentral suture in BSPG 2006 I 53. In the other vertebra that probably occupies the same vertebral position, BSPG 2011 I 116, this bulge is only hinted at, and no dorsal depression is present. The neurocentral suture is well visible and not fully closed in even the largest specimens; none of the specimens shows even partial fusion and obliteration of the suture. In all specimens, the neurocentral suture reaches far onto the lateral side of the centrum to about half of its height (excluding the ventral keel). The neural arch has two pedicels that enclose the neural canal. As in the more anterior vertebrae, the pedicles meet each other in the midline of the neural canal anteriorly, thus forming most of the anterior part of the floor of the neural canal. The posterior part of the floor of the neural canal between the ventromedial borders of the neural arch pedicles is penetrated by several basivertebral foramina, which are mostly aligned in a small, elongate, trench-like depression.

The neural canal is large and wider than high in all specimens. The shape of the neural canal is oval in most specimens, though its dorsal margin is convex in some vertebrae, most notably 2011 I 116, resulting in a heart-shaped outline in anterior view.

The transverse processes merge to the lateral sides of the neural arch directly above the centrum. They are directed ventrolaterally at an angle of approximate 40–55° in the more anterior posterior cervicals BSPG 2006 I 56, BSPG 2011 I 115, CMN 41774, CMN 41790, and CMN 41856. This tilt decreases to an angle of c. 30° in the ultimate cervical BSPG 2011 I 116. Toward their distal ends, the transverse processes are gently ventrally curved, and their dorsal surface is slightly tilted anteriorly. Distally they expand anteroposteriorly, best seen in dorsal or ventral view. Whereas the posterior margin of the transverse process is straight, but slightly posterolaterally directed, this expansion is mainly defined by a rounded expansion of the anterior margin. This rounded anterior expansion is most marked in the more anterior vertebrae (e.g., BSPG 2011 I 115), but becomes less notable in the probable C10 BSPG 2011 I 116. The dorsal length of the transverse process is longer than the ventral length, resulting in a ventral to ventrolateral orientation of the diapophyses. In specimens that preserve the diapophyses, the facets show a roughly triangular outline and are flat.

The transverse processes are massive, and neural arch lamination seems to be largely reduced in *Sigilmassasaurus*. The prezygodiapophyseal laminae (prdl) and postzygodiapophyseal laminae (podl) are developed as robust, transversely oriented ridges, which form the anterodorsal and posterodorsal margin of the transverse process. They thus delimit the dorsal surface of the transverse processes, which is flat and table-like. The prezygodiapophyseal lamina joins the stout centroprezygapophyseal lamina at the anterior base of the transverse process in all vertebrae, and the joint laminae meet the central part of the ventral surface of the prezygapophysis from ventrolateral. The postzygodiapophyseal lamina shows somewhat more variation. In the anteriormost of these vertebrae, such as BSPG 2006 I 56 and BSPG 2011 I 115, there is only a small swelling on the lateral side of the neural arch anteroventral to the postzygapophysis, which is discontinuous with the ridge that forms the posterodorsal margin of the transverse process. In the probable ultimate cervical BSPG 2011 I 116, this swelling is expanded into a lateral ridge that connects the postzygapophysis with the posterodorsal margin of the transverse process. Both the lateral extensions of the prezygodiapophyseal and postzygodiapophyseal lamiane become lower distally, where they merge into the massive articular end of the diapophysis. A cross-section of the middle part of the transverse process would thus result in a “T”-shape, due to the medially narrow ventral ridge and the wide dorsal surface of the transverse process. A prominent ridge extends from the ventral side of the transverse processes to the lateral side of the vertebrae, and connects to the centrum directly above the neurocentral suture. This ridge represents the joint centrodiapophyseal laminae. In the posterior cervicals, the ventral ridge remains unbifurcated as a single “centrodiapophyseal lamina,” which we consider to be an autapomorphy of Sigilmassasaurus, as it is not present in other spinosaurids, such as *Baryonyx* (Charig & Milner, 1997) and *Ichthyovenator*. In the more anterior vertebrae, such as BSPG 2011 I 115, the ventral side of this ridge is convex, but in BSPG 2011 I 116, it is almost flat ventrally. There is a stout posterior ridge that extends from the proximal part of the central ventral side of the transverse process posteriorly to join the pedicle of the neural arch at the level of the dorsal margin of the centrum; this ridge probably corresponds to the posterior centrodiapophyseal lamina. In all specimens, the anterior sides of the transverse processes display prezygapophyseal centrodiapophyseal fossae (prcdf), which are open ventrally in their lateral part, being bound posteriorly by the joint centrodiapophyseal laminae, dorsally by the prdl, and anteriorly by the centroprezygapophyseal lamina (cprl). The centroprezygapophyseal lamina, which usually connects the prezygapophysis with the rim of the anterior articular facet (Wilson, 1999), is more laterally than anteriorly directed in *Sigilmassasaurus*, as already seen in mid-cervicals like CMN 50791. The centroprezygapophyseal lamina is placed slightly posterior to the anterior rim of the centrum and extends on the lateral aspect of the pedicle of the prezygapophysis and eventually curves onto the transverse process, where it meets the prezygodiapophyseal lamina. The prcdf extends over most of the anterior side of the transverse process below the lateral extension of the prezygodiapophyseal lamina and becomes more distinct medially. At the base of the transverse process, it leads into a depression that penetrates the lateral side of the prezygapophyseal pedicle, and which is developed as a deep foramen in several specimens. The foramina are slit-like in most specimens and of various sizes. There is considerable variation both in the size and depth of these foramina, sometimes from one side of the vertebra to the other. This variation is apparently not ontogenetic, as in BSPG 2006 I 56, interestingly one of the smallest vertebrae, the foramina are largest. The foramina are hidden by the centroprezygapophyseal lamina in anterior view.

On the posterior side of the transverse processes, postzygapophyseal centrodiapophyseal fossae (pocdf) are present. The pocdf expands over the medial three-fourth of the posterior side of the transverse processes. A medial part, on the neural arch, and a lateral part, on the transverse process, can be distinguished and is separated by the posterior pneumatic foramen of the transverse process. The medial part is a gentle and shallow depression anterolateral to the base of the postzygapophysis. It is variably developed in the posterior cervicals: it is clearly distinct as a depression in BSPG 2006 I 116, CMN 41774, and CMN 41856, but in BSPG 2007 I 56, the area between the posterior foramen and the base of the postzygapophysis is flat and not or only very slightly depressed. In BSPG 2011 I 115, a narrow and very shallow depression is present on the right side, but the left side is flat. Whereas the fossa, or the flat area anterolateral to the postzygapophysis faces posterolaterally in the more anterior vertebrae, it faces posteriorly and is largest and deepest in the last cervical, BSPG 2011 I 116. The depression on the posterior side of the transverse process is generally better developed and becomes more marked medially towards the opening of the pneumatic foramen. In *Sigilmassasaurus*, the pneumatic invasions of the transverse processes seem to be generally larger on the posterior side than anteriorly. The foramen is bordered dorsally by the continuation of the postzygadiapophyseal lamina. Medially, a ventrally flexing branch of the postzygodiapophyseal lamina curves around back onto the transverse process to border the medially penetrating posterior foramen from all but the lateral side. The foramina are usually large and deep, penetrating the posterior base of the transverse process from posterolateral.

In BSPG 2006 I 53, the partial breakage of the transverse processes allows detailed study of the pneumatic features of the transverse processes. On the right side, anterior and posterior foramina of the transverse processes lead into a medial chamber that invades the ventral part of the right neural arch pedicle. The right and left cavities connect internally to a single large chamber. On the left side, however, both cavities remain separated by a thin septum. Such a septum is clearly not developed on the right side, as the medial floor of the connected pneumatic cavity has a smooth texture. On the left side of BSPG 2006 I 53, the prezygapophyses is preserved, and no connection in the top of the chamber indicates any pneumatization of the prezygapophyses. Also, no channel opens toward the (missing) postzygapophysis, indicating that the latter was not pneumatized. A similar situation seems to be present in BSPG 2011 I 116, in which the anterior and posterior pneumatic recesses of the transverse process are confluent on the left, but apparently not on the right side.

In all specimens, the prezygapophyses are widely spaced, being placed far lateral to the neural canal. They are generally developed as anterodorsally projecting processes. They are placed anterodorsal to the transverse process, overhanging the latter only with their anterior third. In all posterior cervicals with the exception of the most anterior element BSPG 2006 I 56, the anterior margin of the prezygapophysis is placed above the anterior end of the centrum, posterior to the anterior convexity of the centrum. In the other specimens, the prezygapophyses are either anteroposteriorly oval or roughly spade shaped, being wider posteriorly. More anterior vertebrae, such as BSPG 2006 I 56, BSPG 2011 I 115 and CMN 41774 have anteroposteriorly elongate, prezygapophyses that are considerably longer than wide. In the ultimate cervicals BSPG 2006 I 53 and BSPG 2011 I 116, the articular facets are relatively broader, and their posterior margin is straight. Exceptions are CMN 41856, a probably 9th cervical, in which the prezygapophyses are broad.

All prezygapophyseal facets are dorsomedially exposed. The angle between the prezygapophyseal facets is approximately 115°–130°. The articular facets are very slightly flexed anteroposteriorly, and somewhat tilted posteriorly, obviously reflecting a notable upward bent of the neck at its base. Therefore, the posterior connections of the prezygapophyses to the table of the transverse processes are very short. There is no epipophyseal prezygapophyseal lamina (eprl). The prdl start anteriorly at the apex of the prezygapophyses, and run ventrolaterally towards the diapophyses. The prezygapophyseal pedicles display a large anteromedial surface, which is not excavated by prezygodiapophyseal fossae. The prezygapophyses lack interprezygapophyseal laminae, which McFeeters et al. (2013) considered an autapomorphic feature of *Sigilmassasaurus*, but which is also the case in *Ichthyovenator* (BK10-17–BK10-25).

The postzygapophyses have a spoon-like shape, are slightly less widely spaced than the prezygapophyses, and project posteriorly beyond the level of the posterior articular facet, overhanging the latter for approximately half the length of their articular facets. They have a slightly concave facet with a posteroventral and lateral orientation. The postzygapophyses are anteromedially connected to the neural spine by robust spinopostzygapophyseal laminae. These laminae border a dorsoventrally high, trough-like spinopostzygapophyseal fossa laterally. The fossa is ventrally open, since an interpostzygapophyseal lamina is missing. However, the medial sides of the postzygapophyseal pedicles show thin, flange-like laminae that project into the interpostzygapophyseal space. These flanges are lost or at least partially broken in most specimens, although their bases are usually recognizable. BSPG 2011 I 115 is the only specimen that preserves what seems to be an entirely intact right flange. The flange is triangular, with straight medial and posterior margins that meet at an angle of 90°. As mid-cervical vertebrae seem to close the flanges to a continuous interpostzygapophyseal lamina (NHMUK PV R 16427 has closed flanges with a suture, CMN 50791 has a continuous interpostzygapophyseal lamina), this feature seems to be of positional relevance.

None of the posterior cervicals bear epipophyses. However, the penultimate elements BSPG 2011 I 115 and CMN 41774 have a marked kink in the course of their spinopostzygapophyseal laminae. In dorsal view, the angle of divergence of the postzygapophyses notably increases posterior to the kink. Furthermore, the bone surface at the kink is slightly rugose, indicating the attachment of muscles or tendons. Therefore, the kinks might be the remnant of the epipophyses found in the mid-cervicals. In BSPG 2011 I 116, there is a notable depression on either side of the base of the neural spine, which has a rugose texture and might have served for as a ligament attachment site. This depression reaches anteriorly to approximately two thirds of the anteroposterior length of the base of the neural spine; its anterior border is especially well-defined.

The neural spine is damaged or completely broken in all specimens. The preserved bases or partial spines show that the neural spine was an anteroposteriorly short, spiky process. The base of the spine is anteroposteriorly especially short, being approximately as long anteroposteriorly as wide transversely, in the more anterior posterior cervicals, such as BSPG 2006 I 56 and BSPG 2011 I 115. In the former, the spine seems to have been rather straight, whereas the base indicates a very slight posterodorsal inclination in the latter. In the last cervical BSPG 2011 I 116 the base of the neural spine is slightly longer anteroposteriorly, being 1.5 to two times as long as wide.

Two spinoprezygapophyseal laminae (sprl) connect the neural spine to the medial aspect of the prezygapophyses. The laminae are generally poorly developed, and mainly marked as the edge separating the anterior from the lateral surface of the neural spine. The edges are least marked in the probable C9 (e.g., BSPG 2011 I 115). Because the prezygapophyses are situated far laterally in respect to the neural spine, the spinoprezygapophyseal laminae diverge from the apex ventrally and form a reverted “V.” The resulting anteriorly exposed, triangular area is medially parted by a prespinal lamina (prsl). The prespinal lamina is a low, but stout ridge that extends probably from the apex of the neural spine to its base above the opening of the neural canal. In some specimens, for example BSPG 2011 I 116, the prsl projects slightly ventrally beyond the dorsal margin of the neural canal and forms an overhanging tip, which results in a heart-shaped outline of the neural canal opening. To either side of the prsl, a shallow, longitudinal depression is present in the last cervical vertebrae.

### Dorsal vertebrae

The first dorsal vertebra of *Sigilmassasaurus*, represented by a total of four vertebrae in our sample (BSPG 2006 I 54, BSPG 2006 I 55, CMN 41857, NHMUK PV R 16434; Figs. 9–11), including the holotype of *S. brevicollis*, is notably similar to the posterior cervical vertebrae, up to a parapophysis that is placed on the anteroventral end of the centrum. This is in contrast to the vast majority of theropods, in which this process shows a marked dorsal shift in the first dorsal. Indeed, but for the comparison with the complete cervical column and first dorsal vertebra of *Ichthyovenator* (BK10-25), one could take these vertebrae for posterior cervicals.

More posteriorly placed dorsal vertebrae of *Sigilmassasaurus* can be identified on the basis of progressively elevated parapophyseal positions, a regressed expression of the hypapophyses, sharp but low ventral keels, absence of lateral pneumatic foramina of the centrum in all but he most anterior elements, horizontal to slightly dorsally elevated and slightly posteriorly directed transverse processes, elongation of transverse processes, more elaborate transverse process lamination, anteroposteriorly elongate bases of the neural spine, and progressively more narrowly spaced pre- and postzygpophyses.

**Figure 12:**
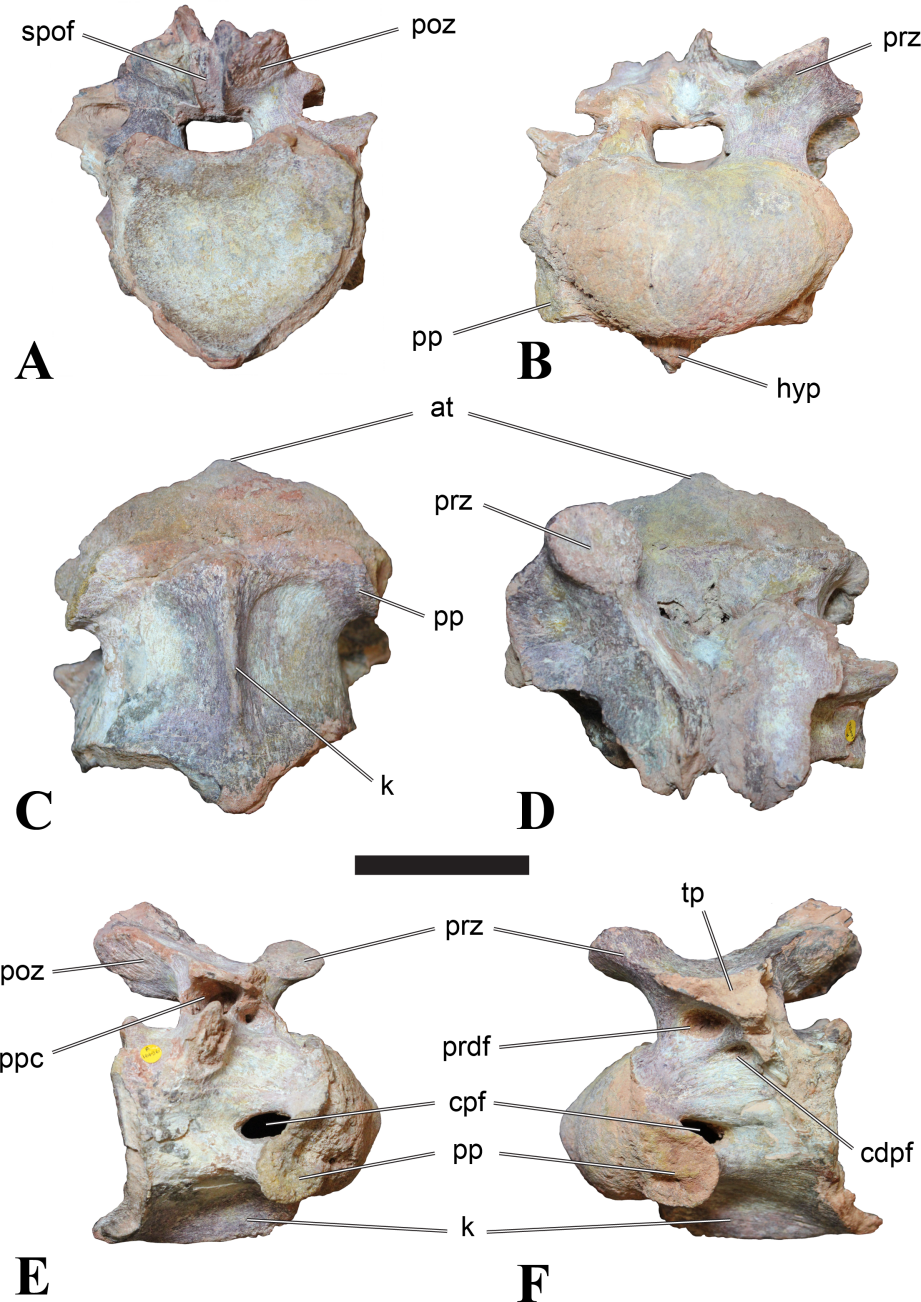
NHMUK PV R 16436, anterior dorsal vertebra (D2) of *Sigilmassasaurus brevicollis*. (A) posterior view; (B) anterior view; (C) ventral view; (D) dorsal view; (E) right lateral view; (F) left lateral view. Abbreviations: at, anterior medial tuberosity; cdpf, centrodiapophyseal fossa; cpf, central pneumatic foramen; hyp, hypapophysis; k, keel; poz, postzygapophysis; pp, parapophysis; ppc, posterior pneumatic chamber of the postzygodiapophyseal fossa; prdf, prezygodiapophyseal fossa; prz, prezygapophysis; spof, spinopostzygapophyseal fossa; tp, transverse process. Scale bar equals 5 cm.

**Figure 13:**
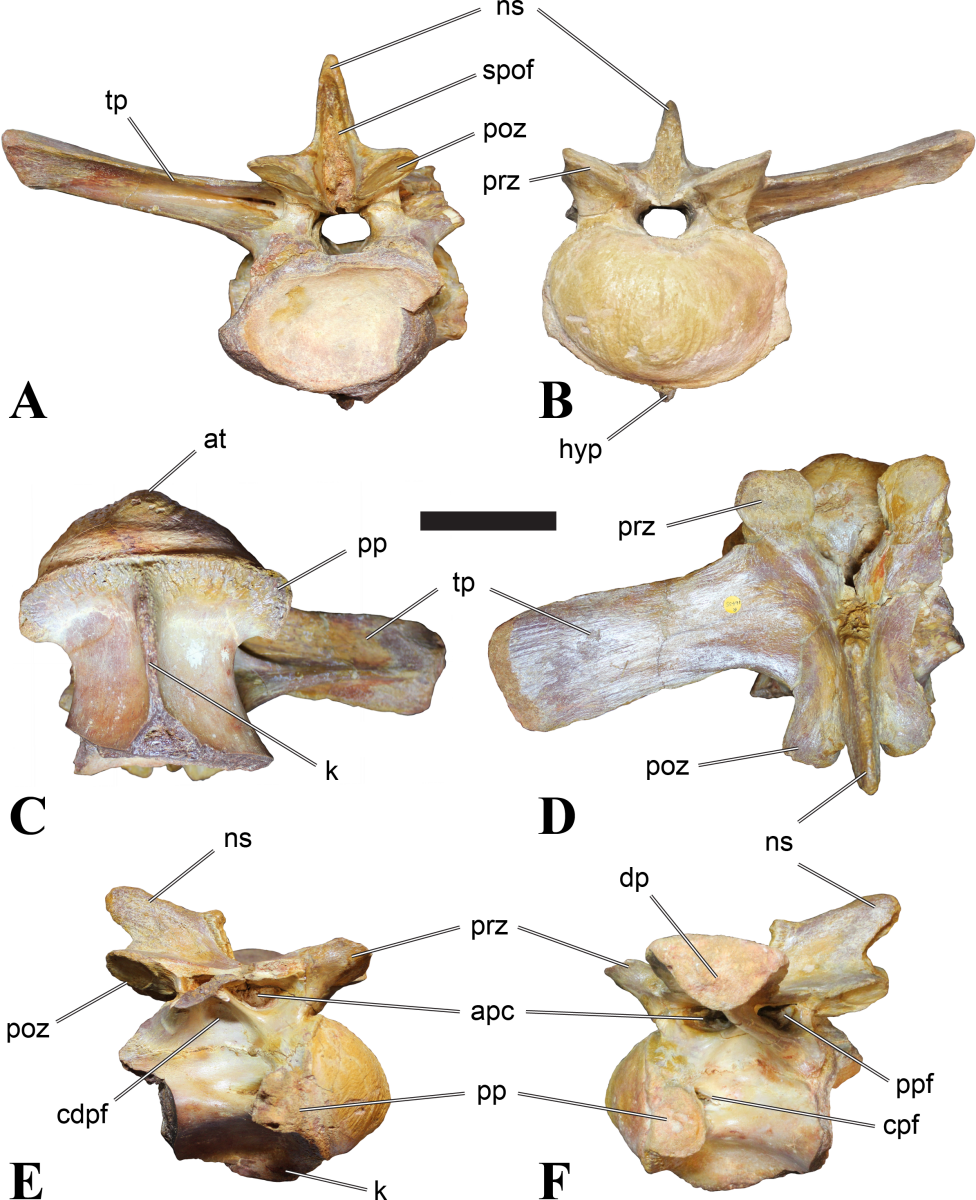
NHMUK PV R 16435, anterior dorsal vertebra (D3) of *Sigilmassasaurus brevicollis*. (A) posterior view; (B) anterior view; (C) ventral view; (D) dorsal view; (E) right lateral view; (F) left lateral view. Abbreviations: apf, anterior pneumatic foramen of the prezygodiapophyseal fossa; at, anterior medial tuberosity; cdpf, centrodiapophyseal fossa; cpf, central pneumatic foramen; dp, diapophysis; k, keel; ns, neural spine; poz, postzygapophysis; pp, parapophysis; ppf, posterior pneumatic foramen of the postzygodiapophyseal fossa; prz, prezygapophysis; spof, spinopostzygapophyseal fossa; tp, transverse process. Scale bar equals 5 cm.

**Figure 14:**
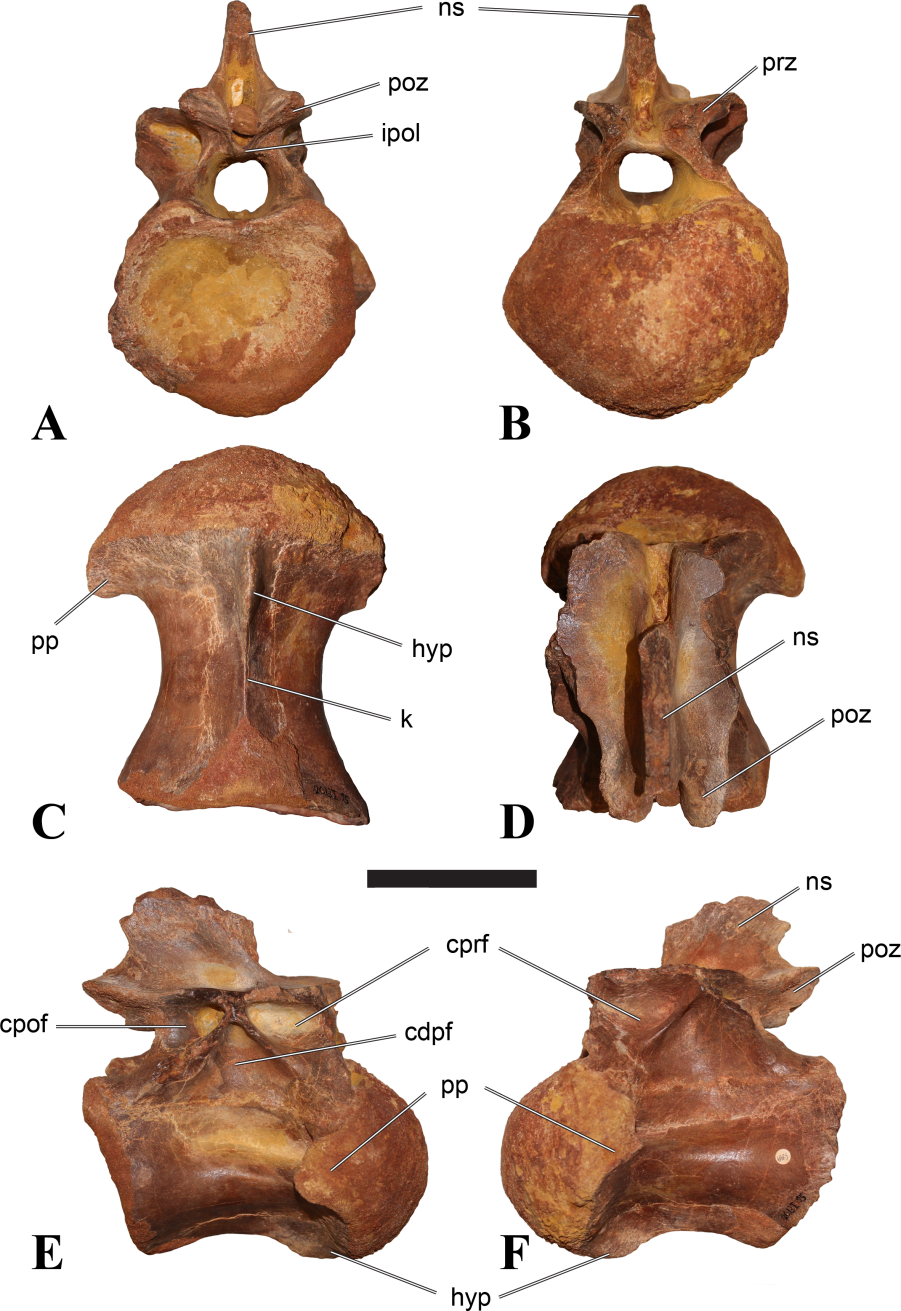
BSPG 2013 I 95, anterior dorsal vertebra tentatively referred to *Sigilmassasaurus*. (A) posterior view; (B) anterior view; (C) ventral view; (D) dorsal view; (E) right lateral view; (F) left lateral view. Abbreviations: cdpf, centrodiapophyseal fossa; cpof, centropostzygapophyseal fossa; cprf, centroprezygapophyseal fossa; hyp, hypapophysis; ipol, interpostzygapophyseal lamina; k, keel; ns, neural spine; poz, postzygapophysis; pp, parapophysis; prz, prezygapophysis. Scale bar equals 5 cm.

We thus identify CMN 41858, NHMUK PV R 16435 and NHMUK PV R 16436 (Figs. 12 and 13; note that the holotype vertebra is adequately figured in McFeeters et al., 2013) as dorsal vertebrae posterior to D1, and BSPG 2013 I 95 (Fig. 14) and CMN 41850 as anterior dorsal vertebrae possibly belonging to *Sigilmassasaurus* (see below). While their dorsal positioning in the axial series can be determined following the above trends, several features support their referral to *Sigilmassasaurus*. NHMUK PV R 16436, NHMUK PV R 16435 especially, and the poorly preserved CMN 41858 closely resemble the posterior cervical and first dorsal vertebrae of *Sigilmassaurus*, so that there can be little doubt that they represent the same taxon. The latter specimen resembles the first dorsal vertebra slightly more in the morphology of its transverse process and neural spine and NHMUK PV R 16436 is very similar to this vertebra in all comparable characters, so that these elements might represent the second dorsal, whereas NHMUK PV R 16435 might be a third dorsal. BSPG 2013 I 95 and CMN 41850 are very similar to each other in their morphology, but differ markedly from the posterior cervicals or anteriormost dorsals of *Sigilmassasaurus*. These vertebrae lack autapomorphies of *Sigilmassasaurus*, which are all based on more anterior vertebral material. However, a tentative referral of these specimens to *Sigilmassasaurus* is supported by a suite of features shared between BSPG 2013 I 95 and CMN 41850 with *Sigilmassasaurus*, some of which are unusual for theropods but not exclusively present in *Sigilmassasaurus*. These include their notably wide and opisthocoelous anterior articular surface (while *Allosaurus* [SMA 0005, MOR 693], for instance, has greatly reduced anterior condyles in the anterior dorsal vertebrae and loses the opisthocoelous state completetly by the third dorsal vertebra,) the presence of a marked, concave ventral keel with a small anterior hypapophysis, and the presence of a subtle median tuberosity on the anterior condyle in BSPG 2013 I 95. Also, the neural spines of both vertebrae are not expanded at their base, as in “*Spinosaurus* B” but unlike in *Spinosaurus aegyptiacus* or *Ichthyovenator* (Stromer, 1931; Allain et al., 2012). The two vertebrae can be clearly assigned to an anterior dorsal position within the axial series of a large theropod, as they show several features indicative of such a position (see below). Notably, many features of their morphology fit well with expectations of anterior dorsal vertebrae of *Sigilmassasaurus* following vertebrae such as NHMUK PV R 16435. For example, the parapophyses of BSPG 2013 I 95 and CMN 41850 are slightly more dorsally positioned than in NHMUK PV R 16435, or the central pneumaticity is further reduced to a fossaeous depression on the lateral side of the centrum (see below). We therefore identify these vertebrae as more posterior anterior dorsal vertebrae likely pertaining to *Sigilmassasaurus*, with their difference from the anteriormost dorsals being due to differences in position within the vertebral column, and include them in this description for comparative reasons. We acknowledge that this referral is tentative at this stage, but feel that the differences to other spinosaurid dorsal vertebrae such as *Spinosaurus* (Stromer, 1915) and the features indicative of spinosaurid affinities and compatible with *Sigilmassasaurus* justify this referral, pending associated discoveries.

Parts of the right postzygapophysis and the distal part of the right transverse process is missing in BSPG 2006 I 54. CMN 41857 has a fairly complete neural arch, with parts of the right transverse process and the tip of the spine missing. BSPG 2006 I 55 and NHMUK PV R 16434 are fairly complete specimens. Both lack parts of their right transverse processes, BSPG 2006 I 55 lacks the distal part of its left postzygapophysis, neural spine and right parapophysis, and NHMUK PV R 16434 has a broken right postzygapophysis and partly eroded rim of the posterior articular facet. NHMUK PV R 16436 lacks the right prezygapophysis and postzygapophysis, the distal part of both transverse processes, and has a strongly eroded posterior articular facet. NHMUK PV R 16435 is one of the most complete dorsal vertebrae described, only missing its right transverse process. In CMN 41858, most of the transverse processes, the tip of the neural spine, and the anterior part of the centrum is broken off. BSPG 2013 I 95 is a large specimen that lacks most of its transverse processes, both prezygapophyses, and the dorsal part of the neural spine. CMN 41850 lacks the pre- and postzygapophyses and the distal parts of the transverse processes are broken.

The centrum of the first dorsal vertebra corresponds closely to those of the posteriormost cervicals in most characters. The anterior articular surface is strongly opisthocoelous and very wide, being more than 1.7 times wider than high and wider than the centrum is long. A strongly developed median tuberosity is present on the anterior condyle. The posterior articular surface is higher than the anterior surface, but the dorsal rim of both surfaces is approximately level. However, the anterior articular surface is slightly angled dorsally so that the centrum is slightly shorter dorsally than ventrally. Thus, the neck curved upwards from the base of the dorsal vertebral column. The posterior articular surface is deeply concave and reniform in outline. A very deep ventral keel is present and becomes deeper anteriorly, where it ends in a prominent hypapophysis just beneath a transverse ridge connecting the parapophyses. The hypapophysis is transversely expanded in comparison to the ventral side of the keel and overhangs the latter anteriorly with its tip, being offset from the ventral end of the anterior articular surface by a well-developed, anteriorly facing transverse groove. In anterior view, the hypapophysis displays a transversely broad base. The keel is slightly convex along its course in lateral outline, and the edge tends to be thicker than its sheet. Both the relative depth and the ventral convexity of the ventral keel seem to increase during ontogeny: whereas this structure is still rather low and almost straight ventrally in the smallest specimen (BSPG 2006 I 55), it is especially deep and strongly convex in large specimens, such as BSPG 2006 I 54 and NHMUK PV R 16434. Posteriorly, the keel merges into the rim of the posterior articular facet, thereby broadening into a small triangular platform. The surface of this platform is faintly striated by longitudinal rugosities. The rugosities are also apparent on the holotype of *Sigilmassasaurus* (CMN 41857), although previous authors have not mentioned this feature (Russell, 1996; McFeeters et al., 2013). As in the posterior cervicaly, the area between the keel and the marked lateroventral edges is depressed into a very shallow fossa. In BSPG 2006 I 54, there are two small foramina visible on the anterior rim of the fossa in posterior view. They are positioned at the base of the hypapophysis, somewhat medially on the posterior side of the transverse ridge connecting the parapophyses. In the right fossa, there are two small foramina, both of approximate circular shape. On the left side, the rim of the fossa is penetrated by only one foramen. This left foramen is bigger and more oval in outline than those on the other side, and it may be speculated that this foramen results from the confluence of the two on the right side. BSPG 2006 I 54 is the only specimen in which those foramina could be observed. These foramina do not seem to lead into an internal chamber, and are thus apneumatic (see ‘CT’ below). Instead, the foramina might be associated with the vascular system of the animal.

The anterior ventral ridge connecting the parapophyses transversely is somewhat expanded ventrally, resulting in an almost straight ventral margin between these processes in anterior view. Nevertheless, the articular surfaces of the parapophyses are still ventrolaterally directed, as in the posterior cervical vertebrae, though less strongly than in the latter elements. The articular surface of the parapophysis is oval in utline, being longer than high. It is large, accounting for one third to almost one half of the length of the centrum (excluding the anterior convexity).

Large pneumatic foramina are present in the first dorsal vertebra on the lateral side, above the parapophysis. The foramina are oval in outline, being longer than high. The smallest specimen, BSPG 2006 I 55, is unusual in that the foramen cuts a deep transverse trough in the dorsal margin of the parapophysis, resulting in a triangular outline of this opening.

A lateral bulge and associated dorsal fossa is well-developed in the first dorsal vertebra. In CMN 41857 (holotype of *S. brevicollis*), the lateral bulge crosses the neurocentral suture and is therefore slightly different from the one described above for BSPG 2006 I 53. However, in other first dorsals, including BSPG 2006 I 54, BSPG 2006 I 55, and NHMUK PV R 16434 the lateral bulge is similar to the one of BSPG 2006 I 53.

As in the posterior cervicals, the neurocentral sutures are clearly visible in all first dorsals. Interestingly, the smallest specimen, BSPG 2006 I 55, shows rather tight suturing of the neural arch to the centrum, despite being only almost half the size of the largest vertebra. Thus, it seems unlikely that the degree of suture fusion is a reliable indicator of skeletal maturity in *Sigilmassasaurus*.

As in the cervical vertebrae, the neural arch pedicles meet in the midline below the neural canal in the anterior half of the centrum. In large specimens, the entire anterior floor of the neural canal is thus formed by the medial expansions of the pedicles. In contrast, in the smallest specimen, BSPG 2006 I 55, the medial contact between the pedicles is small, indicating that this suture expands during ontogeny. The neural canal is large and slightly wider dorsally than ventrally, as in the cervical vertebrae, including the type of *Spinosaurus maroccanus*. In several vertebrae, the dorsal margin of the canal is slightly expanded ventrally in its central part, resulting in a heart-shaped outline of the canal. There seems to be a negative allometry in the size of the neural canal, as the smallest specimen BSPG 2006 I 55 has the relatively largest opening.

The neural arch, transverse processes and zygapophyses of the first dorsal vertebra are very similar to those of the last cervical. The transverse process is very slightly ventrolaterally directed, at an angle of approximately 15–20° from the horizontal in BSPG 2006 I 54, BSPG 2006 I 55, NHMUK PV R 16434, and CMN 41857. In contrast to the posterior cervicals, the distal end of the processes is not or only very slightly flexed ventrally, but the diapophysis remais ventrolaterally directed. In dorsal view, the processes expand slightly distally. The anterior margin is almost straight to slightly concave, so that the moderate distal expansion in these elements mainly stems from a funnel-like divergence of the anterior and posterior margins. The dorsal surface of the process twists slightly towards the distal end, so that it faces slightly anterodorsally. Lateral lamination of the neural arch is very similar to that seen in posterior cervicals. Only in BSPG 2006 I 54, the ridge ventral to the transverse process branches into rudimentary posteriorly and anteriorly oriented laminae in its proximal part. These are the posterior centrodiapophyseal laminae (pcdl) and anterior centrodiapophyseal laminae (acdl), respectively. The pcdl is disproportionally stronger developed than the acpl. It is also longer and extends to a more ventral point on the lateral side of the vertebra than the acpl. The bifurcation of the centrodiapophyseal lamina into acdl and pcdl serves as the dorsal roof of a fossa on the ventrolateral part of the neural arch, which is confluent with the depression above the dorsal bulge dorsal to the pneumatic foramen on the anteroventral part of the centrum described above. However, both the distinction of the laminae and the fossa are much less developed than in *Baryonyx* (NHMUK PV R 9951) and *Ichthyovenator* (BK10-25).

As in the posterior cervical vertebrae, stout prezygodiapophyseal and postzygodiapophyseal laminae are present and extend far laterally onto the anterior and posterior edge, respectively, of the transverse process. In contrast to the former, however, the prezygadiapophyseal lamina rapidly becomes more robust and flexes ventrally distally, thus contributing to the distal twist of the transverse process. Furthermore, the postzygapophysis is somewhat more offset from the transverse process than in posterior cervicals, so that the ridge representing the postzygodiapophyseal lamina is lower and slightly more dorsolaterally directed.

Large pneumatic foramina leading into the base of the transverse process are present on the anterior and posterior side, very similar to the situation in the posterior cervicals. These foramina can be developed quite asymmetrically, as for example in BSPG 2006 I 54, in which the right foramen is much larger than the one on the left side (Fig. 9). The pneumatic organization of the transverse processes in BSPG 2006 I 55 is similarly asymmetric (Fig. 10). While the left posterior pneumatic foramen of the transverse process is developed as a regular, transversely elongate opening, the right foramen is developed as a very small, round opening which leads into a channel-like diverticulum. Simultaneously, the anterior pneumatic opening on the right transverse process is unusually large. On both transverse processes, the anterior and posterior pneumatic cavities are not interconnected. In this specimen, the left postzygapophysis is broken, and the exposed base is hollow. This internal cavity does not seem to be connected with the pneumatic foramina in the transverse process, and it might have served as a chamber for fatty tissue or marrow, but a taphonomic origin of the feature can also not be ruled out completely.

The prezygapophyses of D1 do not overhang the anterior end of the centrum and their posterior half is placed over the base of the transverse process. They stand on rather high, dorsally projecting stalks and are connected to the anterior margin of the transverse process by a stout lamina that extends laterally to approximately half the length of the process. The articular surface of the prezygapophysis is anteroposteriorly expanded and overhangs the stalk anteriorly and posteriorly. The articular surfaces stand at an angle of 100°–110° to each other and are elongate oval in outline. However, there seems to be some variation in this character, as the holotype of *S. brevicollis* (CMN 41857) has differently shaped left and right prezygapophyses (see McFeeters et al., 2013).

The postzygapophysis projects posterodorsally and overhangs the centrum posteriorly for one third to half of its length. The articular surface is elongate oval in outline, placed approximately at the same level as the prezygapophysis and faces ventrolaterally and slightly posteriorly. As in the posterior cervicals there is neither an interpostzygapophyseal lamina, nor an epipophysis.

As in the ultimate cervical BSPG 2011 I 116, there is a well-developed, but more shallow and less rugose longitudinal depression on either side of the neural spine in BSPG 2006 I 54, BSPG 2006 I 55, and CMN 41857. In these specimens, the depression is less marked and reaches anteriorly to the anterior end of the spine. Such a depression flanking the neural spine in the ultimate cervicals has not been described in any other theropod and might thus represent an autapomorphy of *Sigilmassasaurus*, although there seems to be some variation in the development of this feature.

The only specimen that has a complete spine preserved, NHMUK PV R 16434, shows that this structure was anteroposteriorly short and low, being approximately as high as the neural arch between the centrum and the prezygapophyses (Fig. 11). In this element, the spine is anteroposteriorly elongate at its base, but continuously tapers dorsally. It is also slightly inclined posteriorly, being curved, with a notably convex anterior and a slightly concave posterior margin. A stout prespinal lamina is present in the apical part of the neural spine, but becomes less marked ventrally. The development of spinopre- and spinopostzygapophyseal laminae corresponds to the situation in the last cervical (BSPG 2011 I 116).

In the more posterior anterior dorsal vertebrae, intercentral articulations are also broader than high, with width–height ratios ranging from approximately 1.7 in NHMUK PV R 16436 to c. 1.3 in BSPG 2013 I 95 and CMN 41850. The vertebrae are strongly opisthocoelous, with the more posterior elements being only insignificantly less convex anteriorly. A medial tuberosity is very prominent in NHMUK PV R 16436 (Fig. 12), clearly present in NHMUK PV R 16435 and developed as a very subtle bump in BSPG 2013 I 95, but could not be identified in CMN 41850. As in other *Sigilmassasaurus* specimens, both the anterior and the posterior articular facets have a reniform outline. The posterior articular surface is deeply concave in all specimens, indicating that even the dorsal vertebrae following BSPG 2013 I 95 and CMN 41850 in position had convex anterior articulations. Anterior and posterior articulations do not show an offset to one another in NHMUK PV R 16436 and NHMUK PV R 16435, but in BSPG 2013 I 95 and CMN 41850, the anterior articular end is slightly displaced ventrally in relation to the posterior face, indicating that the anterior part of the dorsal vertebral column was flexed anteroventrally. A marked rim around the anterior articular facet, as it is also present in the cervical vertebrae, is present in NHMUK PV R 16436 and NHMUK PV R 16435, but only poorly marked in the more posterior elements.

BSPG 2013 I 95 and CMN 41850 both have a low but sharp keel, which follows the constricted outline of the centrum in lateral view, and is accordingly concave ventrally in lateral view. Anteriorly, the keel develops into a small, rounded and slightly thickened hypapophysis, which projects slightly further ventrally than the keel itself. Posteriorly, the keel becomes less conspicuous and merges into a low triangular expansion at the posterior end of the centrum.

CMN 41858 does not preserve a keel, as the ventral and anterior parts of the centrum are broken off, but a strongly developed ventral keel is present in NHMUK PV R 16436. The keel in the latter vertebra and NHMUK PV R 16435 differs from those in the more posterior dorsal vertebrae in that it is more notably transversely broadened anteriorly and strongly convex ventrally in lateral view, but a strongly developed hypapophysis, as it is present in D1, is absent. Although the posterior end of the keel is eroded in both specimens, the preserved parts in NHMUK PV R 16435 indicate that it terminated in a posteriorly broadening triangular expansion towards the rim of the posterior central articulation facet, as in the cervicals and more posterior dorsals.

The position of NHMUK PV R 16436 and NHMUK PV R 16435 as among the anteriormost dorsals is supported by the parapophyseal position. The parapophyses in the former specimen are still very low on the centrum and only very slightly elevated over its ventral rim. In NHMUK PV R 16435, they are slightly higher positioned on the centrum, so that the ventralmost part of the rim of the anterior articular facet can be seen below them in lateral view (Fig. 13). However, the parapophyses remain on the ventral half of the centrum in this specimen. They project only slightly lateral from the lateral rim of the anterior articular facet and are large, being only slightly lower than half the height of the centrum. The articular surface of the parapophysis is oval in outline, being higher than long and becoming wider ventrally, and is laterally directed. The parapophyses attain a substantially higer position in BSPG 2013 I 95 and CMN 41850, in which they are situated at or just above the mid-height of the centrum, below the anterior part of the neurocentral suture. In these specimens, the parapophyses barely project laterally from the rim of the anterior articular end of the centrum, and are confluent anteriorly with the anterior condyle. The parapophyses in these specimens are relatively smaller than in NHMUK PV R 16436 and NHMUK PV R 16435. Their articular surface is rounded triangular in outline, being wider ventrally. In CMN 41858, the parapophyses are not preserved. In all dorsals a stout ridge connects the posterior margin of the parapophysis with the lateral side of the centrum. From this ridge, a rounded edge extends posteriorly, and partitions the side of the centrum into a dorsal, mainly laterally directed surface, and a ventrolateral surface that extends to the keel. The edge is marked and the surface ventral to it is flat and faces mainly ventrally in NHMUK PV R 16436 and NHMUK PV R 16435, so that the ventral keel rises abruptly from the ventral side, as in the posterior cervical vertebrae. In the more posterior dorsals, the edge is less notable, the ventral side is ventrolaterally directed, and the ventral keel arises gradually from it.

The lateral pneumatic foramina are large in NHMUK PV R 16346 and NHMUK PV R 16435. In the former, they are situated posterodorsal to the parapophyses and well visible in lateral view, as in the posterior cervicals and first dorsal. In NHMUK PV R 16435, they are situated posterior to the parapophyses, so that they invade the centrum in anteromedial direction and are barely visible in straight lateral view. The left foramen is triangular in outline, while the right opening is oval. Dorsal to the pneumatic foramen, a shallow, but large fossa stretches over the neurocentral suture and is dorsally bordered by the centrodiapophyseal laminae of the transverse process (see below). Pneumatic foramina are absent in BSPG 2013 I 95 and CMN 41850, but a large lateral depression is present on the centra of these vertebrae (Fig. 14). This depression is anteroposteriorly elongate and extends from posterodorsal of the parapophysis over approximately two thirds of the length of the centrum. It is bordered ventrally by the edge extending posteriorly from the posterior end of the parapophysis, which becomes dorsoventrally wider posteriorly, and shallows dorsally towards the neurocentral suture, which forms its dorsal margin. Anteriorly, it undercuts the dorsal part of the parapophysis and the anterior margin of the centrum to various degrees, but it is never associated with a pneumatic foramen. Nothing can be said about the situation in CMN 41858, which does not preserve the part of the centrum in which a pneumatic foramen would be present.

The neurocentral suture extends far ventrally, to almost the half height of the centrum in NHMUK PV R 16436 and NHMUK PV R 16435 (and, apparently, CMN 41858), as in the posterior cervical and first dorsal vertebrae. Unlike in the latter, the pedicles do not meet in the midline at the floor of the neural canal in NHMUK PV R 16435, although they are only seprated by a very narrow strip of bone of the centrum. In the more posterior dorsals, the neurocentral suture is placed higher on the centrum, less than one-fourth of the height of the latter from its dorsal margin. The neural canal floor of BSPG 2013 I 95 is developed as a deep trench, which is bordered by a lateral elevation of the centrum on either side, on which the neural arch pedicles articulate. This part of the opening is largely hidden in anterior view by the dorsal part of the rim surrounding the anterior articular facet. The neural canal itself is circular, as in other specimens, but the ventral trench-like extension contributes to an overall keyhole-shape of the neural canal. The neural canal is circular in CMN 41850, CMN 41858 and NHMUK PV R 16435, and the neural canal floor is slightly depressed and does not show basivertebral foramina, although this cannot be determined in CMN 41858 with certainty, as the neural canal floor suffered from erosion. In NHMUK PV R 16436, the neural canal has a rectangular outline and is wider than high.

The neural arches in these dorsal vertebrae of *Sigilmassasaurus* are characterized by an increase in lateral lamination in comparison to the cervical and first dorsal vertebrae. Furthermore, the only completely preserved transverse process in NHMUK PV R 16435 is strongly elongate, and the neural spines are more ‘typical’ for tetanuran theropods than the spiky processes seen in cervical and first dorsal vertebrae.

Transverse processes are slightly dorsally inclined in these dorsal vertebrae of *Sigilmassasaurus*. While the broken process bases of NHMUK PV R 16436, BSPG 2013 I 95 and CMN 41850 do not allow a precise quantification of the dorsal elevation, the transverse processes are projecting with 15–25° off the horizontal in CMN 41858 and NHMUK PV R 16435. The only specimen that completely preserves one transverse process, however, is NHMUK PV R 16435 (Fig. 13). In this specimen, the length of the complete left transverse process equals 117% of the central width of the specimen. In this specimen, the transverse processes are situated directly behind the prezygapophyses, and are slightly posteriorly inclined in dorsal view. The latter is also the case in CMN 41858.

All of these dorsal vertebrae have a consistent arrangement of laminae: The prezygodiapophyseal and postzygodiapophyseal laminae are sharply edged laminae spanning the planar dorsal table of the transverse processes and connect the latter with the pre- and postzygapophysis, respectively. Because the pre- and postzygapophyses are situated only slightly above the level of the transverse processes from D3 onwards, the prdl and podl do not curve upwards when approaching the medial base of the transverse processes, in contrast to the situation in the cervical and first dorsal vertebrae. The probable D2, NHMUK PV R 16436, shows an intermediate condition in that the pre- and postzygapophyses are slightly elevated above the level of the transverse processes (Fig. 12). The transverse processes are braced ventrally by a centrodiapophyseal lamina, which proximally bifurcates into an anterior and posterior centrodiapophyseal lamina. The anterior centrodiapophyseal lamina is shorter than the posterior centrodiapophyseal lamina, which has an enlarged and more robust base on the posterior part of the lateral side of the neural arch.

Anterior and posterior centrodiapophyseal laminae subdivide the area underneath the transverse process into an anterior (prezygo-centrodiapophyseal), a posterior (postzygo-centrodiapophyseal), and a ventral (centrodiapophyseal) fossa, which are all of approximately equal size in BSPG 2013 I 95 and CMN 41850 (Fig. 14). The centrodiapophyseal fossa is small in NHMUK PV R 16346, but becomes progressively larger in more posterior elements. In NHMUK PV R 16435, the posterior fossa is somewhat smaller than the anterior and ventral fossae, because the angle between the posterior centrodiapophyseal and postzygodiapophyseal laminae is more acute than in the more posterior specimens. The anterior centrodiapophyseal lamina meets the posterior centrodiapophyseal lamina anteroventrally before the latter connects to the transverse process in this specimen, while in BSPG 2013 I 95 and CMN 41850 both laminae join in the same point just beneath the dorsal table of the transverse process. In NHMUK PV R 16435, the only specimen which preserves an entire transverse process, the podl curves laterally from its posterior buttress to the underside of the transverse process, and continues as a stout ridge almost to the distal end of the process. The ridge is placed slightly posterior to the mid-width of the process and becomes gradually lower distally. The anterior centrodiapophyseal lamina joins the stronger posterior centrodiapophyseal lamina in the medial-most third of the transverse process. The diapophyseal facet is triangular in outline and strongly ventrally inclined in respect to the long axis of the transverse process. Because of the overall dorsal projection of the transverse process, the net diapophyseal orientation is ventrolateral. In CMN 41858, the separation between anterior centrodiapophyseal and posterior centrodiapophyseal lamina is not as clearly developed as in the other specimens, but similar to the situation in NHMUK PV R 16436. Otherwise, the morphology seems to be similar to that found in NHMUK PV R 16435, but the situation is partly obscured by damage to the specimen.

Although the laminae of the transverse processes define well developed fossae, no pneumatic foraminae leading into internal chambers are evident in CMN 41850 and NHMUK PV R 16435, in contrast to the cervical and first dorsal vertebrae. In BSPG 2013 I 95, the left postzygo-centrodiapophyseal fossa has a pneumatic diverticulum associated with it. The opening extends into a small, finger-like cavity, which penetrates dorsomedially toward the central interior of the neural arch, beneath the neural spine and above the neural canal. A very similar, though smaller recess is also present within the right postzygo-centrodiapophyseal fossa in this specimen. NHMUK PV R 16436 and CMN 41858 also show a posterior pneumatic foramen on the fragmentary bases of the transverse processes, and the morphology of the foramen is reminiscent of posterior cervical and first dorsal vertebrae, in that the subcircular foramen opens anteromedially into the medial base of the transverse process, and is medially bound by a bony wall curving off ventrally from the postzygodiapophysel lamina.

The prezygapophyses are progressively less widely spaced from specimen CMN 41858 and NHMUK PV R 16436, via NHMUK PV R 16435 to BSPG 2013 I 95 and CMN 41850. In the latter two specimens, the prezygapophyses are broken off, but their bases are situated above the neural canal, rather than dorsolateral to it. Because the neural spine does not rise immediately behind the prezygapophysis as in cervical vertebrae, their bases parallel each other above the neural canal, instead of diverging laterally, thus forming the margins of a trench like canal leading toward the neural spine posteriorly. The diverging pattern is retained in CMN 41858 and NHMUK PV R 16436, and, to a lesser extent, in NHMUK PV R 16435. While only the left prezygapophysis is present in NHMUK PV R 16436, the prezygapophyses are completely preserved in the other two specimens. They project approximately as far anteriorly as the anterior condyle, and their articular surfaces are slightly medially inclined and gently flexed. Their outline is spade-shaped to round in CMN 41858 and NHMUK PV R 16436, but oval, being wider than long, in NHMUK PV R 16435. The angle between the prezygapophyses is wider than in the first dorsal vertebra, c. 122° in CMN 41858 and more than 125° in NHMUK PV R 16435. Whereas the base of the prezygapophysis slightly overlaps the anterior part of the transverse process in CMN 41858 and NHMUK PV R 16436, it is placed entirely anterior to the latter in NHMUK PV R 16435. The prezygapophyses are not connected by an interprezygapophyseal lamina in these anterior dorsals, while such a connection is present in BSPG 2013 I 95, in which the space between the bases of the prezygapophyses is ventrally bridged by the anterior part of the neural canal roof. Hypantral articulations are absent in the anteriormost dorsals, but nothing can be said about the situation in BSPG 2013 I 95 and CMN 41850. The centroprezygapophyseal lamina is a thin, dorsomedially oblique and anteriorly directed buttress in BSPG 2013 I 95 and CMN 41850, while anteriorly broad prezygapophyseal pedicles, as they are ‘typical’ for the posterior cervical vertebrae, can be observed in CMN 41858, NHMUK PV R 16436 and NHMUK PV R 16435.

Postzygapophyses are less widely spaced than prezygapophyses in CMN 41858 and NHMUK PV R 16435, and about equally spaced as the prezygapophyses in BSPG 2013 I 95. In CMN 41850, most of the postzygapophyses are broken, but the general similarity to BSPG 2013 I 95 might suggest a similarly narrow spacing. Postzygapophyses are missing in NHMUK PV R 16436. Postzygapophyseal facets are relatively large in CMN 41858 and NHMUK PV R 16435, while they are smaller and more elongate in BSPG 2013 I 95. They are approximately as long as wide in the anteriormost dorsals, but become longer than wide in the more posterior elements. The posterior part of the neural canal is dorsally open in CMN 41858, NHMUK PV R 16436 and NHMUK PV R 16435, as in posterior cervical and the first dorsal vertebrae of *Sigilmassasaurus*, and relatively large and prominent ventromedial flanges of the postzygapophyseal pedicles are evident. In CMN 41850 these flanges meet to form a ventrally convex and dorsally concave interpostzygapophyseal lamina, which closes the neural canal dorsally. In BSPG 2013 I 95, this lamina is expanded ventrally to form an incipient, U-shaped hyposphene. The dorsal concavity of this lamina contributes to a trench-like depression between the postzygapophyses, which forms the ventral part of the spinopostzygapophyseal fossa.

The neural spine of CMN 41858 is similar to that of the first dorsal vertebra of *Sigilmassasaurus*, in that it is posterodorsally inclined, rises a short way behind the prezygapophyses, exhibits a weak triangular area anteriorly, and gradually develops to a rod-like spine, although the apex is missing. The spine is missing in NHMUK PV R 16436. NHMUK PV R 16435 has a completely preserved neural spine, and shows a transitional morphology between the condition known from more anterior vertebrae of *Sigilmassasaurus* and CMN 41850 and BSPG 2013 I 95. The spine is placed on the posterior part of the neural arch and tapers posterodorsally to form a rounded tip. This posterodorsally projecting apex overhangs the space between the postzygapophyses in dorsal view. As in the first dorsal vertebra, the spine is low, approximately as high as the neural arch from the dorsal rim of the centrum to the postzygapophyses. Anteriorly, the neural spine is offset posteriorly from the prezygapophyses and does not show a triangular, ventrally broadening area as it is found in posterior cervical vertebrae and D1. A centrally positioned prespinal lamina, as for example seen in BSPG 2006 I 54, is also absent. Instead, the spine has a straight to slightly concave, mainly anteriorly directed edge. This edge is slightly transversely thickened and rugose in its surface texture and probably served as a relatively strong attachment site for an intervertebral ligament. The rugose area is laterally bordered by rudimentary spinoprezygapophyseal laminae, which become better defined ventrally and diverge anteriorly as low ridges towards the bases of the prezygapophyses. The anterior edge of the spine is lower than its posterodorsal extension and offse from the dorsal margin by a marked kink, posterior to which the dorsal margin is concave, before it then forms a straight, gradually posterodorsally raising edge. The dorsalmost part of the anterior edge has a small, rectangular anterior projection. On the posterior side of the spine, rather slender, but low spinopostzygapophyseal laminae extend from their junction at the apex of the spine ventrally and very slightly laterally towards the medial side of the dorsal surface of the postzygapophysis. The laminae define a narrow spinopostzygapophyseal fossa, which begins a short distance below the apex and becomes deeper ventrally.

In BSPG 2013 I 95 and CMN 41850, the dorsal parts of the neural spines are broken off so that only their bases remain. In their preserved parts, both neural spines show similar features. The neural spine begins relatively far posteriorly to the prezygapophyses. The trench-like canal between the bases of the prezygapophyses leads to an abruptly rising and slightly posterodorsally inclined neural spine. The anterior edge is rugose as in CMN 41850, and its base is laterally bordered by a thin lamina on each side. This lamina becomes lower and disappears rapidly dorsally, leaving a transversely convex anterior edge of the spine. On the posterior side, the neural spines rise slightly anterior to the postzygapophyses, and are connected to the latter by spinopostzygapophyseal lamina, which begin on the dorsal surface of each postzygapophyses. From here, they curve upward to the posterolateral egdes of the spine, hereby defining a small, posteriorly facing groove on the basis of the neural spine. The well-developed laminae are approximately as short as the spinoprezygapophyseal laminae, but persist as low lateral edge of the posterior end of the spine dorsal to the level where the latter lamina disappears. Whereas the anterior edge of the spine is almost vertical and only very slightly inclined posteriorly, the posterior margin shows a more marked posterior inclination in BSPG 2013 I 95. The broken edge of the spine shows that the anterior and posterior margins are slightly thickened in relation to the central part. Due to the breakage, nothing can be said about the height or exact shape of the spine.

### Internal pneumaticity of posterior cervical and anterior dorsal vertebrae

**Figure 15:**
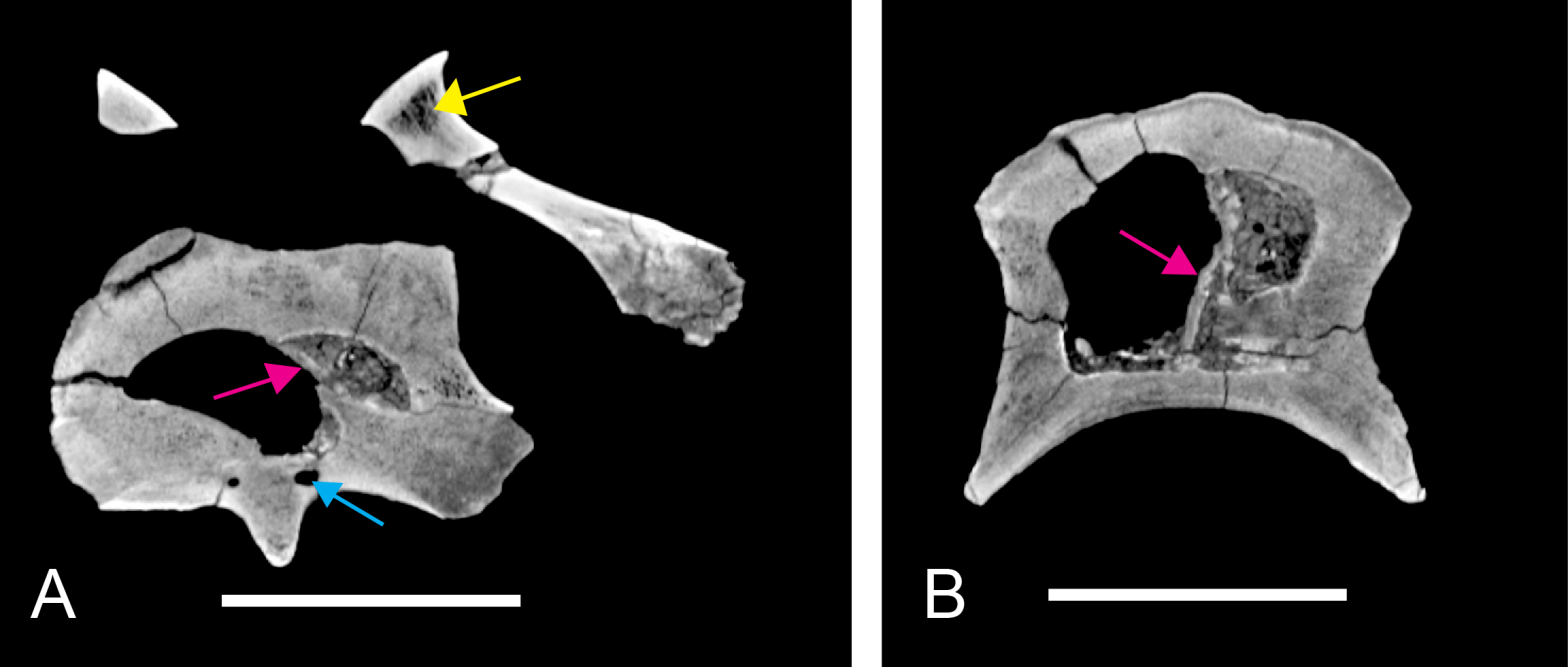
CT images of BSPG 2006 I 54. (A) slice through anterior part of the vertebra in popsterior view. (B) slice through centrum in dorsal view. Pink arrows indicate course of bony septum separating two large camerate chambers. The yellow arrow indicates hollow structure in prezygapophysis. The blue arrow indicates accessory foramen underneath transverse ridge. Scale bars equal 10 cm.

In order to evaluate the internal structure of the posterior cervical and anterior dorsal vertebrae, the specimens BSPG 2011 I 115 (probable C9) and BSPG 2006 I 54 (probable D1) were subjected to CT scanning. The CT slices show several internal structures in BSPG 2006 I 54 (Fig. 15). Most notable, due to their size, are two large chambers in the centrum. The chambers lie adjacent to one another and occupy the left and right part of the interior of the centrum. The right and left chamber are connected to the exterior via the right and left central pneumatic foramina, respectively. Both chambers are separated from one another by a thin bony septum. This septum runs approximately along the midline of the vertebrae, more or less in line with the ventral keel of the centrum. The septum is not perfectly straight over its entire height, which results in an asymmetry of the chambers, the left chamber being bigger than its right counterpart. The right chamber is largely filled with matrix, indicated by a different contrast (in respect to the bone) in the CT images. The chambers fill most of the interior of the centrum. The anterior and posterior walls are about 10 mm thick at the level of the greatest extent of the chambers. The thickness of bone dorsal and ventral to the chamber seems more variable, but the thinnest the ventral surface becomes is about 5 mm. Dorsally, the bone of the centrum retains a minimum thickness of about 15 mm.

The small foramina described for BSPG 2006 I 54, which are located beneath the transverse ridge between the parapophyses and to either side of the hypapophysis, do not connect with interior chambers of the centrum, and are thus most probably apneumatic (Fig. 15).

The foramina on the anterior and posterior side of the transverse processes do not connect internally. The left posterior foramen penetrates deeply into the neural arch pedicle and leads to an internal chamber that is largely filled with matrix.

**Figure 16:**
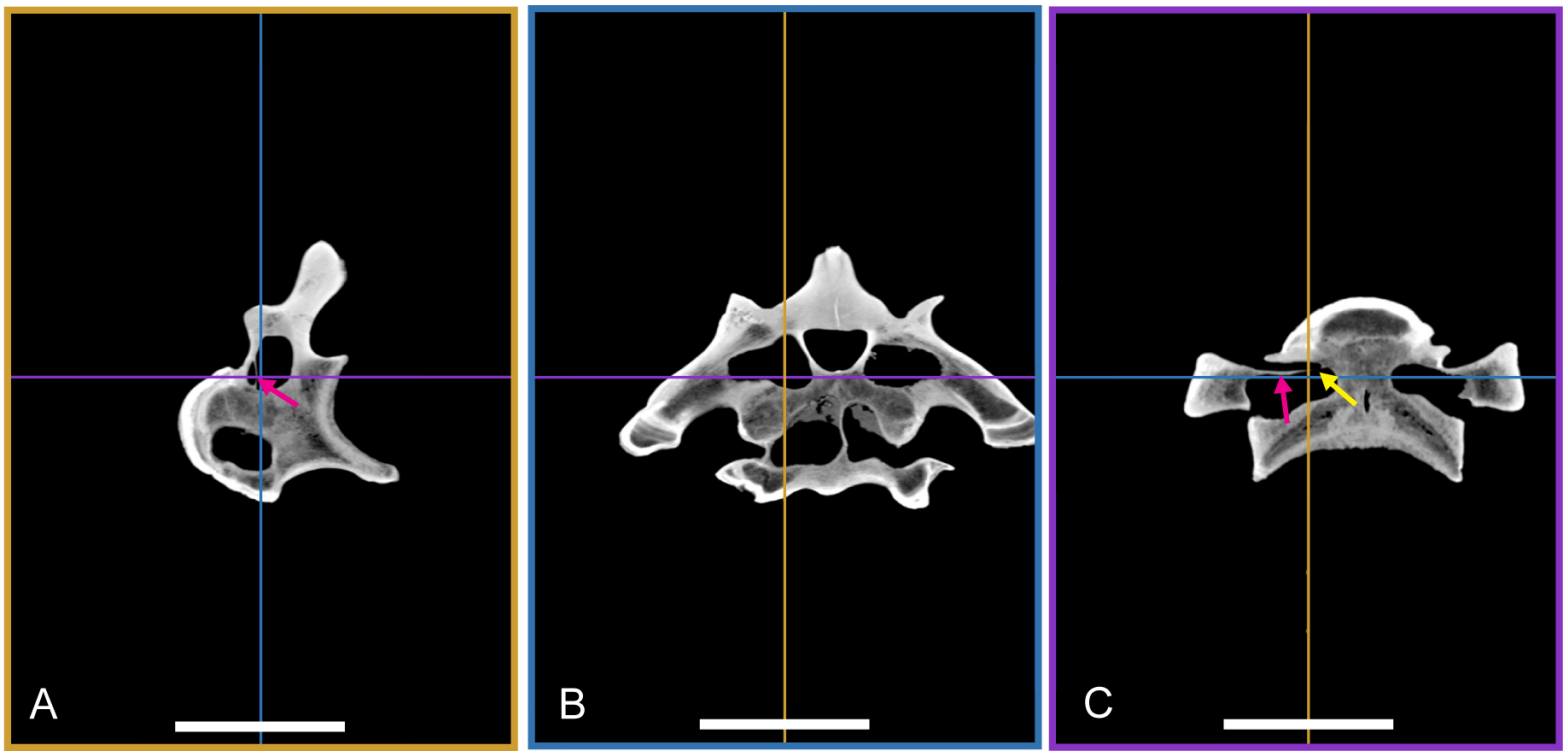
Multi planar reformat (MPR) image of BSPG 2011 I 115. (A) slice through right lateral aspect of vertebra; (B) transversal slice through vertebra in posterior view, (C) horizontal slice through centrum. All sections are orthogonal to one another, with colors indicating relations between the images. The yellow arrow shows connection of posterior and anterior pneumatic cavities of transverse process; The pink arrow shows thin lamina separating cavities laterally. Scale bars equal 10 cm.

The second scanned specimen shows very similar internal structures, as expected. However, there are also some interesting differences to BSPG 2006 I 54. BSPG 2011 I 115 shows the same large internal chambers in its centrum as in BSPG 2006 I 54. Also in BSPG 2006 I 54, those are paired into a left and right chamber and are separated by a bony septum. The chambers are partially filled with matrix, and show different sizes, with the right chamber being larger. One conspicuous difference to BSPG 2006 I 54 is that the various processes of BSPG 2011 I 115 seem to have hollow structures towards their distal ends (Fig. 16). This is particularly well developed in the transverse processes (close to the diapophyses) and in the parapophyses. However, the presence of non-compactly ossified bone areas is also indicated in the pre- and postzygapophyses, and to a weaker extent in the neural spine. A hollow structure was also recognized in the prezygapophysis of BSPG 2006 I 54. The CT slices of BSPG 2011 I 115 also reveal an interior connection of the chambers associated with the anterior and posterior foramina of the transverse processes (Fig. 16), as in the right transverse process of BSPG 2006 I 53. The anterior foramen leads into a tunnel-like cavity, which is laterally separated from the posterior cavity by the bone forming the anterior surface of the transverse process. Only medially in the neural arch pedicle this bone wall is thinning and eventually tapering out to leave a small connection of the cavities.

Together with the observations in other specimens (through pneumatic openings or based on breaks), these results show that vertebral pneumaticity of the cervical and anterior dorsal vertebrae was well developed and penetrated both the vertebral centrum and the neural arch. However, the pneumatic cavities are rather simple and thus correspond to a primitive camerate type (Britt, 1993; Benson et al., 2012a) rather than to the camellate type found in many very large theropods, such as abelisaurids, carcharodontosaurians, and tyrannosaurids (see Benson et al., 2012a). As there seems to be some size-related function in the development of pneumaticity (Benson et al., 2012a), and *Sigilmassausaurus* represents one of the largest theropods known, this might be a significant difference to these taxa. Interestingly, the extremely large Jurassic megalosaurid *Torvosaurus* Galton & Jensen, 1979 is similar to *Sigilmassasaurus* in showing only two large chambers within the cervical vertebrae (Britt, 1991; Britt, 1993).

## Discussion

### Synonymy of *Spinosaurus maroccanus* and *Sigilmassasaurus brevicollis*

In his 1996 paper on the dinosaurs of the Kem Kem area, Russell described two new species of theropods, a new species of *Spinosaurus*, *Spinosaurus maroccanus*, and the new genus and species, *Sigilmassasaurus brevicollis*. As the material was purchased from fossil dealers, and there was thus no information on the possible association of any of these remains, Russell (1996) chose a single vertebra as holotype for each taxon. Thus, Russell (1996) chose a mid-cervical vertebra CMN 50791 as the type of *Sp. maroccanus*, with the ratio of centrum length to centrum height proposed as a diagnostic character distinguishing the two species of *Spinosaurus*. In addition to the holotype mid-cervical vertebra, Russell (1996) referred two additional cervical vertebrae, a dorsal neural arch, and two dentary fragments to *Spinosaurus maroccanus*. Subsequently, Taquet & Russell (1998) referred a partial snout, a fragmentary premaxilla, two cervical centra, and a dorsal neural arch to the same taxon. All of the latter specimens came from the locality of Gara Samani in Algeria, some 550 km to the east of the Kem Kem area. The age of the locality was given as Albian; however, De Broin, Grenot & Vernet (1971) describe the locality as being placed in the “Continental Intercalaire” underlying the Cenomanian transgression, thus in the same stratigraphic position as the ‘Kem Kem beds,’ and it might therefore be of the same age. Since there is no association, nor any overlap between the holotypic vertebra and the referred cranial remains, both from Morocco and from Algeria, and there is evidence for more than one taxon of spinosaurids in at least the Kem Kem, the cranial remains cannot be referred to this taxon with any certainty, and are therefore not further considered here, pending a complete revision of the material.

*Sigilmassasaurus brevicollis* was originally based on a supposed mid-cervical vertebra (CMN 41857; here interpreted as a first dorsal vertebra, see above), to which Russell (1996) referred three further cervical, nine dorsal (mostly represented by isolated centra; McFeeters et al., 2013) and four caudal vertebrae. Additional vertebrae (interpreted as posterior cervicals) were assigned to this taxon by Rauhut (2007) and McFeeters et al. (2013), although this material was not described in detail.

The holotype vertebra of *Spinosaurus maroccanus* differs considerably from that of *Sigilmassasaurus brevicollis*, yet the data presented here shows that these elements share potential synapomorphies and that their differences can be explained by different positions within the cervical vertebral column. This is supported by comparisons with the complete cervical vertebral series of *Ichthyovenator* (Allain, 2014) and *Suchomimus* (S Evers & O Rauhut, pers. obs., 2015), and the partial series of *Baryonyx* (Charig & Milner, 1997), which also show drastic changes between vertebrae depending on vertebral position (see below).

Diagnostic features of the *Sp. maroccanus* holotype include the presence of an elevated, rugose ventral platform in continuation with the weakly developed ventral keel. The ventral platform is clearly visible in lateral view as a pronounced step in the ventral outline. In the type specimen of *Sp. maroccanus*, as well as in BSPG 2011 I 117 and 2011 I 118, this platform is strongly elevated, transversely convex and has a strongly rugose surface. Anteriorly, the platform rapidly lowers into the posteriorly widening ventral keel. In the seventh cervical vertebra of *Ichthyovenator*, as well as in other, probably mid-cervical spinosaur vertebrae from the Kem Kem compound assemblage (BSPG 2013 I 97, ROM 65537), the ventral outline shows a similar, though less marked step, but in ventral view, this step is formed by posteriorly placed lateroventral tubercles with a smooth depressed area in between; a ventral keel is absent. Furthermore, as BSPG 2011 I 117 and BSPG 2011 I 118 most probably do not represent the same vertebral position (as indicated by differences in the development of the ventral keel and platform and the slightly different offset and orientation of the articular surfaces), the stepped ventral outline is present in several cervical vertebrae of *S. maroccanus*, whereas it is only present in C7 in *Ichthyovenator*. Neither a stepped ventral outline, nor a ventral platform is present in any cervical vertebrae of *Baryonyx*, including the probable C7 (identified as C8 by Charig & Milner, 1997), or *Suchomimus* (S Evers & O Rauhut, pers. obs., 2015).

A centroprezygapophyseal fossa, i.e., a depression on the anterior side of the neural arch pedicles defined by the centroprezygapophyseal lamina, the intraprezygapophyseal lamina, and the rim of the neural canal, is present in the anterior and mid-cervicals of many theropods, including *Baryonyx* (Charig & Milner, 1997). This fossa is usually less conspicuous or even absent in more posterior cervicals. In *Ichthyovenator*, a clearly defined fossa is present in anterior cervicals, but in mid-cervicals, the fossa lacks a medial rim and is thus developed as a medially deepening depression on the pedicle defined by a sharp-rimmed centroprezygapophyseal lamina. The fossa is reduced in C7 (BK10-21), and completely lost in C8 (BK10-22) of *Ichthyovenator*. In the type of *Sp. maroccanus*, in contrast, the centroprezygapophyseal lamina is transversely broad, and a depression is absent. Even the probably most anterior cervical neural arch referred to *Sigilmassasaurus* here (the probable C4 NHMUK PV R 16427) shows a similar development of very broad centroprezygapophyseal laminae and a weakly developed or even absent fossa. In contrast, another spinosaurid mid-cervical from the ‘Kem Kem beds’ (BSPG 2006 I 57; probably C5-C6) shows a well-developed and sharply rimmed fossa.

Apart from several characters based on the erroneous assumption that the type and referred vertebrae of *Sigilmassasurus brevicollis* represent mid-cervicals (e.g., position of parapophyses, development of hypapophysis, angulation of articular ends), Russell (1996) mainly considered the extreme breadth of the vertebrae as being diagnostic for this taxon. This was the only character of the original diagnosis retained by McFeeters et al. (2013), who also noted a suite of other potential autapomorphies, including the presence of a median tuberosity on the anterior articular surface and the lack of interzygapophyseal laminae, resulting in ventrally open spinopre- and spinopostzygapophyseal fossae. However, the discovery of a complete cervical vertebral column of *Ichthyovenator* (Allain, 2014) shows that these characters are more widely distributed within spinosaurids, as the latter taxon also has extremely wide posterior cervicals and anterior dorsals, and lacks interzygapophyseal laminae in the posterior cervicals. Likewise, a median tuberosity on the anterior articular surface is present in a few other theropod taxa, such as *Aerosteon* (MCNA-PV-3137) and even, weakly developed, in *Baryonyx* (NHMUK PV R 9951). Nevertheless, the conspicuous development of this feature in all posterior cervical and anteriormost dorsal vertebrae of *Sigilmassasaurus* is unusual. A character that is unique to *Sigilmassasaurus* and can be regarded as an autapomorphy of the taxon is a strong reduction of lateral neural arch lamination, especially a massive and undivided centrodiapophyseal lamina. This lamina is divided into thin anterior and posterior centrodiapophyseal laminae in all other theropods examined, including *Ichthyovenator*. Likewise, a shallow fossa flanking the neural spine on either side of its base is present in the only ultimate cervical with a preserved neural arch (BSPG 2011 I 116) and all first dorsal vertberae in which this area is preserved (BSPG 2006 I 54, BSPG 2006 I 55, CMN 41857, NHMUK PV R 16434).

A key element is BSPG 2006 I 56, which shows proportions that are intermediate between the holotype specimens of *Sp. maroccanus* and *S. brevicollis*. It shares autapomorphic features with both holotypes, such as a transversely convex triangular posterior ventral platform that leads into the ventral keel anteriorly with *Sp. maroccanus* and the poorly developed vertebral lamination and the lack of a divided centrodiapophyseal lamina with *S. brevicollis*. Additionally, features that usually vary along the axial series, such as the development of the ventral keel or the elevation of transverse processes, indicate an intermediate position between both holotypes for BSPG 2006 I 56. Furthermore, BSPG 2006 I 56 has a strongly developed median tuberosity on the anterior articular surface, and this feature is even present, though more weakly developed in BSPG 2011 I 117, which probably represents the same vertebral position as the type of *Sp. maroccanus*. In contrast, such a median tuberosity is absent in *Ichthyovenator* (Allain, 2014), a probable mid-posterior spinosaurid cervical vertebral centrum from the ‘Kem Kem beds’ (NHMUK PV R 36637) and a probable posterior cervical vertebra from the ‘Kem Kem beds’ referred to *Spinosaurus* by Ibrahim et al. (2014a: Fig. S2C). Thus, we come to the conclusion that the material represents a single, valid taxon. Hence, we synonymize *Spinosaurus maroccanus* with *Sigilmassasaurus brevicollis*, whereby the former is a subjective junior synonym of the latter.

### Evidence for more than one spinosaurid taxon in the ‘Kem Kem beds’

Given the synonymy of *Spinosaurus maroccanus* and *Sigilmassasaurus brevicollis* established above, the question remains as to whether all of the spinosaurid material from the Kem Kem compound assemblage might be referable to this taxon, or if there is evidence for the presence of more than one spinosaurid taxon from these beds. On the basis of enamel wrinkling pattern of isolated teeth, Richter, Mudroch & Buckley (2013) recently argued that more than one taxon of spinosaurid is represented in this assemblage, and Hendrickx (2006); Hendrickx & Buffetaut (2008) came to the same conclusion based on an analysis of spinosaurid quadrates from this unit. Here we present further evidence from vertebral morphology for the presence of at least two spinosaurid taxa in the Kem Kem compound assemblage.

**Figure 17:**
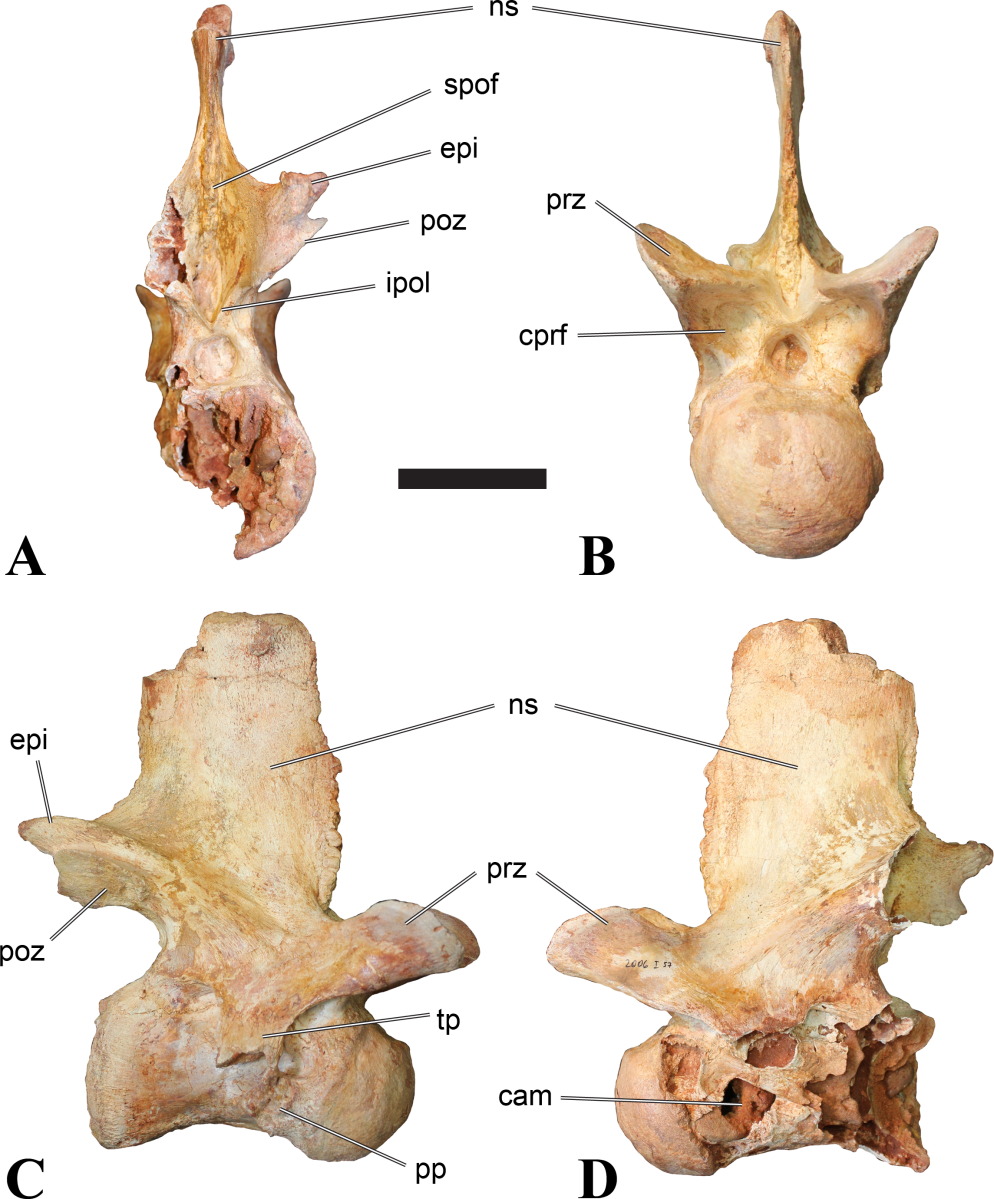
BSPG 2006 I 57, mid-cervical vertebra (C6) of an indeterminate spinosaur. (A) posterior view; (B) anterior view; (C) right lateral view; (D) left lateral view. Abbreviations: cam, camerate pneumatic chamber; cprf, centroprezygapophyseal fossa; epi, epipophysis; ipol, interpostzygapophyseal lamina; ns, neural spine; poz, postzygapophysis; pp, parapophysis; prz, prezygapophysis; spof, spinopostzygapophyseal fossa; tp, transverse process. Scale bar equals 5 cm.

Apart from the material referred to *Sigilmassasaurus*, several other cervical vertebrae of probably spinosaurid theropods from the ‘Kem Kem beds’ are present in the collections in Ottawa, London and Munich, which cannot be referred to this taxon. Several of these specimens were originally referred to *Spinosaurus maroccanus* (Russell, 1996), but as we did not study these remains first hand in the course of this study, we will mainly concentrate our discussion on material housed in London and Munich. Probable spinosaurid material not referable to *Sigilmassasaurus* kept in these institutions includes an anterior cervical vertebra (NHMUK PV R 16429), two mid-cervicals (BSPG 2006 I 57, BSPG 2013 I 97) and a posterior mid-cervical (NHMUK PV R 36637). Preservational quality of these elements is variable. The most complete element is BSPG 2006 I 57 (Fig. 17), in which the right side of the vertebra is completely preserved, with only minor parts of the posterodorsal edge of the neural spine missing; on the left side of the vertebral centrum and the left postzygapophysis are broken off. NHMUK PV R 16429 preserves the vertebral centrum, which is damaged anteroventrally, and parts of the neural arch, including the rim of the neural canal and the prezygapophyses; postzygapophyses and neural spine are missing. BSPG 2013 I 97 preserves a complete centrum and large parts of the neural arch, including the left postzygapophysis and the base of the neural spine, whereas the prezygapophyses and the dorsal part of the spine are missing. Finally, NHMUK PV R 36637 is only represented by the vertebral centrum and small portions of the lateroventral sides of the neural arch.

Referral of these elements to Spinosauridae is based on a unique character combination only found in these animals. All vertebrae are strongly opisthocoelous, as is the case in all Megalosauria and Allosauroidea. Furthermore, all vertebrae have a marked rim around the anterior articular surface, which was regarded as a megalosaurian synapomorphy by Carrano, Benson & Sampson (2012). Within megalosaurians, the vertebrae NHMUK PV R 16429, BSPG 2006 I 57 and BSPG 2013 I 97 can be referred to Spinosauridae on the basis of their strongly elongate vertebral centra, as the anterior and mid-cervical vertebrae in non-spinosaurid megalosaurs are usually short, with the centra being subequal in length to height or only slightly longer (Britt, 1991; Sereno et al., 1994; Sadleir, Barrett & Powell, 2008). NHMUK PV R 36637 is referred to Spinosauridae due to the very broad vertebral centrum, which is almost as broad as long, as in BSPG 2006 I 56 and many other spinosaurids (e.g., *Baryonyx*, Charig & Milner, 1997; *Ichthyovenator*, Allain, 2014; *Suchomimus*, MNN GDF500).

**Figure 18:**
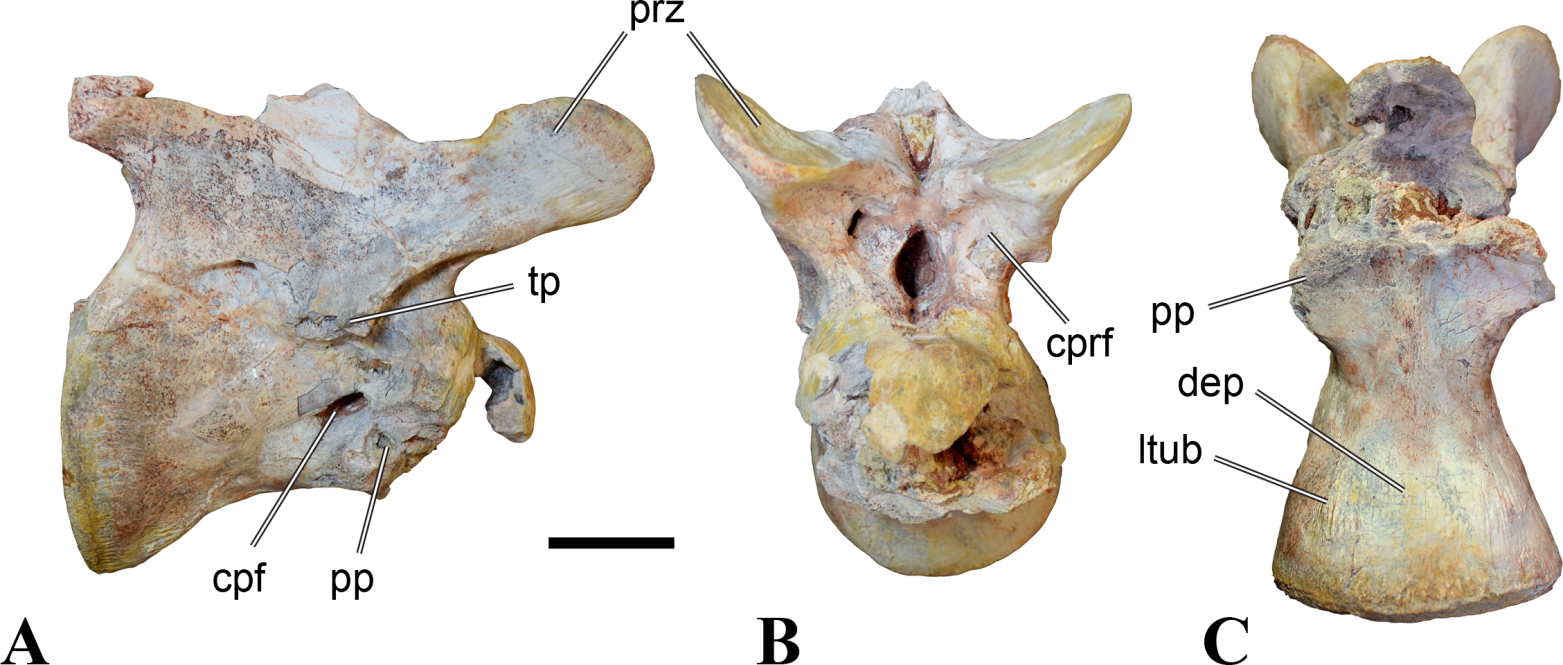
NHMUK PV R 16429, anterior cervical vertebra of an indeterminate spinosaur. (A) right lateral view; (B) anterior view; (C) ventral view. Abbreviations: cpf, central pneumatic foramen; dep, depression; ltub, lateral tubercle; pp, parapophysis; prz, prezygapophysis; tp, transverse process. Scale bar equals 5 cm.

**Figure 19:**
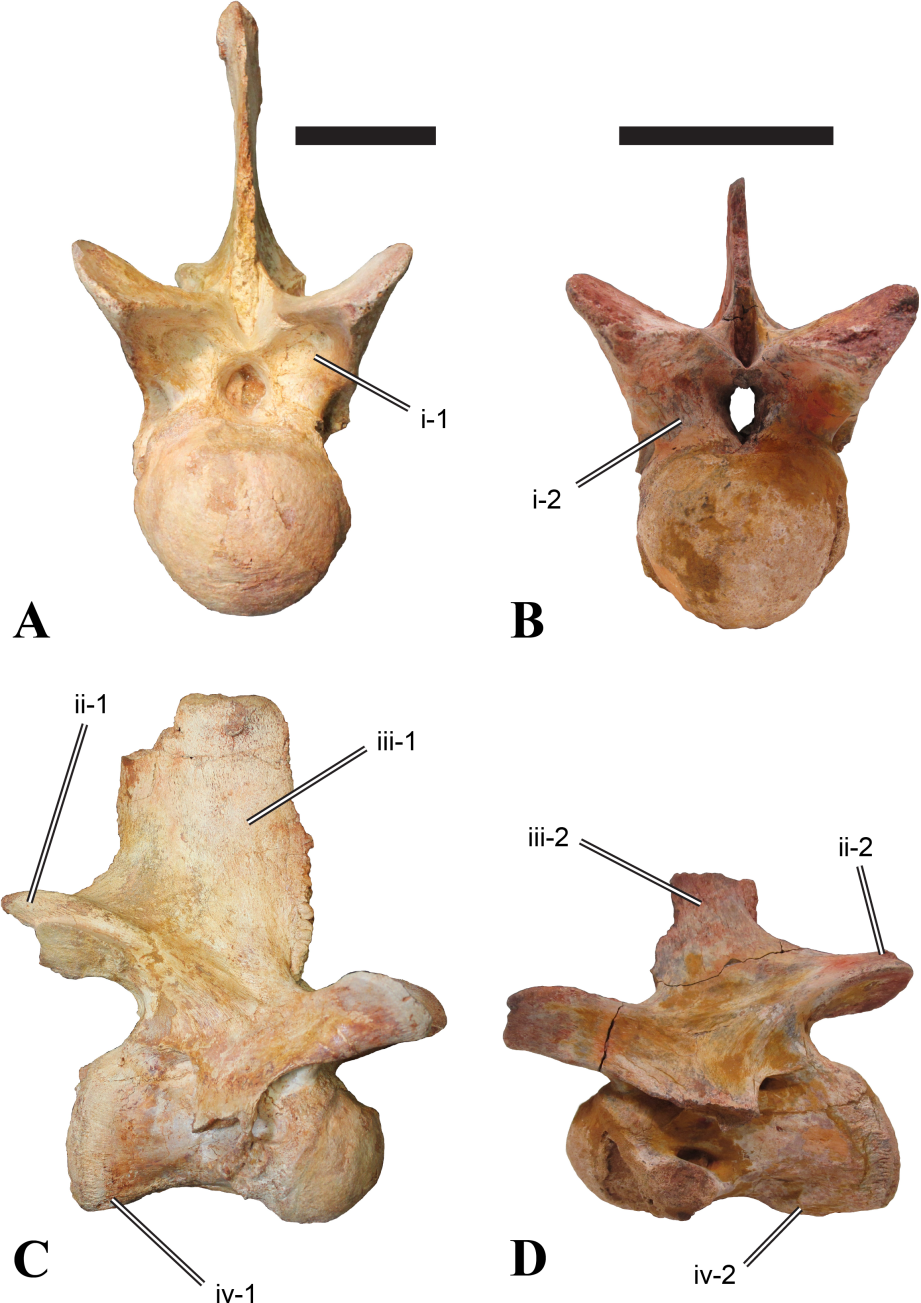
Comparisons of different spinosaurid mid-cervical vertebrae from the Kem Kem compound assemblage. (A) & (C) BSPG 2006 I 57, C6 of an indeterminate Spinosauridae; (B) & (D) CMN 50791, C6 vertebra of *Sigilmassasaurus brevicollis*. (A) & (B) anterior view; (C) right lateral view, (D) left lateral view. Labels (i–iv) indicate state of osteological features in the indeterminate spinosaur (−1) and *Sigilmassasaurus* (−2); (i) state of centroprezygapophyseal fossa (i-1: present; i-2: absent); (ii) state of epipophyses (ii-1: strongly developed; ii-2: weakly developed); (iii) state of neural spine (iii-1: anteroposteriorly long and dorsally high; iii-3: short and relatively low); (iv) state of ventral triangular plateau (iv-1: absent; iv: present). Left scale bar equals 5 cm, right scale bar equals 10 cm.

Given the incomplete preservation of several of these specimens and the sparse material available for comparison, establishing the exact vertebral position of these elements is difficult. Nevertheless, based on comparisons with *Baryonyx* and *Ichthyovenator*, NHMUK PV R 16429 (Fig. 18) most probably represents C4, as both the relative length of the vertebral centrum as well as the overall pattern of the neural arch compare well with this vertebra in these two taxa. BSPG 2006 I 57 most probably represents C6 and thus the same vertebral position as the type vertebra of *Spinosaurus maroccanus* (CMN 50791). Apart from the length–height ratio of the vertebral body (1.4), which is very similar to that of C6 of *Ichthyovenator* and of CMN 50791, the lateral neural arch lamination and the development of the prezygapophyses are closely comparable to these elements (Fig. 19). BSPG 2013 I 97 represents C6 or C7, as it has a slightly more elongate body than BSPG 2006 I 57 (with a length–height ratio of 1.63), and NHMUK PV R 36637 most probably represents C8, as indicated by the very similar proportions to BMNH 2006 I 56 and the 8th cervical of *Ichthyovenator*.

**Figure 20:**
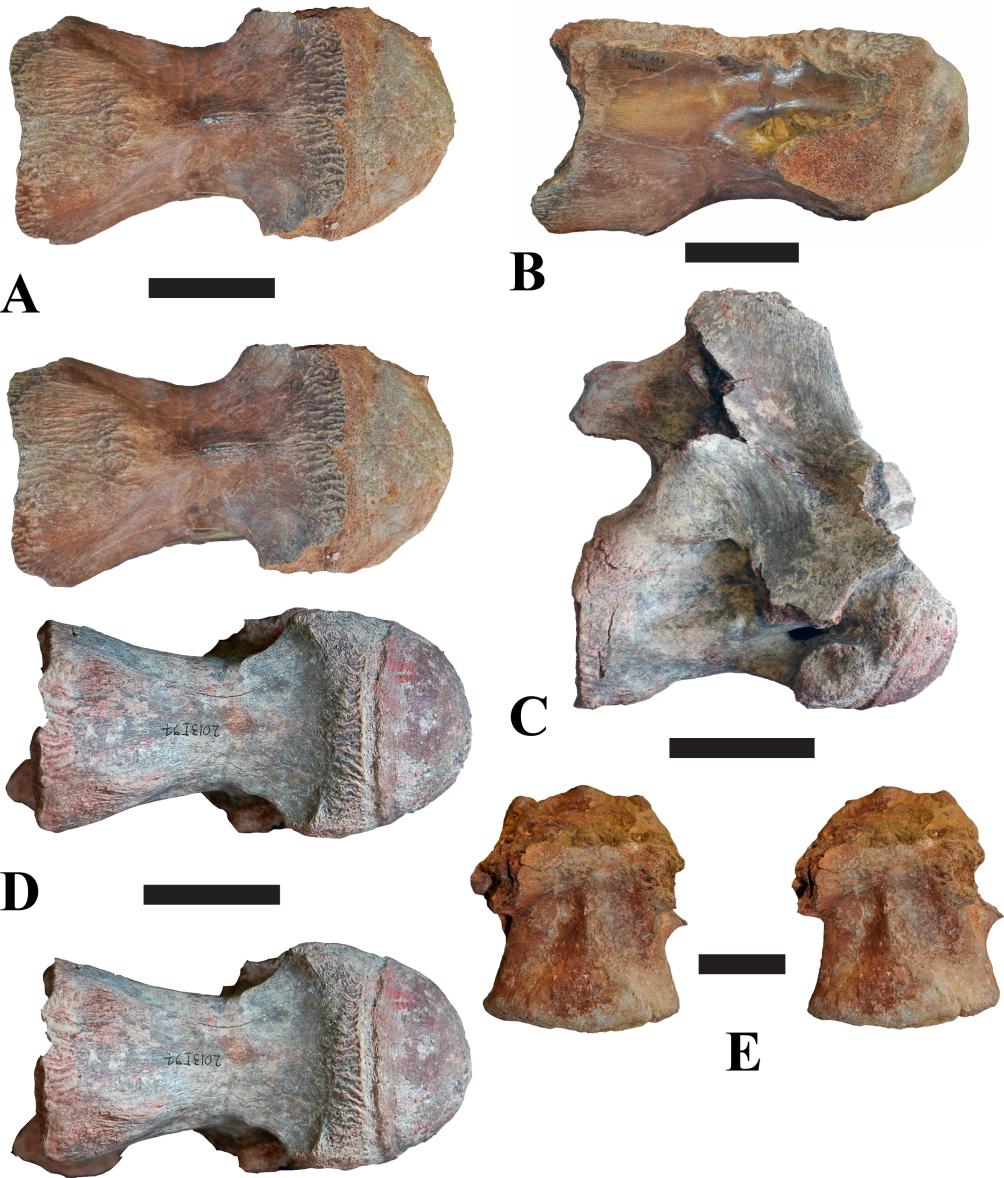
Stereographic comparisons of spinosaur cervical vertebrae from the Kem Kem compound assemblage. (A) stereopair showing ventral view of BSPG 2011 I 117, *Sigilmassasaurus brevicollis*; (B) right lateral view of BSPG 2011 I 117; (C) right lateral view of BSPG 2013 I 97; (D) stereopair showing ventral view of BSPG 2013 I 97, Spinosauridae indet.; (E) stereopair showing ventral view of BSPG 2006 I 56, *Sigilmassasaurus brevicollis*. Note triangular plateau in *Sigilmassasaurus* and prominent step in lateral outline in mid-cervicals, and ventral lateral tubercula with central depression and weak lateral step in BSPG 2013 I 97. Scale bars equal 5 cm.

**Figure 21:**
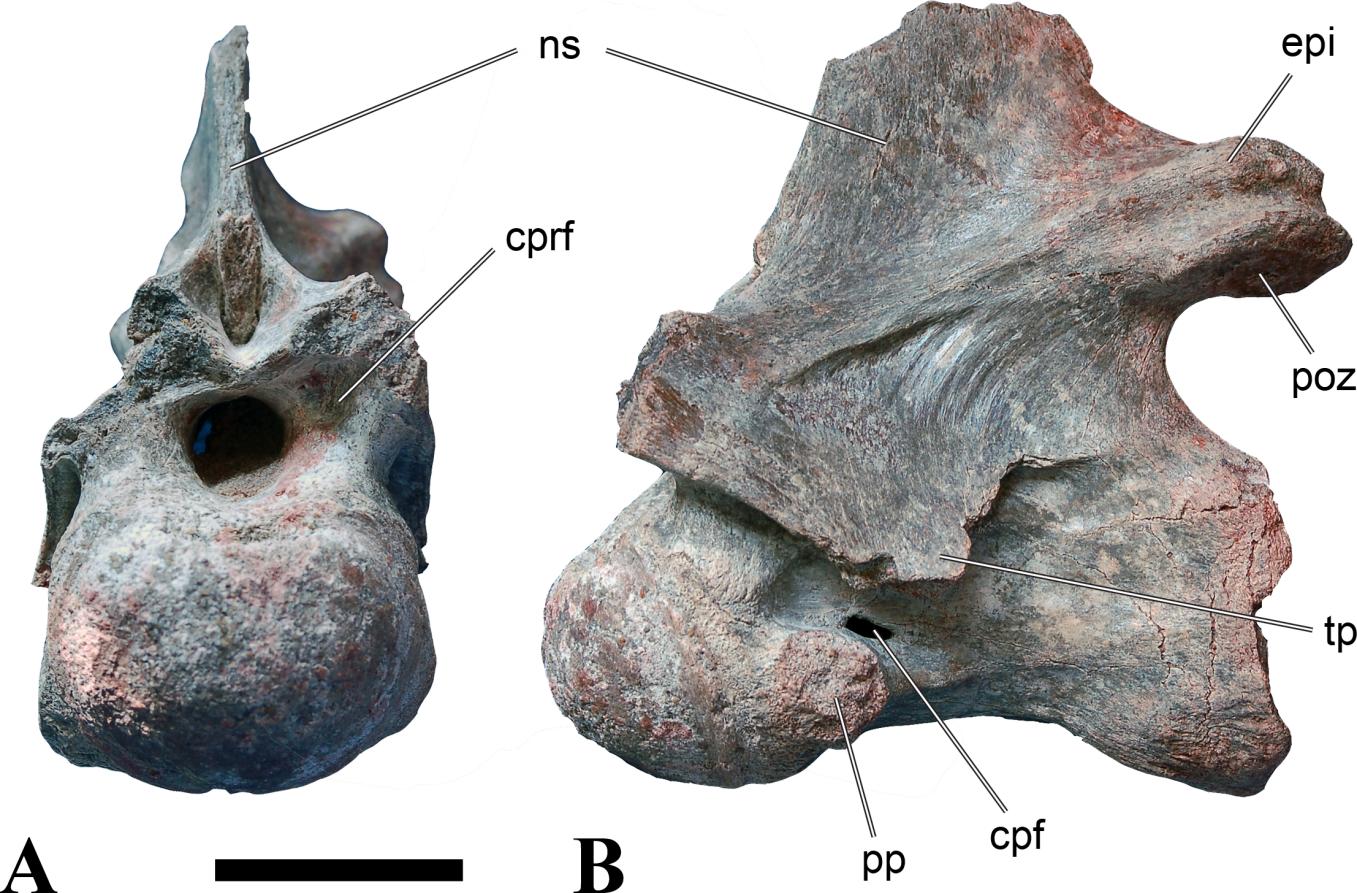
BSPG 2012 I 97, mid-cervical of an indeterminate spinosaur. (A) anterior view; (B) left lateral view. Abbreviations: cpf, central pneumatic foramen; cprf, centroprezygapophyseal fossa; epi, epipophysis; ns, neural spine; pp, parapophysis; poz, postzygapophysis; tp, transverse process. Scale bar equals 5 cm.

However, despite general similarities with the vertebrae referred to *Sigilmassasaurus* above, these specimens show significant differences, which make a referral to the same taxon unlikely. In the vertebral centra, the mid-cervical vertebrae of *Sigilmassasaurus brevicollis* show a well-developed, transversely convex plateau with a triangular outline in the posterior part of the vertebral centrum, as described above. This plateau is strongly developed in CMN 50791 and BSPG 2011 I 117 (both probably C6), but it is also present in BSPG 2011 I 118 (?C5) and BSPG 2006 I 56 (C8). In contrast, NHMUK PV R 16429 shows two low but broad, laterally paced tubercles in the posterior half of the ventral side of the centrum, which are separated by a broad depression, and the same morphology is seen in the probable C6 or C7 BSPG 2013 I 97, in which both the tubercles and the median depression are more conspicuously developed. Furthermore, whereas a median keel is present in the mid to anterior posterior cervicals of *Sigilmassasaurus* (BSPG 2011 I 118, CMN 50791, BSPG 2011 I 117 and BSPG 2006 I 56), a keel is absent in NHMUK PV R 16429 and BSPG 2013 I 97, in which only a broad, ventrally flat and slightly offset triangular area is present on the ventral side anterior to the tubercles. Due to damage to the posteroventral side of the vertebral centrum of BSPG 2006 I 57, the situation cannot be clearly established in this element, but a ventral keel is clearly absent, and there is no indication for a ventral posterior plateau, which is very conspicuous even in lateral outline in the mid-cervical vertebrae of *Sigilmassasurus brevicollis*, for instance in CMN 50791, which probably represents the same vertebral position. These differences on the ventral side, exemplified by BSPG 2006 I 56, BSPG 2011 I 117, and BSPG 2013 I 97, are illustrated in Fig. 21.

Further differences are found in a comparison of the neural arches of the mid cervicals (CMN 50791, BSPG 2006 I 57, BSPG 2013 I 97). Most conspicuous of these is the development of the epipophysis, which is high, robust and strongly overhangs the postzygapophysis posteriorly in BSPG 2006 I 57, whereas it is small, low, medially placed and does not overhang the postzygapophysis in CMN 50791. In BSPG 2013 I 97, the epipophysis is less strongly developed than in BSPG 2006 I 57 (which might be in accordance with a drastic reduction in the development of this process from C6 to C7 in *Ichthyovenator*), but it is still robust, rather high, and occupies the entire dorsal surface of the postzygapophysis (Fig. 21), which is in stark contrast to the situation in CMN 50791. On the anterior side of the neural arch pedicles, BSPG 2006 I 57 shows large and well-defined centroprezygapophyseal fossae, and this structure is also present, though less clearly defined in BSPG 2013 I 97; such fossae are entirely absent in CMN 50791. A further difference is found in the development of the neural spine, the base of which is subequal in anteroposterior length to the ventral length of the vertebral centrum in BSPG 2006 I 57 and only slightly shorter in BSPG 2013 I 97, whereas it is little more than half the length of the centrum in CMN 50791. Concerning the neural spine, a reduction from the high, rectangular and anteroposteriorly extensive neural spine of the probable C6 BSPG 2006 I 57 (and, apparently also in BSPG 2013 I 97) to the very low, spike-like spine seen in the probable C8 of *Sigilmassasaurus* BSPG 2006 I 56 seems rather unlikely.

**Figure 22:**
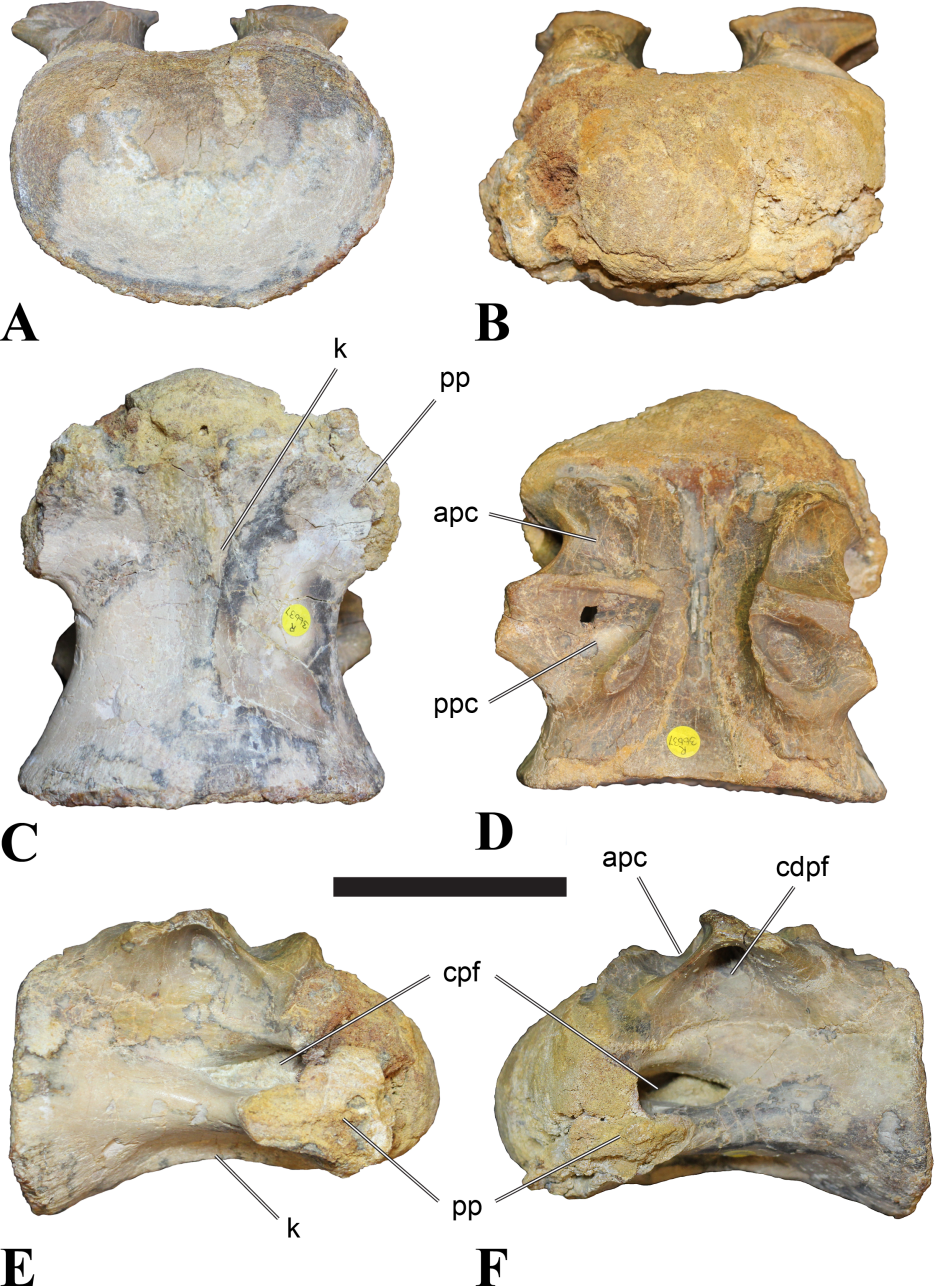
NHMUK PV R 36637, posterior mid-cervical vertebra of an indeterminate spinosaur. (A) posterior view; (B) anterior view; (C) ventral view; (D) dorsal view; (E) right lateral view; (F) left lateral view. Abbreviations: apc, anterior pneumatic chamber of the transverse process; cdpf, centrodiapophyseal fossa; cpf, central pneumatic foramen; k, keel, pp, parapophysis, ppc, posterior pneumatic chamber of the transverse process. Note that specimen is slightly dorsoventrally compressed. Scale bar equals 10 cm.

Finally, the vertebra NHMUK PV R 36637 differs significantly from BSPG 2006 I 56, which probably represents the same vertebral position, but also from more posterior cervicals and the anterior dorsals of *Sigilmassasaurus*. The ventral keel in this vertebra is low and sharply defined over most of its length, unlike the stout anterior keel that fades into a posterior platform in BSPG 2006 I 56; such a platform is missing entirely in NHMUK PV R 36637 (Fig. 22). Furthermore, in contrast to all posterior cervicals of *Sigilmassasaurus* (including BSPG 2006 I 56), the latter vertebra lacks a median tubercle on the anterior articular surface and has separated neural arch pedicles without a median suture below the neural canal. Most importantly, NHMUK PV R 36637 shows a large centrodiapophyseal fossa, delimitated by slender and well-developed anterior and posterior centrodiapophyseal laminae, whereas the absence of this fossa is an autapopmorphy of *Sigilmassasaurus* (see above).

From these comparisons, it is clear that NHMUK PV R 16429, BSPG 2006 I 57, BSPG 2013 I 97 and NHMUK PV R 36637 cannot be referred to *Sigilmassasaurus* but represent a further taxon of spinosaurid from the Kem Kem compound assemblage. The taxonomic affinities of material we interpret to represent the second Kem Kem spinosaurid is unclear; BSPG 2006 I 57 shares some characters with the C7 of FASC-KK 11888, the *Spinosaurus aegyptiacus* ‘neotype’ (e.g., the presence of centroprezygapophyseal fossae; S Evers & O Rauhut, pers. obs., 2015). Therefore, BSPG 2007 I 57 might represent the same taxon as the C7 and other material reported by Ibrahim et al. (2014a). As we question the neotype designation of these authors (see below), it is unclear if the second spinosaurid from the ‘Kem Kem beds’ can be referred to *Spinosaurus aegyptiacus*, or another, currently unrecognized taxon. Comparisons with the holotype material of *Spinosaurus aegyptiacus* show some similarities to BSPG 2007 I 57, for instance the pronounced development of epipophyses. It should be noted that all of the above mentioned features distinguish these specimens from *Sigilmassasaurus*, but are not uncommon in spinosaurids and in no case represent autapomorphic characters of *Spinosaurus aegyptiacus*. Therefore, the possibility that the non-*Sigilmassasaurus* spinosaurid is *Spinosaurus aegyptiacus* exists, but cannot be confirmed pending more material that shows shared autapomorphic features with the holotype. Note that the uncertain exact stratigraphic provenance of these remains does not necessarily mean that these spinosaur taxa were contemporaneous, although both are Cenomanian in age, and probably Early Cenomanian (Cavin et al., 2010; Le Loeuff et al., 2012). The possibility of a co-existence of two contemporaneous large spinosaur taxa might be supported by the analysis of Läng et al. (2013), who find a qualitatively homogeneous taxonomic composition of several sites tested within the Ifezouane Formation, but note compositional differences related to stratigraphic levels. However, together with the evidence for two spinosaurid taxa presented by other authors (Hendrickx, 2006; Hendrickx & Buffetaut, 2008; Richter, Mudroch & Buckley, 2013), these comparisons show that the assumption that there is a single spinosaurid taxon in the Cenomanian of northern Africa, *Spinosaurus aegyptiacus* (Ibrahim et al., 2014a), is unwarranted. Thus, caution is advisable in referring isolated remains to certain taxa known from or based on non-overlapping material (e.g., Russell, 1996; Taquet & Russell, 1998; Dal Sasso et al., 2005; Ibrahim et al., 2014a). Such referrals should always be based on anatomical arguments, as presented here, not on general stratigraphic or geographic provenance.

### “*Spinosaurus B*” and *Sigilmassasaurus*

In 1934, Stromer described a number of specimens that had been accessioned together in the Bayerische Staatssammlung für Paläontologie und Geologie under the collection number Nr. 1922 X 45. The fossils included several long bones as well as vertebral material and some teeth. Although Stromer believed these specimens to represent a single taxon (which he denominated “*Spinosaurus* B,” see below), he noted that at least the appendicular elements (parts of an ilium and leg bones) were too small to represent the same individual as the rest of the material. On the other hand, Stromer noted, “there is no reason to doubt that the teeth, vertebrae, and gastralia on one side, and the long hindlimb bones on the other side belong together” Stromer, 1934, p. 8 translated from German), although even this has been questioned by other authors (e.g., Novas, Dalla Vecchia & Pais, 2005). Unfortunately, this material was lost during WW II, as was all the dinosaur material from the Baharyia Oasis described by Stromer, so that only his accounts of these specimens remain.

Stromer’s initial assessment that the hindlimb bones must be of a different individual (if not species) than the vertebrae of Nr. 1922 X 45 is supported by comparisons of the tibia to centrum length ratios of associated theropod material. The tibia reported by Stromer only measures 600 mm, whereas the centrum of “Wirbel a” of Nr. 1922 X 45 (called anterior cervical by Stromer, but probably rather an anterior dorsal) is 117 mm long. This gives a ratio of 5.1, which is significantly beneath the values for other theropod dinosaurs (e.g., *Neovenator salerii* (MIWG 6348) has a ratio of 11 (Brusatte, Benson & Hutt, 2008); *Ceratosaurus nasicornis* Marsh, 1884 (USNM 4735) has a ratio of 9 (Gilmore, 1920)). Only if the hindlimbs were exceptionally short, as recently argued by Ibrahim et al. (2014a), could these limb bones represent the same taxon as the vertebrae. As there is considerable uncertainty about this issue (see below), we do not consider these elements further here.

Five cervical and dorsal vertebrae were reported by Stromer (1934) for 1922 X 45, designated as “Wirbel a” to “Wirbel e” for measurements see Stromer, 1934: p. 8), as well as seven caudal vertebrae (“Wirbel f” to “Wirbel m”; Stromer, 1934: p. 10). The vertebrae reminded Stromer of *Spinosaurus aegyptiacus*. Because of the proposed resemblance and the equally obvious differences to *Spinosaurus aegyptiacus* (such as the ventral keel, which is absent in the type of *Sp. aegyptiacus* (Stromer, 1934)), Stromer concluded that Nr. 1922 X 45 represented a new species of *Spinosaurus*. However, Stromer expresses his dissatisfaction with the practice of naming taxa on the basis of very fragmentary material, noting that “Den Mißbrauch aber, auf derartige, vereinzelte oder ganz unvollständige Reste neue Gattungs- oder Artnamen aufzustellen, die dann leider auf Grund der für die Paläontologie gar nicht passenden Prioritätsregeln der Nomenklatur und infolge deren geistlos pedantischer Handhabung den Ausganspunkt weiterer Benennungen zu bilden haben, mache ich nicht mit” (“I refuse to participate in the abuse of coining new genus and species names on the basis of such isolated and totally incomplete remains, which then, due to the, for palaeontology completely inadequate, priority rules of nomenclature and their senseless pedantic application, will have to be used for further nomenclatorial acts”; (Stromer, 1934, p. 6). Consequently, he did not assign a new species name to the material, but used “*Spinosaurus* B” in reference to it in the following text.

Of the presacral vertebrae, “Wirbel a,” “c,” and “d” are pictured in plates (Stromer, 1934: plate I [“Doppeltafel I”]). However, the drawing of “Wirbel c” is not very helpful, because it is a dorsal view of the centrum (to illustrate the degree of its central constriction). “Wirbel a” and “Wirbel d” are fairly well figured and described, but their different axial positions (the former considered to be an anterior vertebra, and the latter seems to be a mid dorsal vertebra) preclude direct comparisons between the two.

**Figure 23:**
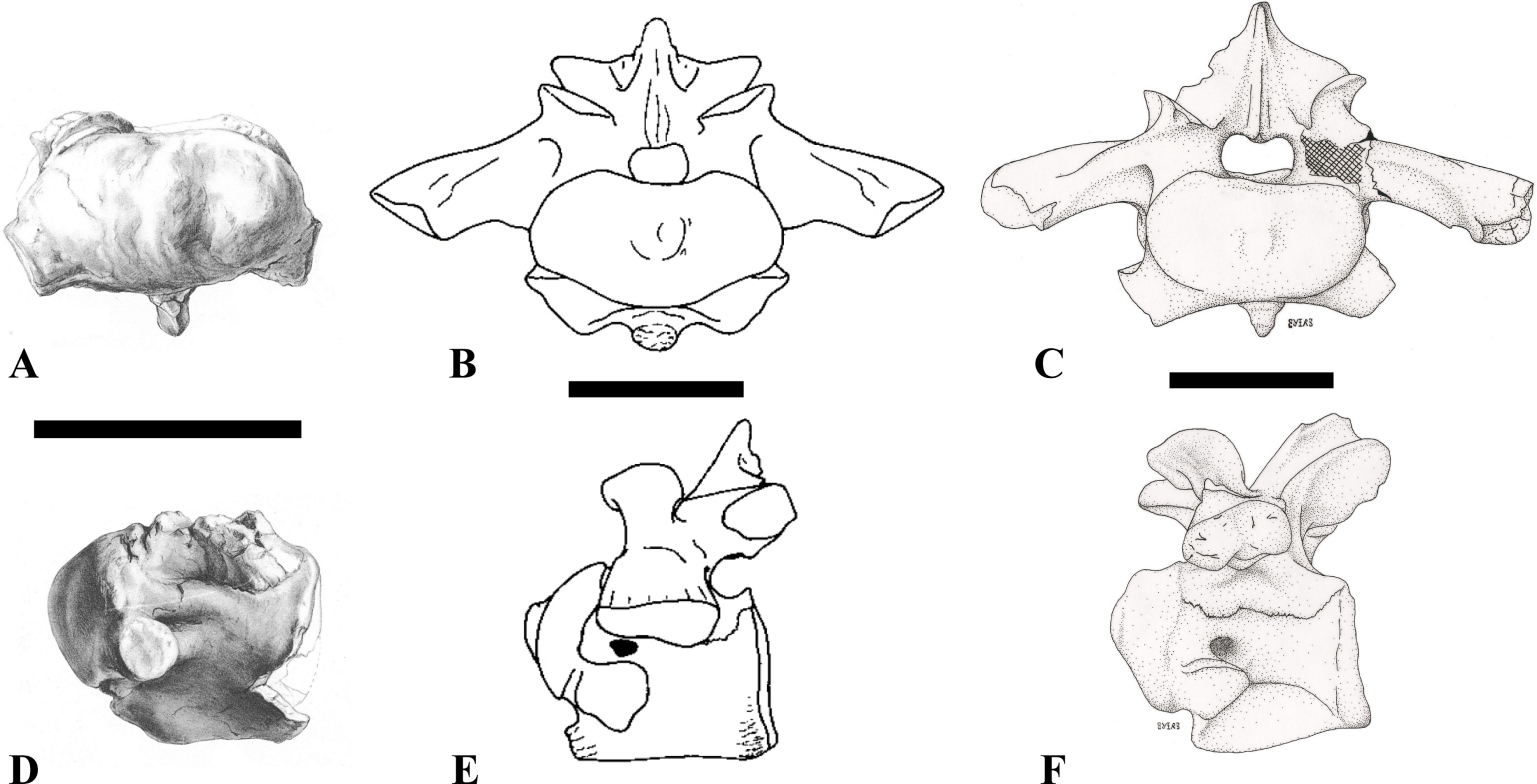
Drawings comparing “*Spinosaurus* B” and *Sigilmassasaurus*. (A) “*Spinosaurus* B” in anterior view; (B) holotype of *Sigilmassasaurus brevicollis* in anterior view; (C) BSPG 2006 I 54 in anterior view; (D) “*Spinosaurus* B” in left lateral view; (E) holotype of *Sigilmassasaurus brevicollis* in left lateral view; (F) BSPG 2006 I 54 in left lateral view. (A, C) modified from Stromer 1934. (B) E re-drawn from Russell (1996). Cross-hatching in (C) represents breakage. Scale bars equal 10 cm.

The term “*Spinosaurus* B,” although used by Stromer as a name summarizing all fossils included in Nr. 1922 X 45 and some other briefly described and not figured remains (1912 VIII 20, 21 and 22, 1911 XII 21), was later largely used in reference to the vertebral morphology represented by “Wirbel a” (see Russell, 1996). Russell noted the similarity between “Wirbel a” and CMN 41857 (the holotype of *Sigilmassasaurus*), and thus concluded that they belong to the same taxon. The resemblance of “Wirbel a” to vertebrae of *Sigilmassasaurus* is obvious from the drawings provided by Stromer (1934: pl. 1, Fig. 2; Fig. 23 this paper). Specific diagnostic features shared by the elements include the short and very broad centrum, the presence of a well-developed medial tuberosity on the anterior articular surface, and the very strongly developed and ventrally convex ventral keel. Stromer regarded “Wirbel a” as an anterior cervical vertebra based on the low position of the parapophysis on the centrum. However, as discussed above, parapophyses are retained in a low position up to the end of the cervical vertebral series in theropods, and remain low on the centrum even in the anteriormost dorsals in *Sigilmassasaurus* and other spinosaurids (e.g., *Ichthyovenator*: BK10–25). Given that the position of the parapophyses and the shape of the ventral keel are very similar to those seen in NHMUK PV R 16435, we identify “Wirbel a” as an anterior dorsal vertebra.

There are no unambiguous autapomorphies found in the descriptions and images linking the vertebrae of Nr. 1922 X 45, so that their referral to a single taxon and even individual remains uncertain. As noted above, Stromer (1934) did not doubt that all the vertebrae represented a single individual. However, the remains were found by Stromer’s collector, Markgraf, and the only information on their discovery that Stromer provided is that Markgraf reported in two letters to have found “… at the western foot of Gebel Harra, 2 km from Ain Gedîd in one spot several broken, but well-preserved bones, partially on the surface, partially in a hard, gypsum-free marl…” (Stromer, 1934: 7). Thus, it cannot be excluded with certainty that this might not simply be a chance association of vertebrae of roughly matching size. Nevertheless, the association of the vertebrae described by Stromer was obviously the incentive for Russell (1996) to refer isolated dorsal and caudal vertebrae with a morphology similar to those described and figured by Stromer (1934) to *Sigilmassasaurus*. Likewise, we found some supporting evidence to refer the anterior dorsal vertebrae BSPG 2013 I 95 and CMN 41850 to this taxon (see above). As these vertebrae are quite similar to the mid-dorsal figured by Stromer (1934: pl. 1, Fig. 4), the association of at least the presacral vertebrae of Stromer might be justified. The caudal vertebrae described as “*Spinosaurus* B” by Stromer are mid- to posterior caudals and considerably smaller than the dorsal vertebra (as would be expected for middle to posterior caudals), and have a very unusual morphology, with elongate, anterodorsally projecting prezygapophyses. Although it is suggestive that very similar vertebrae are found in the same unit as the material of *Sigilmassasaurus* (see Russell, 1996; Ibrahim et al., 2014a), and very similar caudal vertebrae are present in *Ichthyovenator* (BK 97-02), the evidence that there are at least two spinosaurid taxa in the Kem Kem compound assemblage (see above) and, combined with our still very incomplete knowledge of spinosaur caudal vertebral anatomy, precludes a secure referral to *Sigilmassasaurus* at the present. However, the close similarity of “Wirbel a” to those of *Sigilmassasaurus*, including characters that are not found in other spinosaurids, such as the strongly developed anterior tubercle, indicates that they represent at least the same genus, though there is currently insufficient evidence to refer them to the same species.

Another specimen described by Stromer (1934: 18–20) warrants some additional comments. It was only briefly described, but not illustrated, nor are there any existing photographs of these remains that we are aware of. The specimen consisted of three cervical vertebral centra, two half neural arches, a mid-section of an elongate dorsal neural spine, two ribs, and a possible distal fibula. Stromer (1934: 18–19) noted that the cervical centra were considerably wider than high, but less so than the vertebrae of his “*Spinosaurus* B” and that the ventral keel in the presumably most posterior centrum was more pronounced than in “Wirbel b” of “*Spinosaurus* B.” The dorsal neural spine was only represented by a part of the mid-section, which showed that the spine was elongate and slightly expanded dorsally. Stromer himself remarked that these remains corresponded to his “*Spinosaurus* B,” but resembled the type of *Spinosaurus aegyptiacus* in the high dorsal neural spine (Stromer, 1934: 21).

Unfortunately, the available information is insufficient to clarify the taxonomic identity of these remains. Concerning the high and dorsally slightly expanding dorsal neural spine, similar structures are known for *Suchomimus* (Sereno et al., 1998) and *Ichthyovenator* (Allain et al., 2013), so this character is now known to be more widely distributed in spinosaurids and not unique to *Spinosaurus*. As no dorsal neural spines are known for *Sigilmassasaurus* (and also not in the specimen 1922 X 45, described and figured as “*Spinosaurus* B” by Stromer), nothing can be said about the absence or presence of elongate spines in this taxon. On the other hand, spinosaur cervical and anterior dorsal vertebrae seem to be generally similar (see Charig & Milner, 1997; Allain, 2014), so that it also cannot be ruled out that Stromer (1934) erroneously compared these specimens, which were rather poorly preserved according to his comments and description, to “*Spinosaurus* B,” and these remains simply represented a smaller specimen of *Spinosaurus aegyptiacus*. Given that the specimen is lost and no further information is available, it does not help to resolve the taxonomic problems at hand.

### Distinction of *Sigilmassasaurus brevicollis* from *Spinosaurus aegyptiacus*

Recently, Ibrahim et al. (2014a) argued that *Spinosaurus maroccanus* and *Sigilmassasaurus brevicollis* as well as “*Spinosaurus* B” are referable to *Spinosaurus aegyptiacus*. However, no detailed justification for these referrals were given, and these authors did not comment on the differences between “*Spinosaurus* B” and *Spinosaurus aegyptiacus* noted by Stromer (1934; see below), nor did they provide any anatomical evidence for the referral of the two species created by Russell (1996) to *Spinosaurus aegyptiacus*. The only justification given was a notion that similar trends in positional variation of vertebrae are found in other spinosaur taxa, and a possible overlap of the proposed neotype of *Spinosaurus aegyptiacus* with the material of “*Spinosaurus* B” in respect to similar unusual limb proportions. Thus, the referral of *Spinosaurus maroccanus* and *Sigilmassasaurus brevicollis* to *Spinosaurus aegyptiacus* was essentially based on the assumption that there is a single spinosaurid taxon in the Cenomanian of all of northern Africa, which is unwarranted, as outlined above. Although the burden of proof for a synonymy of two formally named taxa is on the researchers arguing for such a proposal, we here present some comments on the differences between *Spinosaurus aegyptiacus* and *Sigilmassasaurus brevicollis*, indicating that the two represent distinct taxa. However, for several reasons, we do not accept the neotype designation proposed by Ibrahim et al. (2014a; see below), so that our comparisons are based only on Stromer’s description and figures of the holotype of *Spinosaurus aegyptiacus*. It should be noted, however, that even if the ‘neotype’ material (FSAC-KK 11888) pertains to *Sp. aegyptiacus*, several features allow a distinction of the mid-cervical included in that material and *Sigilmassasaurus brevicollis* (see below; S Evers & O Rauhut, pers. obs., 2015).

Stromer (1934: 21) noted that the remains of his “*Spinosaurus* B” showed great similarity with the holotype of *Spinosaurus aegyptiacus* in the structure of the teeth, the vertebrae and the rib. He discussed the possibility that the observed differences might only be due to sexual dimorphism or individual variation, but concluded that, with his state of knowledge, it seemed preferable to separate two taxa. In his detailed comparisons, Stromer (1934: 22) noted numerous differences in the cervical and dorsal vertebrae, such as the lack of a ventral keel and the longer and much less wide centrum in the cervicals of *S. aegyptiacus*, and the relatively shorter and slightly keeled dorsal vertebrae in “*Spinosaurus* B,” which, however, might be due to different vertebral positions being represented. He furthermore considered the neural spine of a posterior dorsal vertebra (“Wirbel d”; Stromer, 1934: pl. 1, Fig. 4) to be significantly different from those of the type of *S. aegyptiacus* in that the anterior and posterior border of the spine are parallel in “*Spinosaurus* B,” whereas the base of the spine expands in *S. aegyptiacus* (Stromer, 1915).

The original material of *Spinosaurus aegyptiacus* included two cervical vertebrae (“Wirbel a” and “b”) and seven dorsal vertebrae, besides a fragment of the left maxilla, both partial mandibles (dentaries and splenials), several teeth, a few ribs, three partial sacral, and one caudal vertebra (Stromer, 1915). In the original description, Stromer (1915) himself already expressed doubts as to whether the caudal vertebra represented the same taxon as the rest of the material, and later stated that, after viewing more dinosaur material, he was certain that this vertebra did not belong to *Spinosaurus* Stromer (1934: 6). Based on this uncertainty in the original association of the holotypic material of *Spinosaurus*, Rauhut (2003: 35–36) questioned if the presacral vertebrae and mandibles belong together, noting that the former do not show typical features seen in related taxa, such as *Suchomimus* and *Baryonyx*. However, most other authors accept the association of the material (e.g., Dal Sasso et al., 2005). Unfortunately, no other definite *Spinosaurus aegyptiacus* vertebral material has been described in the literature (Smith et al., 2006; see below), but the recently described spinosaurid *Ichthyovenator* at least confirmed that closely related animals had considerably elongated dorsal neural spines that expand dorsally (Allain et al., 2012), similar to those described for the type of *Spinosaurus*. Therefore, we follow the present consensus that the vertebrae and the mandible described by Stromer (1915) represent the same taxon, *Spinosaurus aegyptiacus*.

The two cervical vertebrae (“Wirbel a” and “Wirbel b”) were described in some detail by Stromer (1915), who also figured the neural arch of “Wirbel a” in lateral and posterior views, and “Wirbel b” in lateral view. Furthermore, both vertebrae are visible on the only existing photograph of the holotype of *Spinosaurus* (see Smith et al., 2006: Fig. 3). The centra of the two cervicals are twice as long as they are wide, and they are strongly opisthocoelous. Although the measurements taken by Stromer are incomplete, the height of the anterior articular facet exceeds its width by at least 20% in “Wirbel b” (see Stromer, 1915: 24. However, this measurement should be treated with caution, as (Stromer, 1915: 12) noted that this vertebra is compressed transversely. The ventral surfaces were described as being convex transversely and lacking keels. “Wirbel b,” which is almost completely preserved, bears an anteroposteriorly elongate pneumatic foramen posterodorsally to the parapophysis. There are two more “grooves” present on the lateral aspect of the centrum, one behind the pneumatic foramen and another posteroventral to the parapophysis. Stromer could not detect whether these two were merely depressions or small foramina that led into an internal cavity. The parapophyses are short and stout and are positioned just below the medium height on the anterior end of the centrum.

The neural arch is posterodorsally inclined. The neural canal is as high as it is wide, therefore circular in shape, yet Stromer stated that it is “conspicuously narrow” (Stromer, 1915, p. 13). The transverse processes arise from the anterior part of the pedicles that form the base of the neural arch. They project ventrolaterally and are short. The dorsal surface of the transverse processes is a flat table and the prezygodiapophyseal lamina (prdl) has a sharp edge. The only preserved prezygapophysis (on “Wirbel a”) projects anteriorly beyond the anterior margin of the neural canal. The postzygapophyses are long and bear very prominent epipophyses that overhang the postzygapophysis posteriorly, with thin epipophyseal laminae that connect with the posterior aspect of the spinal process. In “Wirbel a” these laminae connect to a roof-like structure of a posterior (spinopostzygapophyseal) fossa which “makes the neural arch appear to have been furnished with cavities” Stromer, 1915: 13; translated from German). The neural spine itself is differently developed in “Wirbel a” and “Wirbel b,” respectively. In “Wirbel a,” the neural spine rises from the entire length of the roof of the neural canal and extends beyond the spinopostzygapophyseal fossa. It emerges vertically for 3.5 cm, and then inclines posterodorsally; its dorsal part is broken off. In “Wirbel b,” the anterior edge of the spinal process seems to have been connected to the prezygapophyses by low, dorsally concave spinoprezygapophyseal laminae, above which this edge becomes vertically oriented. The posterior edge is vertically oriented from its base. The neural spine covers the posterior 8 cm of the neural arch length.

Stromer tentatively suggested “Wirbel a” to be an axis, and “Wirbel b” to be a mid-cervical. However, the identification of “Wirbel a” as axis is most probably wrong, as this vertebra has a large, dorsomedially facing and anteriorly projecting prezygapophysis, whereas axial prezygapophyses in theropods are usually developed as small, laterodorsally facing facets on the dorsal surface of the anterior end of the neural arch (e.g., Madsen, 1976). Furthermore, according to the measurements and figures given by Stromer (1915), this vertebra was approximately as large as the supposed mid-cervical “Wirbel b,” which would also be highly unusual. In comparison with *Baryonyx* (Charig & Milner, 1997) and *Ichthyovenator*, this neural arch might represent the third cervical vertebra. Characters in favour of this interpretation include the anteroposteriorly rather short neural arch and the posteriorly inclined neural spine with laterally flaring spinopostzygapophyseal laminae, which resembles the axial neural spine. In *Baryonyx* especially the neural spine of the third cervical closely resembles that of the axis (Charig & Milner, 1997). The photograph of the original *Spinosaurus* material shows that the vertebral centrum was strongly damaged, which, together with the extensive spinopostzygapophyseal laminae of the neural spine (as they are often found in the axis of basal theropods; e.g., Welles, 1984: Fig. 8) might have led Stromer to assume that this vertebra represented the axis. As for Stromer’s “Wirbel b,” we agree that this element represents a mid-cervical, based on the structure of the neural arch, mainly the high and vertical neural spine. The vertebral centrum is only slightly longer than high, resembling the condition in C5, but unlike the relatively much longer C6 and C7 of *Ichthyovenator*. Thus, this element most probably represents C5, although an identification as C4 cannot be completely excluded.

From the descriptions summarized above, the drawings provided by Stromer (1915), and the photograph of the material, several important differences with *Sigilmassasaurus* can be noticed. First, Stromer specifically noticed that the vertebrae did not have ventral keels, whereas such structures are present already in the mid-cervicals of *Sigilmassasaurus* and are especially well-developed in posterior cervicals. Likewise, a posteriorly positioned, triangular ventral platform, which is prominently developed in the mid-cervicals of *Sigilmassasaurus* (including the centrum of the possible C5 BSPG 2011 I 117) and clearly visible in lateral view (see above) is not mentioned by Stromer, nor are there any indications for such a structure in his figure of “Wirbel b” (Stromer, 1915: Table 1, Fig. 2) or the photograph. There is furthermore no indication of additional deep depressions or foramina on the posterior part of the lateral side of the centrum, as described by Stromer (1915) for “Wirbel b” and clearly visible in his illustration (Stromer, 1915: pl. 2, Fig. 2) and the only existing photograph of this element (Smith et al., 2006: Fig. 3) in any of the vertebrae referred to *Sigilmassasaurus*. Importantly, the epipophyses of both “Wirbel a” and “Wirbel b” are strongly developed and considerably overhang the postzygapophyses posteriorly (although the tip is broken in “Wirbel b”), which is not the case even in the most anterior positioned cervical vertebrae known of *Sigilmassasaurus* (the probable C4 NHMUK PV R 16427). As it is very unlikely that strongly pronounced epipophyses are present in C3 and C5, but not in C4 and from C6 onwards, this is a significant difference. Furthermore, the probable C5 of *Spinosaurus aegyptiacus* has a vertical, anteroposteriorly rather extensive and high neural spine, similar to the condition in the non-*Sigilmassasaurus* spinosaurid vertebra BSPG 2006 I 57. As the neural spine of the probable C4 of *Sigilmassasaurus* (NHMUK PV R 16427) is rather short and posteriorly inclined, and posterior cervical neural spines in this taxon are low and spike-like, such a marked variation in spine morphology within a single taxon also seems rather unlikely. Therefore, as far as comparisons are possible, the type and referred material of *Sigilmassasaurus* differs considerably from cervical material of the original specimen of *Spinosaurus aegyptiacus.* As the material also does not show any uniquely shared synapomorphies, we prefer to keep *Sigilmassasaurus* and *Spinosaurus* as distinct taxa.

### The ‘neotype’ of *Spinosaurus aegyptiacus*

In a recent paper, Ibrahim et al. (2014a) designated a neotype for *Spinosaurus aegyptiacus* (the holotype of which was destroyed in WWII) and argued that “*Spinosaurus* B,” *Sigilmassasaurus brevicollis*, and *Spinosaurus maroccanus* are referable to the former taxon. As this nomenclatorial act is relevant to the question of the taxonomic and systematic affinities of *Sigilmassasurus*, we offer here some comments on the designation of this neotype. Unfortunately, the specimen has so far been briefly described and poorly documented, so that the following comments should be regarded as tentative, pending a more comprehensive description of the material. Two of us (SWE, OWMR) have seen casts of the ‘neotype’ at the University of Chicago. As detailed above, we do not accept the synonymy of *Spinosaurus aegyptiacus* and *Sigilmassasaurus* (including *Sigilmassasaurus brevicollis* and *Spinosaurus maroccanus*), but there are further considerations that lead us to reject the specimen described by Ibrahim et al. (2014a) as neotype for *Spinosaurus aegyptiacus*.

#### Association of remains of the ‘neotype’

As acknowledged by (Ibrahim et al., 2014a, suppl. information), the allegedly associated skeleton designated as neotype was largely not excavated by the authors, but acquired from a local collector. More specifically, Cau (2014; http://theropoda.blogspot.co.uk/2014/09/spinosaurus-revolution-episodio-ii-ode.html) noted that parts of the specimen (though it was not specified which parts) were purchased in 2008 by the University of Casablanca, whereas others were acquired in 2009 by the Museo di Storia Naturale in Milan. According to Ibrahim et al. (2014a: SI, p. 6) the authors only subsequently located the collector, who showed them to the supposed locality where “complete excavation of the site by NI, PCS, CDS, DMM, SZ and colleagues resulted in the recovery of many additional pieces belonging to the neotype“ (again, these remains were not specified). Thus, unfortunately, there is no information on the original association of the remains, nor is there additional information on the locality. As most vertebrates in the ‘Kem Kem beds’ are found in multitaxonomic bone beds (Cavin et al., 2010), such information is crucial to evaluate if these remains may really pertain to a single individual. Even if it can be shown beyond a doubt that all of this material came from a single locality, it would still remain to be demonstrated that it represents a single individual. Potential evidence for association of the remains comes from the fact that the vertebrae included in this specimen are of matching size and, in the case of comparable elements (different dorsal vertebrae) matching morphology, and this is also true for the left and right femora and tibiae (S Evers & O Rauhut, pers. obs., 2015). However, it must be noted that local collectors in Morocco and fossil dealers often sort remains acquired from different sources according to fitting size and morphology (U Leonhardt, pers. comm., 2015), and an important question is, of course, if the vertebrae and the pelvic and limb elements represent the same individual.

The proportions of the new remains, specifically the disproportionally small pelvic and hindlimb remains, were used by Ibrahim et al. (2014a) to suggest very unusual leg proportions in *Spinosaurus*, noting that these unusual proportions are also found in the material of “*Spinosaurus* B,” thus allegedly supporting the association. As outlined below, however, there are significant anatomical differences between the appendicular remains of the ‘neotype’ of *Spinosaurus aegyptiacus* and the remains described as “*Spinosaurus* B,” so unless one assumes that these specimens represent closely related taxa with very similar proportions, but differences in limbbone morphology (for which there is no independent evidence), this coincidence in proportions is of doubtful value to prove association. As noted above, Stromer (1934) did not provide any evidence for original association of the remains described as “*Spinosaurus* B,” other than that they were found at the same spot (which would also be the case for non-associated materials resulting from a bone bed). However, it is striking that in both cases there is a set of matching vertebrae on the one hand, and a set of matching limb elements on the other hand.

#### Anatomical considerations

Ibrahim et al. (2014a: Fig. S2) provided a figure showing the supposed close similarity of the material of the proposed neotype to the remains described as “*Spinosaurus* B” by Stromer (1934). However, no specific characters linking the two specimens were noted, and despite some superficial similarities, there are a number of significant differences, which make a referral of these remains to a single taxon questionable. It should be noted that although the figure claims to be a comparison between the ‘neotype’ and “*Spinosaurus* B,” not all the material figured seems to pertain to the ‘neotype’ material (FSAC-KK 11888); no cervicodorsal vertebra is indicated in Fig. S3 of Ibrahim et al. (2014a), nor is such a vertebra listed in the list of materials for this specimen (Table S2), yet Fig. S2C clearly shows such an element. A cervicodorsal vertebra is also not among the casts of the ‘neotype’ kept at the University of Chicago (S Evers & O Rauhut, pers. obs., 2015). The figure caption suggests that the vertebra shown in Fig. S2C might instead be material used for the skeletal reconstruction for Fig. S3 (Ibrahim et al., 2014a). As no specimen numbers are given for the vertebrae shown in Fig. S2, their identity is unclear, but this cervicodorsal vertebra at least does not seem to belong to the ‘neotype’ material. Cervical vertebrae reported to belong to FSAC-KK 11888 are an axis and C7 (Ibrahim et al., 2014a, Table S2), so comparisons to “*Spinosaurus* B” are impossible, as the latter did not preserve these vertebral positions. Comparisons of dorsal vertebrae of “*Spinosaurus* B” and the ‘neotype,’ on the other hand, must be based on measurements given. Even the supposedly similar proportions of the limb bones of “*Spinosaurus* B” and FSAC-KK 11888 in comparison with dorsal vertebral elements are not too close: the allegedly 8th dorsal vertebra of the latter taxon is 26.9% of the length of the tibia, wheras the ratio of a posterior mid-dorsal to tibia length in “*Spinosaurus* B” is 23.3%. Though both ratios would indeed indicate extremely short hindlimbs, this difference of almost 4% is comparable to that found in theropod taxa with greatly different proportions, such as *Allosaurus* (12.3%; Gilmore, 1920) and *Elaphrosaurs* (15.6%; Janensch, 1925), and much greater than that in some phylogenetically disparate taxa, such as *Elaphrosaurus* and *Sinraptor* (15.2–15.3%; Currie & Zhao, 1993).

For non-vertebral material that does belong to the ‘neotype’ (FSAC-KK 11888), the limited comparisons that can be based on the figures provided by Ibrahim et al. (2014a) with the descriptions and figures provided by Stromer (1934) show numerous differences. Apart from differences that might be attributable to different positions within the skeleton (e.g., lack of collateral ligament grooves in pedal phalanges, less strongly curved pedal ungual, etc.), there are important differences in the limb bones.

In the distal femur of “*Spinosaurus* B,” the posteriorly projecting crista tibiofibularis is broken off, but the proximodistal length of its base is less than 75% of the distal width of the femur, as opposed to more than 100% in FSAC-KK 11888. Likewise, the proximodistal expansion of the medial condyle is much greater in the latter specimen than in 1922 X 45. An unusual feature of the femur of “*Spinosaurus* B” is a small, posterodistally pointing tubercle in the intercondylar groove, which has not been described explicitly by Stromer, but is clearly visible in both the posterior and distal views of the specimen (Stromer, 1934: pl. 1, Figs. 13B and 13D). Such a tubercle is absent in the femur figured by Ibrahim et al. (2014a: Fig. S2A). Furthermore, the lateral expansion of the lateral condyle beyond the crista tibiofibularis is much more pronounced in 1922 X 45 than in FSAC-KK 11888, and its medial side has a marked longitudinal depression (visible in Stromer’s Figs. 13A and 13D on his plate 1), resulting in the posterolateral edge of the bone forming a sharp ridge. Such a morphology is not present in FSAC-KK 11888, but the medial side is convex anteroposteriorly (Ibrahim et al., 2014a: Fig. S2A). It is also conspicuous that Stromer (1934) did not mention anything unusual about the femoral shaft of “*Spinosaurus* B,” although only the distal end of the bone is preserved, exposing a cross-section of the shaft. Given that he was aware that theropods usually have hollow limb bones (he comments on this e.g., in his description of *Carcharodontosaurus*; Stromer, 1931), but in FSAC-KK 11888, the medullary cavity is almost completely closed (Ibrahim et al., 2014a), this seems at least odd (one might argue that Stromer’s plate 1, Fig. 13B shows a thin femoral cortex at the proximal break, although this might be over-interpreting the images). Although there is certainly some possible individual variation in the morphology of the distal femur, these comparisons show that there are substantial differences between the two specimens, and no shared derived characters or a unique character combination supporting the referral to a single taxon are obvious. Given the unusually proximodistally elongate posterior crests (crista tibiofibularis and medial condyle) that are present in both femora of FSAC-KK 11888, but not in “*Spinosaurus* B,” this might be taxonomically significant.

The same is true for the tibia. According to the measurements given by Stromer (1934: 16), the width–depth ratio of the tibia at mid-shaft is 1.13 for both the right and left tibia of 1922 X 45, whereas it is 1.56 in FSAC-KK 11888 (Ibrahim et al., 2014a: Table S2). This difference in shaft proportion is quite remarkable, as it is larger than that between “*Spinosaurus* B” and most other theropods. For example, this ratio is 1.07 in *Ceratosaurus* (Madsen & Welles, 2000), 1.06 in a tibia referred to *Megalosaurus* (Stromer, 1934), 1,10 in *Piatnitzkysaurus* (PVL 4073), 1.45 in *Sinraptor* (Currie & Zhao, 1993), 1.22 in *Neovenator* (Brusatte, Benson & Hutt, 2008) and *Elaphrosaurus* (Janensch, 1925), and 1.29 in *Acrocanthosaurus* (Stovall & Langston, 1950), and only coelurosaurs have ratios similar to or larger than that of FSAC-KK 11888 (e.g., 1.51 in *Gorgosaurus* Lambe, 1917 (Lambe, 1917) and *Albertosaurus* Osborn, 1905 (Carr, Williamson & Schwimmer, 2005); approximately 2 in *Nothronychus* Kirkland & Wolfe, 2001 (Zanno et al., 2009)). On the other hand, the distal end is considerably more expanded in the Egyptian specimens than in the ‘neotype,’ with the ratio between length and distal transverse width being 4.61 and 4.65 in the former, but 5.57 in the latter. As the mid-shaft is thus more anteroposteriorly compressed in FSAC-KK 11888, but the distal end is relatively narrower transversely, these proportional differences cannot be explained by compression. Likewise, all other comparable proportions between the Egyptian and Moroccan specimens are also different, and these differences are as large or larger than between the tibiae of “*Spinosaurus* B” and other theropod taxa, so it is unlikely that they represent intraspecific variation.

Apart from these differences in proportion, the distal end of the tibiae of “*Spinosaurus* B” and FSAC-KK 11888 also show anatomical discrepancies. The most conspicuous of these are found in the distal ends of the bones. In 1922 X 45, the lateral side gradually and significantly expands distally, forming a large lateral malleolus that is triangular in outline in anterior view (Stromer, 1934: pl. 1, Fig. 14A). In contrast, FSAC-KK 11888 has an only moderately expanded lateral malleolus with a slightly convex lateral outline Ibrahim et al., 2014a: Fig. S2F). Furthermore, Stromer (1934: 17) noted that the medial malleolus was “almost rectangular” (this malleolus seems to be damaged in the element figured by Stromer), whereas it forms a tapering, medially rounded structure in FSAC-KK 11888. Although several other differences are obvious from the figures (e.g., in the shape and extent of the facet for the ascending process of the astragalus and the straight versus curved tibial shaft), these comparisons show that there are significant differences and no diagnostic shared characters.

Thus, there is reason to assume that at least the limb elements of “*Spinosaurus* B” and the specimen from Morocco do not represent the same taxon.

#### Taxonomic and formal considerations

Even if one accepts the association of the proposed neotype of *Spinosaurus aegyptiacus*, and the extremely short proportions and similarities with “*Spinosaurus* B” as evidence that both specimens represent the same taxon, the question remains, which taxon is represented by these specimens. As demonstrated above, there are several differences between the vertebrae of *Spinosaurus aegyptiacus* (as described by Stromer, 1915) and *Sigilmassasaurus maroccanus*, indicating that these remains represent different taxa, and there is also evidence that more than one spinosaur taxon is present in the ‘Kem Kem beds’ (see also Richter, Mudroch & Buckley, 2013). As noted above, the anterior dorsal vertebra figured by Stromer (1934: pl. 1, Fig. 2) is very similar to that described for *Sigilmassasaurus*, indicating that these remains represent the latter taxon. On the other hand, the proposed neotype includes neural spines that have a basal expansion (Ibrahim et al., 2014a: Fig. 2E), as it is the case in the holotype of *Spinosaurus aegyptiacus* (Stromer, 1915), but not in the dorsal vertebrae known for “*Spinosaurus* B,” as specifically pointed out by Stromer (1934: 22). Thus, if one accepts the association of the material of FSAC-KK 11888 and its identity with “*Spinosaurus* B,” the question remains whether these specimens represent *Spinosaurus aegyptiacus*, *Sigilmassasurus brevicollis*, or a further, unnamed taxon of spinosaur. Examination of a cast of the C7 of the ‘neotype’ at the University of Chicago by two of us (SWE & OWMR) showed that this vertebra has features incompatible with *Sigilmassasaurus*. The vertebra of FSAC-KK 11888 shows centroprezygapophyseal fossae with relatively sharp lateral and dorsal borders, well-developed interpostzygapophyseal laminae, and lacks a ventral triangular plateau or keel.

This leads to the final objection to the neotype designation by Ibrahim et al. (2014a). As outlined by these authors Ibrahim et al., 2014a: supplements p. 6–7), the ICZN requires several conditions to be met for a neotype designation. We note that several of these conditions are not met with the designation of FSAC-KK 11888: the secure identification of the proposed neotype as the same taxon as the holotype, an exceptional need for the designation of a neotype, and the geographical proximity of the locality of the neotype to the original type locality. The first objection is clear from the different considerations on association, anatomy, and taxonomy of the specimen presented above. As for an exceptional need for a neotype, it is clear that the holotype of *Spinosaurus aegyptiacus* was destroyed in WW II, but the material was relatively well-described and figured by Stromer (1915). Both the descriptions and the figures provided by Stromer indicate a number of potentially autapomorphic characters, such as a change from very large anterior teeth to minute middle teeth to again large posterior teeth in the dentary (Stromer, 1915: pl. 1, Fig. 12B), an extremely enlarged, anteroventrally open anterior myliohyoid foramen in the splenial (Stromer, 1915: pl. 1, Fig. 6), and the strong, rounded expansion at the base of the neural spines (Stromer, 1915: pl. 1, Figs. 17–19, pl. 2, Figs. 3–5). Even if there might be some doubt about the uniqueness of some of these characters, such as the basal expansion of the neural spines (see above), the identity of *Spinosaurus aegyptiacus* can clearly be established from the combination of numerous unusual characters, based on Stromer’s description and figures. Thus, we do not see an exceptional need for a neotype designation.

Concerning the proximity of the locality of the proposed neotype to the original type locality, the distance of the Kem Kem area in Morocco from the Baharyia Oasis in Egypt is roughly 3,200 km in a straight line. Apart from the geographic distance, it is somewhat unclear in how far the fauna from the Baharyia Oasis is directly comparable to that of the Kem Kem compound assemblage. Although great similarity exists on higher systematic levels (see Cavin et al., 2010), only few shared species are actually present, especially in tetrapods (contra Le Loeuff et al., 2012). In crocodiles, for example, many forms are similar and might represent sister taxa, but no shared species are present (see Larsson & Sues, 2007; Sereno & Larsson, 2009; Holliday & Gardner, 2012). In dinosaurs, the only other taxon said to be present in both the Baharyia Oasis and the ‘Kem Kem beds’ is *Carcharodontosaurus saharicus* (Sereno et al., 1996). The syntypes of this taxon are two isolated teeth from the (?)Albian of the area around Timimoun, western Algeria (Depéret & Savornin, 1927), which are now seemingly lost (see Brusatte & Sereno, 2007), so Brusatte & Sereno (2007) proposed an almost complete skull from the ‘Kem Kem beds’ area as neotype. Stromer (1931) described a fragmentary skeleton from Baharyia also as *Carcharodontosaurus saharicus*, due to the similarity of the teeth to those described by Depéret & Savornin (1927). However, teeth with the characteristics described by Depéret & Savornin (1927) are also found in other carcharodontosaurid taxa (see Brusatte & Sereno, 2007), and no detailed comparison of the skull from the ‘Kem Kem beds’ with the few skull elements recovered by Stromer (1931) from Baharyia (which are also lost) has ever been presented. Thus, it remains to be shown that the latter remains are really conspecific with the specimen from the ‘Kem Kem beds,’ or if they might represent closely related, but separate species. Apart from *Carcharodontosaurus* and *Spinosaurus*, three additional nominal species of dinosaurs are known from Baharyia, the theropod *Bahariasaurus ingens*, and the sauropods *Aegyptosaurus aegyptiacus* Stromer, 1932 and *Paralatitan stromeri* Smith et al., 2001 (Stromer, 1932; Stromer, 1934; Smith et al., 2001); none of these taxa have been identified from the ‘Kem Kem beds.’ Likewise, none of the other nominal dinosaur species in the ‘Kem Kem beds,’ including the theropod *Deltadromeus agilis,* the probable carcharodontosaurid *Sauroniops pachytholus* Cau, Dalla Vecchia & Fabbri, 2013, and the sauropod *Rebbachisaurus garasbae* Lavocat, 1954 (Lavocat, 1954; Sereno et al., 1996; Cau, Dalla Vecchia & Fabbri, 2013), has been reported from Baharyia. Thus, although similar in general taxonomic composition, there is actually little overlap on specific level between the two localities, making a neotype designation from such a geographically distant locality dubious.

### Synonymy of *Sigilmassasaurus* with *Carcharodontosaurus saharicus*

Soon after it was proposed as a new taxon, *Sigilmassasaurus* was suggested to be a subjective junior synonym of *Carcharodontosaurus* (Sereno et al., 1998; Brusatte & Sereno, 2007). This taxonomic opinion was partly based on a proposed overlap between associated material of *Carcharodontosaurus* described by Stomer in 1931 as 1922 X 46, and the material Stromer (1934) described as “*Spinosaurus* B” (see Sereno et al., 1996), which has commonly been interpreted to be material of *Sigilmassasaurus*. The specimen of *Carcharodontosaurus* (1922 X 46) described by Stromer (1931) included three cervical vertebrae, of which “Halswirbel b” is the only detailed described and figured postaxial vertebra, allowing comparison to *Sigilmassasaurus* vertebrae. However, the vertebra in question shows significant differences to vertebrae of *Sigilmassasaurus*. Measuring 88 mm across the anterior articular surface of the centrum and 100 mm in length, the centrum of the vertebra is slightly longer than wide. Also, the width–height ratio of “Halswirbel b” equals 1.3, which is much lower than the ratios of >1.5 used to identify *Sigilmassasaurus* (Russell, 1996; McFeeters et al., 2013). These observations do not match the morphology seen in the posterior cervicals and anterior dorsals of *Sigilmassasaurus,* to which this material was compared by Sereno et al. (1996). Furthermore, “Halswirbel b” was originally identified as an anterior cervical by Stromer (1931), and some features like the relative offset between anterior and posterior interarticular facets suppot this identification. Accordingly, comparisons between the “Halswirbel b” of *Carcharodontosaurus* and posterior vertebrae of *Sigilmassasaurus* are inadequate. In comparison with more anterior cervical vertebrae of *Sigilmassasaurus*, such as CMN 50791 or BSPG 2011 I 117, the most striking difference is the extreme shortness of the vertebra of *Carcharodontosaurus*: in the latter, the ratio of centrum length to anterior centrum width is 1.13, whereas it is approximately 2 in BSPG 2011 I 117.

**Figure 24:**
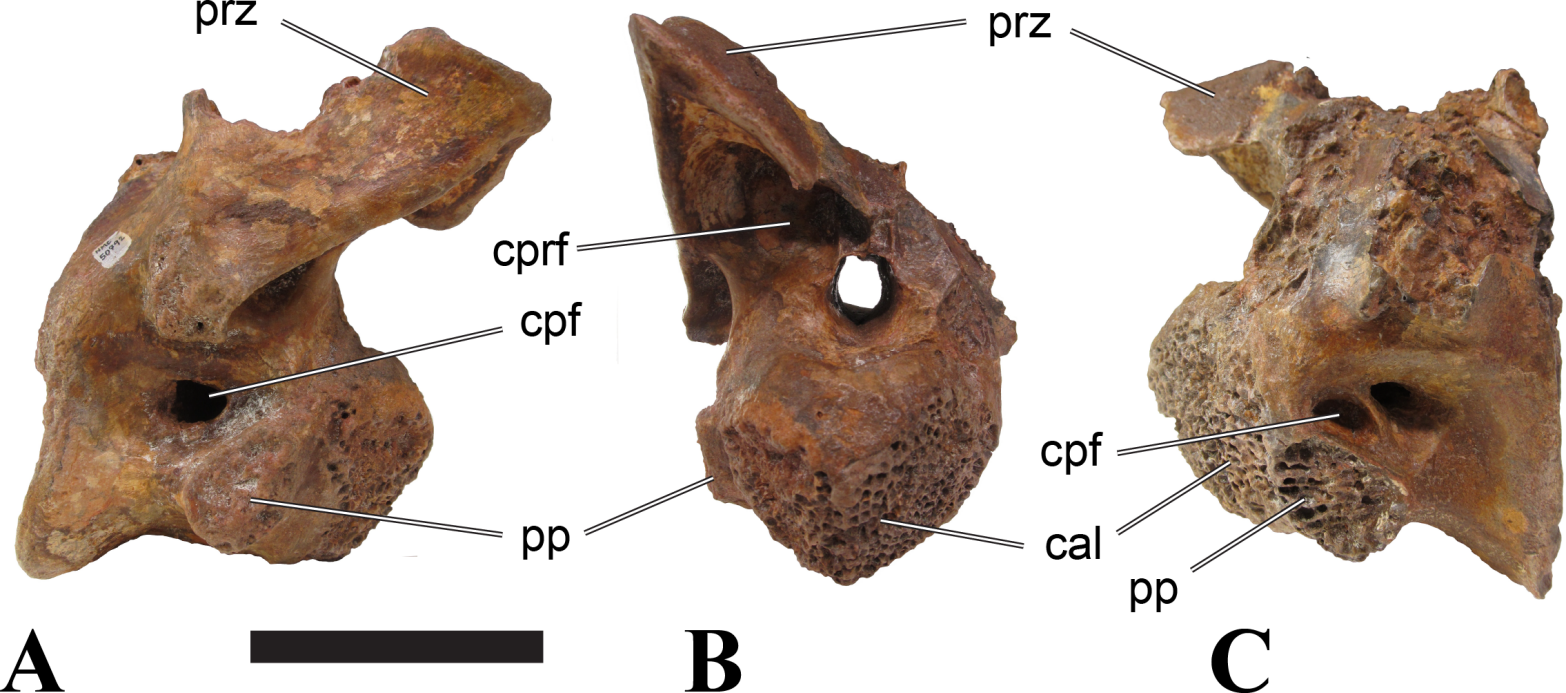
CMN 50792, posterior cervical vertebra of an indeterminate carcharodontosaurid. (A) right lateral view; (B) anterior view; (C) left lateral view. Abbreviations: cal, camellate pneumatic structure; cpf, central pneumatic foramen; cprf, centroprezygapophyseal fossa; pp, parapophysis; prz, prezygapophysis. Scale bar equals 10 cm.

Furthermore, as pointed out by Novas et al. (2005), Novas et al. (2013) and Canale, Novas & Haluza (2008), known cervical vertebrae of South American carcharodontosaurids differ considerably from vertebrae of *Sigilmassasaurus*. A well-preserved cervicodorsal vertebra of *Mapusaurus* (MCF-PVPH-108.82; probably one of the first three dorsal vertebrae, based on the position of the parapophyses; Coria & Currie, 2006) is markedly different from *Sigilmassasaurus* vertebrae thought to occupy similar axial positions. Among the differences are a rounded outline of the anterior condyle of the centrum, the lack of a ventrally expanded hypapophysis, presence of two separated pneumatic formanina on the lateral side of the centrum, well-developed laminae on the lateral side of the neural arch, relatively short transverse processes, presence of interzygapophyseal laminae, presence of a hyposphene, and large, laterally extensive spinopostzygapophyseal laminae (Coria & Currie, 2006). Additionally, *Mapusaurus* has a high and anteroposteriorly very short neural spine, deep centroprezygapophyseal fossae (anterior infraprezygapophyseal fossae of Coria & Currie, 2006) on the anterior side of the prezygapophyseal pedicles, and a relatively small, rounded neural canal. Similarly, *Sigilmassasaurus* can be easily distinguished from *Tyrannotitan*, which preserved on postaxial cervical, interpreted as a seventh cervical (MPEF-PV 1157; Novas et al., 2005; Canale et al., 2014). Since *Sigilmassasaurus* displays many differences to well-known carcharodontosaurids, the proposed overlap between “*Spinosaurus* B” and *Carcharodontosaurus* is not supported by the available evidence, and there is good evidence for spinosaurid affinities for the former taxon, a synonymy of *Sigilmassasaurus* with *Carcharodontosaurus* can be rejected. Furthermore, there is no conclusive evidence for carcharodontosaurid affinities from comparisons with other members of this clade. Within the Kem Kem compound assemblage, vertebrae of a more ‘typical’ carcharodontosaurid morphology have been found (e.g., CMN 50792, Fig. 24), offering alternatives for cervical vertebral material of *Carcharodontosaurus*.

The best evidence is probably the close overlap and the synapomorphic features shared by “*Spinosaurus* B” and *Ichthyovenator*, the latter being a Spinosauridae.

### Systematic affinities of *Sigilmassasaurus*

Since its original description (Russell, 1996), *Sigilmassasaurus* has generally been regarded as a tetanuran theropod (e.g., Sereno et al., 1996; Sereno et al., 1998; McFeeters et al., 2013). Only Canale, Novas & Haluza (2008), in a published abstract, suggested that this taxon might actually be a large ornithopod, based on similarities of the vertebrae with those of *Iguanodon* Mantell, 1825, which also has very broad cervical centra, a well-developed ventral keel and poor lateral lamination. However, these characters have a wider distribution in dinosaurs and are also found in several theropod lineages. *Sigilmassasaurus* can be identified as a member of Tetanurae based on several synapomorphic features, including presacral vertebrae with a convex anterior condyle, a single pneumatic opening on the lateral side of the centrum, and widely spaced prezygapophyses (see also McFeeters et al., 2013).

Establishing the systematic position of this taxon within Tetanurae is more difficult, given that only cervical and anterior dorsal vertebrae can be referred to *Sigilmassasaurus*. Theropod material from the Kem Kem compound assemblage, including common occurences of shed teeth, has been assigned to Carcharodontosauria (e.g., Sereno et al., 1996; Cau, Dalla Vecchia & Fabbri, 2013), Spinosauridae (e.g., Dal Sasso et al., 2005; Ibrahim et al., 2014a), Ceratosauria (Russell, 1996; Sereno et al., 1996; Mahler, 2005; Carrano & Sampson, 2008; Porchetti et al., 2011; Richter, Mudroch & Buckley, 2013; Evans et al., 2015), and Dromeosauridae (Amiot et al., 2004; Richter, Mudroch & Buckley, 2013). Ceratosaurian and abelisaurid affinities of *Sigilmassasaurus* are unlikely given the tetanuran synapomorphies present in the vertebrae of this taxon and numerous differences with the vertebrae of other known ceratosaurs and abelisaurids (e.g., Gilmore, 1920; Madsen & Welles, 2000; O’Connor, 2007; Méndez, 2012). As noted above, carcharodontosaurid affinities, as suggested by Sereno et al. (1996), Sereno et al. (1998) and Brusatte & Sereno (2007) are also unlikely. *Sigilmassasaurus* material does not pertain to Dromeosauridae, as is lacks synapomorphies of Coelurosauria (such as amphiplatyan cervical vertebrae, Turner, Makovicky & Norell, 2012). As further outlined above, differences between the vertebrae of the holotype of *Spinosaurus aegyptiacus* and the vertebrae of *Sigilmassasaurus* preclude a referral of the latter to the former, but *Sigilmassasaurus* might represent a distinct taxon of spinosaurid.

Within spinosaurids, *Sigilmassasaurus* can best be compared with *Baryonyx* and *Ichthyovenator*, because large parts of the presacral vertebral column are known and detailed descriptions and images have been published for *Baryonyx* (Charig & Milner, 1997), and one of us (RA) has access to recently discovered cervical and dorsal material pertaining to the holotype specimen of *Ichthyovenator* (Allain, 2014). *Baryonyx* and *Suchomimus* have been recovered as sister taxa in latest phylogenetic analyses, and form the monophyletic Baryonynchinae within the Spinosauridae (e.g., Carrano, Benson & Sampson, 2012), in which the Asian *Ichthyovenator* was included (Allain et al., 2012). For *Suchomimus*, only a short initial report has been published (Sereno et al., 1998), and a detailed description of vertebral material is lacking. However, some comparative notes can be included here based on observations of the holotype material (by SWE & OWMR).

The other spinosaurid clade Spinosaurinae includes the genera *Spinosaurus*, *Irritator*, and *Angaturama* Kellner & Campos, 1996 (Carrano, Benson & Sampson, 2012), of which only *Spinosaurus* has vertebral material than could be compared to *Sigilmassasaurus* (see above).

The holotype material of *Baronyx* (NHMUK PV R 9951) includes remains of 17 presacral vertebrae. While only five cervical vertebrae were originally recognized within the holotype material, we argue that the vertebrae initially identified as D1 and D2 also represent cervical elements. Although Charig & Milner (1997: 30) argued that the typical number of cervical vertebrae in theropods was 9, we consider non-avian theropod dinosaurs to generally have ten cervical vertebrae. This is based on observations on well represented or articulated specimens, in which the first vertebra bearing a dorsal rib is the 11th presacral vertebra, such as *Eodromaeus* Martínez et al., 2011 (Martínez et al., 2011), *Coelophysis* Cope, 1889 (BSPG cast of AMNH 7224), *Sciurumimus* Rauhut et al., 2012, BMMS BK 1, *Sinraptor* (Currie & Zhao, 1993), *Allosaurus* (SMA 0005; contra Gilmore (1920) and Madsen (1976)), and *Sinosauropteryx* Ji & Ji, 1996 (Currie & Chen, 2001). As noted above, the change from cervical to dorsal vertebrae is marked with a notable dorsal displacement of the parapophysis in theropods. In most theropods, the parapophysis still lies on the anteroventral end of the centrum in C10, but on or above the mid-height of the anterior rim of the centrum in D1, often straddeling the neurocentral suture. However, in some taxa, the displacement is less marked, and the anterior dorsals have the parapophysis on the ventral part of the anterior end of the centrum, but slightly above the ventral margin. Furthermore, the transverse process is usually strongly ventrolaterally directed in mid-cervical vertebrae and becomes more lateral in posterior cervicals, until it achieves a more or less horizontal position in the first dorsal vertebrae.

In *Baryonyx*, Charig & Milner (1997) identified the axis and four further cervical vertebrae as C3, 5, 6 and 8. They also identified a continuous series of anterior dorsal vertebrae, ranging from D1 to D8. However, based on their overall morphology and in comparison with other theropods, we argue that the five cervicals identified by Charig & Milner (1997) probably represent C2 (axis) to C6, with the differences in the dimensions of the centra being largely due to deformation of the elements.

**Figure 25:**
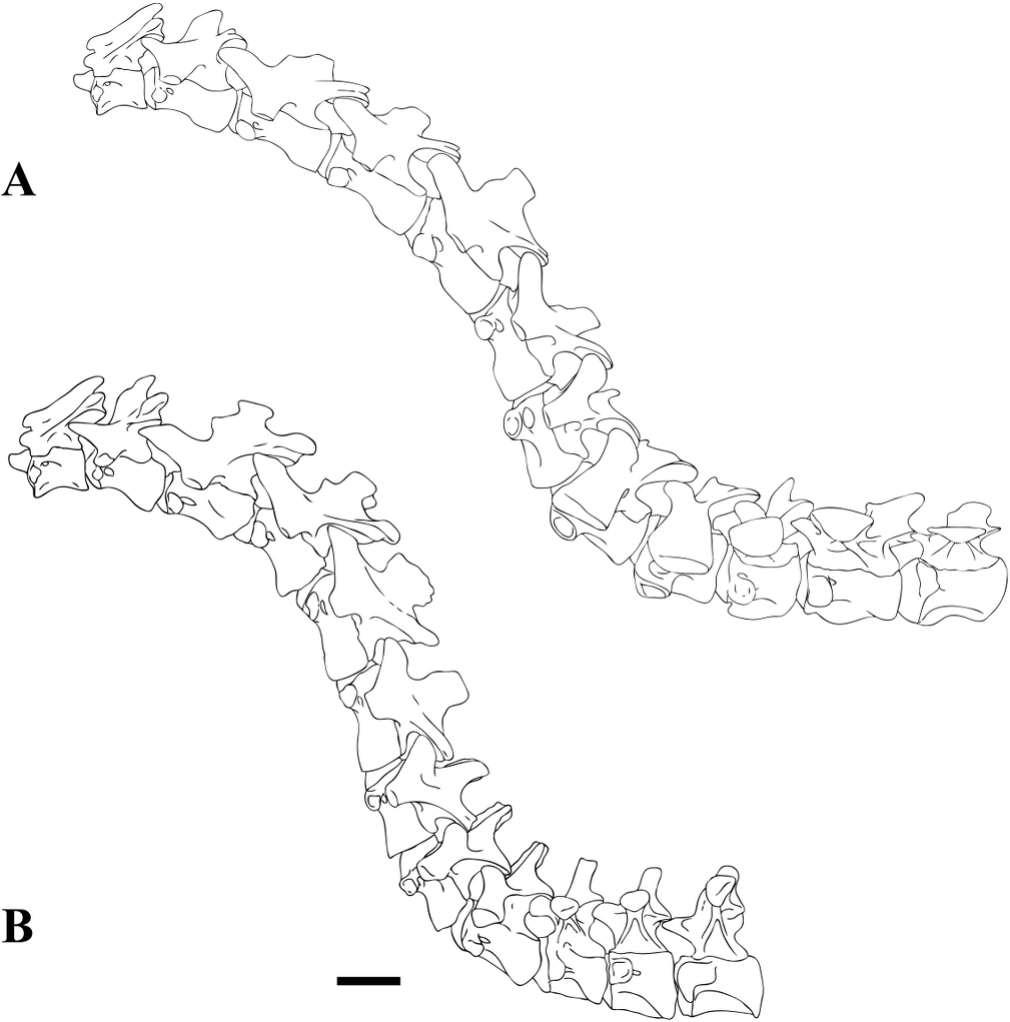
Neck reconstructions of *Sigilmassasaurus* and *Baryonyx*. (A) *Sigilmassasaurus*; (B) *Baryonyx*. Scale bar equals 10 cm. Scale bar only applies to *Baryonyx*, for which associated material is preserved. Reconstructions are based on the holotype material for *Baryonyx*, and material discussed in this paper for *Sigilmassasaurus*. Note that the first vertebra depicted is the C2 (axis), and that reconstruction includes anterior dorsal vertebrae.

Based on this count, we think the originally assigned positions C2, C3, C5, C6, C8, D1, and D2 represent cervical vertebrae C2, C3, C4, C5, C6, C9, and C10, respectively. Consequentially, we disagree with some of the dorsal placements propsed by Charig & Milner (1997). However, as in Charig & Milner (1997), we recognize continuous morphological trends in the anterior dorsal vertebrae, so that we agree that they represent a successive series. Hence, the originally assigned positions D3 through D8 represent D1 through D6. Originally assigned D10 through D14 represent D9 through D13, as we agree that the posterior most preserved dorsal vertebra is the last presacral vertebra. Figure 25 shows a reconstruction of the neck and anterior parts of the dorsal vertebral series of *Sigilmassasaurus* and *Baryonyx*, and follows our new vertebral assignment for *Baryonyx*.

The new identifications are based on the following observations: the first vertebra with slightly elevated parapophyses is Charig & Milner’s D3, here identified as D1; the first vertebra with horizontally oriented transverse processes in Charig & Milner’s D3, here identified as D1; increased central length toward mid-cervical vertebrae and following length reduction together with increased centrum width, as well as transition from blade-like, transverse thin neural spine to vertically oriented, rod-like neural spine is in agreement with cervical morphology transitions in articulated or associated specimens of other theropod taxa, including *Allosaurus* (DINO 11541, SMA 0005), *Acrocanthosaurus* (OMNH 10146, formerly MUO 8-0-S9, Stovall & Langston, 1950), *Majungasaurus* (UA 8678, O’Connor, 2007), or *Sinraptor* (IVPP 10600, Currie & Zhao, 1993). The assignment of following dorsal vertebrae of *Baryonyx* is in agreement with observations on SMA 0005, which is an *Allosaurus* specimen preserving the entire presacral series. In *Baryonyx*, D4 (“D6”) is the first vertebra with the parapophyses being located entirely on the neural arch, as in SMA 0005. Also as in *Baryonyx*, the degree of opisthocoely levels off in the anterior dorsal series of *Allosaurus*, with D1 being the first dorsal with a n oticeable decrease in the convexity of the anterior articular condyle. In SMA 0005, the third dorsal vertebra is the first one in which the anterior articular facet is truly flat. The appearance of a flat surface is delayed in *Baryonyx* to the D4 (“D6”). In general, the continuous dorsal assignment for the anterior and posterior dorsals proposed by Charig & Milner (1997) is followed here. In the following, referral to *Baryonyx* vertebrae will follow our new identification sheme.

When the cervical vertebrae of *Baryonyx* are aligned according to our new positional designations, the neck does assume a sigmoidal shape, *contra* the original description in which *Baryonyx* is described as having an uncurved neck (Charig & Milner, 1986; Charig & Milner, 1997). The curvature is less intense than in some theropods, such as *Sinraptor* (Currie & Zhao, 1993), and instead of anterior and posterior intercentral articulations that are offset to one another, the curvature is achieved by the inclination of those facets. This condition is similar to *Sigilmassasaurus*, in which the mid-cervicals (e.g., CMN 50791, BSPG 2011 I 117) have anteroventrally inclined anterior facets like C6 of *Baryonyx*, and also *Ichthyovenator*, and posterior cervicals and anterior dorsals (e.g., BSPG 2006 I 54) have strictly anteriorly facing facets like in anterior dorsals of *Baryonyx* and *Ichthyovenator*. *Suchomimus* is slightly different in this respect, as the intercentral articualtions show a clear offset to one another in both the posterior cervical and anterior dorsal vertebrae of the holotype (MNN GDF500).

**Figure 26:**
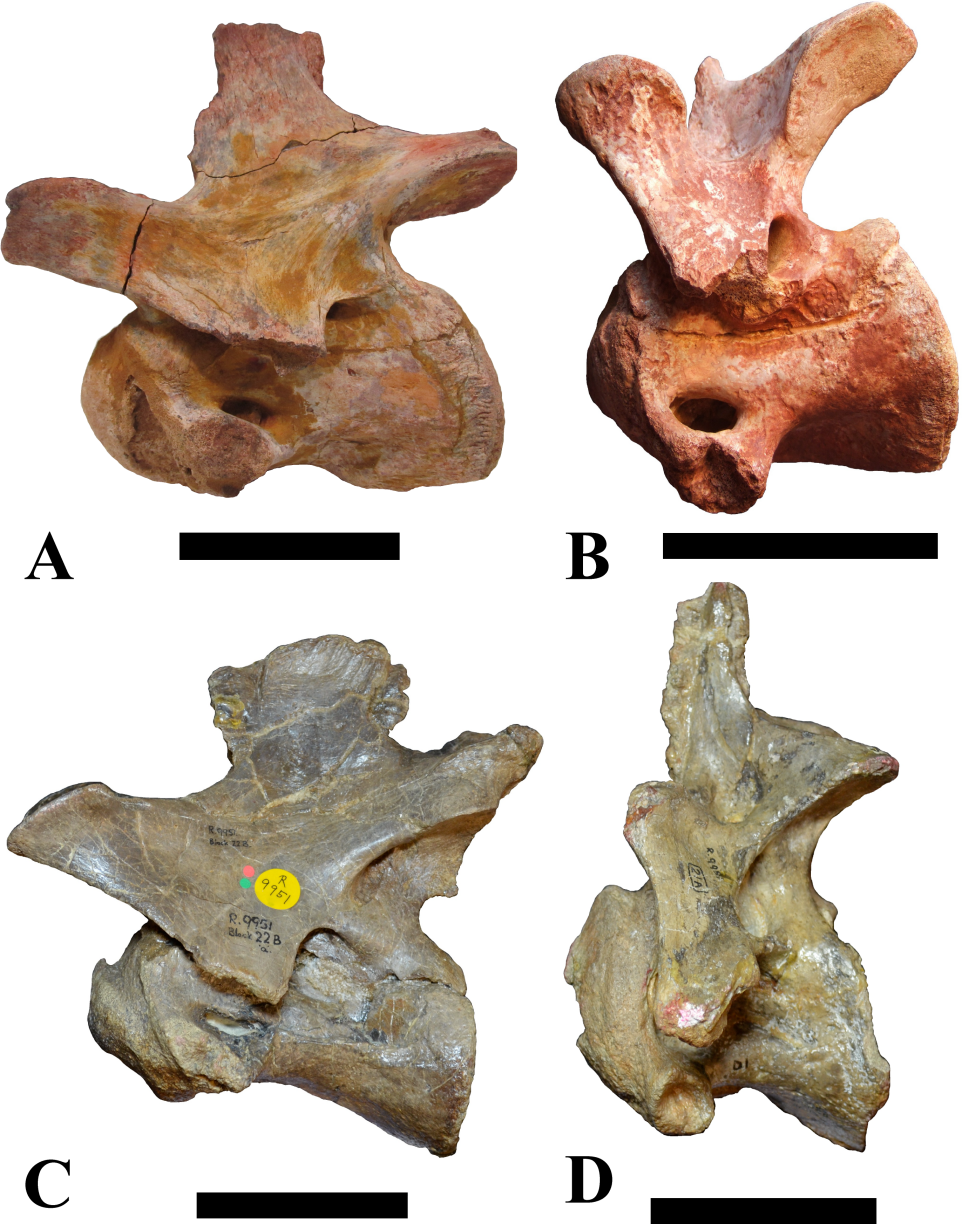
Morphological changes along the cervical vertebral column of *Sigilmassasaurus* and*Baryonyx*. (A) CMN 50791, holotype mid-cervical vertebra (C6) of *Sigilmassasaurus brevicollis* in left lateral view; (B) BSPG 2005 I 56, posterior cervical vertebra (C8) of *Sigilmassasaurus brevicollis* in left lateral view; (C) NHMUK PV R 9951, C6 of *Baryonyx walkeri* in left lateral view; (D) NHMUK PV R 9951, C9 of *Baryonyx walkeri* in left lateral view. Note the extreme morphological changes in centrum length, orientation of prezygapophzses, and shape of the neural spine in both taxa. For discussion of axial placement of *Baryonyx* vertebrae see main text. Scale bars equal 10 cm.

The general proportions of the cervical centra of *Baryonyx, Ichthyovenator, Suchomimus* and *Sigilmassasaurus* are comparable. In all taxa, the centrum is elongate and slightly broader than high in mid-cervicals, and gets progressively shorter in posterior cervicals (see Fig. 26). The width–height ratios of *Baryonyx* do not quite approach those of *Sigilmassasaurus*, and there is no consistent width–height-ratio for *Baryonyx*, because the articular facets become progressively wider posteriorly, but retain the same height (Charig & Milner, 1997). Similarly, *Suchomimus* has width–height ratios below those of *Sigilmassasaurus* (1.2 in the vertebra of MNN GDF500 that we consider to represent the D1), but is similar in respect to the reniform posterior facet. In *Ichthyovenator*, anterior dorsals like D1 have width–height ratios as in *Sigilmassasaurus*.

The mid-cervicals of *Baryonyx, Ichthyovenator* and *Sigilmassasaurus* are strikingly similar. The proposed similarities are exemplified by C6 of *Baryonyx* and *Ichthyovenator*, and CMN 50791, the holotype of *Sp. maroccanus*. Besides the inclination of the condyles of their centra, C6 of *Baryonyx* and *Ichthyovenator*, and CMN 50791 share a distinct rim around the anterior articular facet, intercentral articulations which are transversely broader than high, gently reniform outline of the posterior articular facets, and elongate central pneumatic foramina posterodorsal to the parapophyses. Elongate central pneumatic foramina in mid-cervical vertebrae have been interpreted as an allosauroid character (Carrano, Benson & Sampson, 2012), although they are also present in some megalosauroid taxa, including *Baryonyx* (e.g., C4–6; Charig & Milner, 1997: Fig. 20, p. 33), spinosaurid vertebrae from the Kem Kem compound assemblage (BSPG 2006 I 57), and *Eustreptospondylus* (Sadleir, Barrett & Powell, 2008). Camerate pneumatic organization can be neither confirmed nor rejected for *Baryonyx* or *Ichthyovenator*, as the central pneumatic foramina are closed with matrix, and no CT-data is available for those taxa. However, a mid-cervical vertebra of an indeterminate spinosaurid that is not referable to *Sigilmassasaurus* (BSPG 2006 I 57, see above) has large single foramina on either side of the centrum. BSPG 2006 I 57 lacks some parts of its left anterolateral centrum. Because of this breakage, it is apparent, that this particular spinosaurid is another member of the group that bears large pneumatic cavities of the camerate type in cervical centra, although these camerae are more subdivided than the single large camera of *Sigilmassasaurus*. *Suchomimus* also displays the camerate type of pneumatization: the large foramen dorsal to each parapophysis leads into a single, undivided, large cavity in its cervical vertebrae (posterior cervical, MNN GDF500). Although there is no information about the internal tissue for all currently recognized spinosaurid taxa, the available evidence suggests that the entire Spinosauridae had camerate pneumatization.

**Figure 27:**
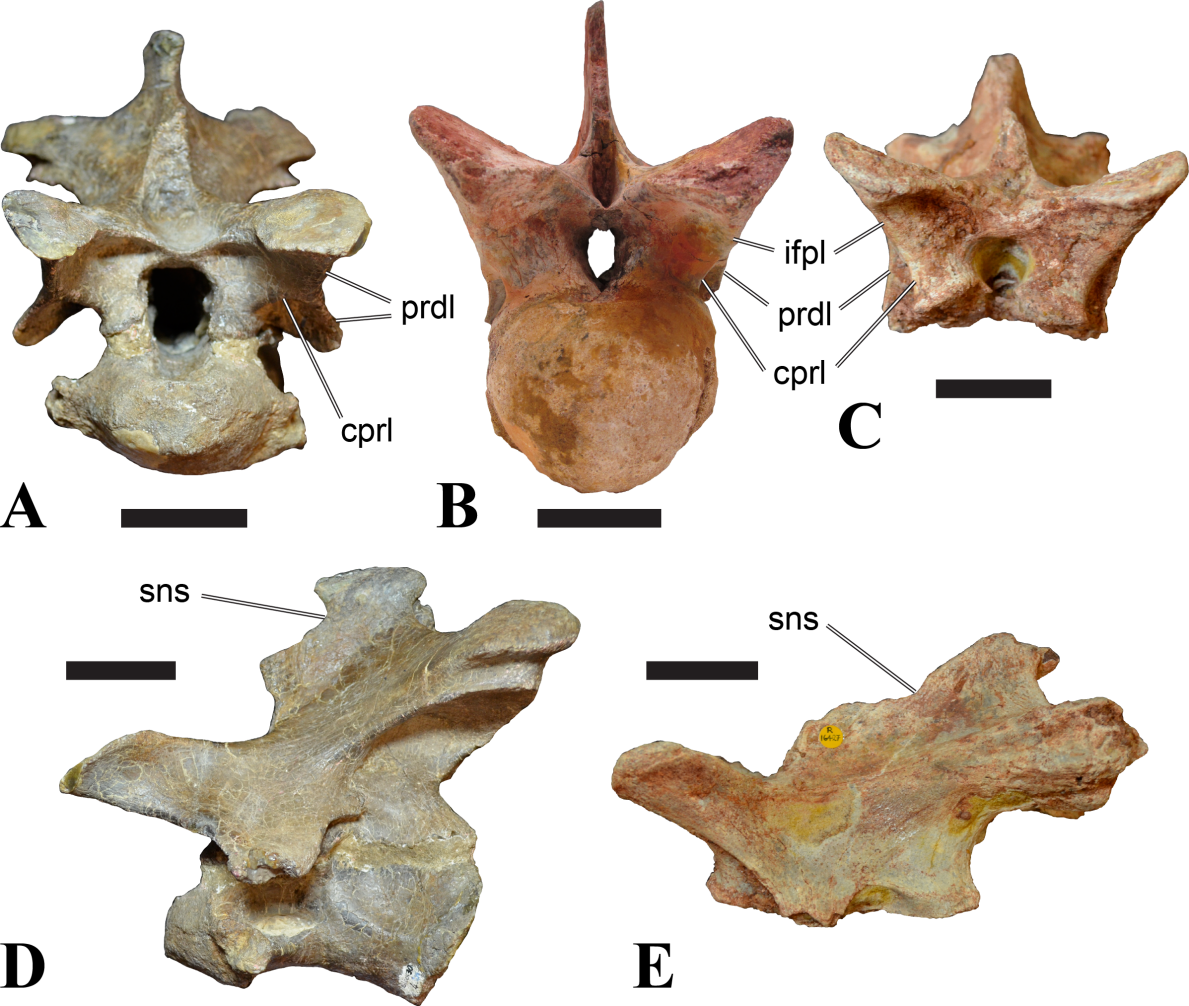
Comparison between mid-cervical vertebrae of *Baryonyx* and *Sigilmassasaurus*. (A) Ce4 of *Baryonyx* in anterior view; (B) CMN 50791, *Sigilmassasaurus* mid-cervical vertebra (Ce6-7); (C) NHMUK PV 16427 *Sigilmassasaurus* mid-cervical (?Ce4) in anterior view; (D) Ce4 of *Baryonyx* in left lateral view; (E) NHMUK PV 17427 *Sigilmassasaurus* in left lateral view. Abbreviations: cprl, centroprezygapophyseal lamina; ifpl, infraprezygapophyseal lamina; prdl, prezygodiapophyseal lamina; sns, stepped neural spine. Scale bars equal 5 cm.

Although *Baryonyx* cervical vertebrae have been described as lacking a ventral keel (Charig & Milner, 1997), the underside of C6 of *Baryonyx* does show a weak ridge. Importantly, the ridge merges posteriorly into a progressively broadening structure, which is slightly elevated and ends in the margin of the ventral part of the posterior cotyle of the centrum. This corresponds to the elevated triangular complex described herein for *Sigilmassasaurus*, although the structure is much less pronounced in *Baryonyx*. In *Ichthyovenator*, the ventral keel is absent, and although a stepped outline of the ventral central margin is seen in the C7 in lateral view, no solid ventral plateau as in *Sigilmassasaurus* is found (see above). This is similar in some vertebrae from the Kem Kem compound assemblage, which also have an albeit smaller stepped outline in lateral view, but lack a convex triangular plateau. Instead, the stepped outline is formed by two stout ventrolateral tubercula, which border a median ventral depression (see above, also see Fig. 20). The mid-cervicals of *Baryonyx* and *Sigilmassasaurus* furthermore share the blade-like, but relatively low neural spines, and the absence of laminae connecting the epipophyses with the prezygapophyses (Fig. 27), which are also absent in *Suchomimus*. The neural spines are higher in *Ichthyovenator*, but also blade-like in mid-cervicals (Fig. 27). Other differences between the taxa include the length of the epipophyses, which are larger in *Baryonyx* and *Ichthyovenator* than in *Sigilmassasaurus*, well-developed centroprezygapophyseal fossae in *Ichthyovenator* and *Baryonyx* (Fig. 27), a relatively prominent transverse ridge between the parapophyses in *Baryonyx*. However, all of these differences are related to diagnostic or at least characteristic features for *Sigilmassasaurus*, which for instance lacks centroprezygapophyseal fossae altogether and differs from most other theropods in having only weakly expressed epipophyses and a rudimentary and stout neural arch lamination. The weak development of epipophyses, although not evident in *Baryonyx* and *Ichthyovenator*, can also be found in *Suchomimus*.

Vertebrae of *Baryonyx* and *Ichthyovenator* with a more posterior axial position are also similar to *Sigilmassasaurus*. BSPG 2006 I 56, which is an important specimen in linking the vertebrae of the *Sp. maroccanus* morphology with the ‘typical’ *Sigilmassasaurus* morphology, shares aspects with posterior cervicals of *Ichthyovenator* and *Baryonyx*, like C9. These vertebrae share a rim around the anterior articular facet, prominent parapophyses in a low-central position and with deeply concave capitular facets (which is not so prominent in *Ichthyovenator*), gently reniform posterior articular facets, strongly curved postzygapophyses, stout transverse processes, and thick ridges running from the neural spine onto the dorsal surface of the postzygapophyses in *Baryonyx* and *Sigilmassasaurus*, whereas *Ichthyovenator* retains epipophyses. Many of those features (e.g., rimmed articular facet; reniform shape of posterior facet; prominent, yet slightly higher positioned parapophyses) are also found in a posterior cervical of *Suchomimus* (MNN GDF500), but incomplete preservation of the specimen prevents comparison of many aspects of the neural arch. The probable D1 of the holotype does, however, show that transverse processes in the cervicodorsal region of *Suchomimus* were also robust, although the lamination is better developed than in *Sigilmassasaurus*. On the ventral side of the centrum, similarities to *Sigilmassasaurus* can be found in spite of the distortion of C9 of *Baryonyx*. Centrally behind the weak transverse ridge of the C9 starts a gently projecting keel. In the center of the underside of the centrum, the keel is abruptly sheared, and its posterior part is right-laterally displaced. From there on, the keel broadens to a posterior triangle, which merges with the posterior articular facet. Like in the mid-cervicals, this triangular structure is less plateau-like in *Baryonyx*. In *Suchomimus* (MNN GDF500), a ventral keel is well developed in a posterior cervical; it has a straight edge, broadens slightly anteriorly, but without developing into a hypapophysis, and it does not broaden posteriorly in any way. The neural spine of BSPG 2006 I 56 is partly broken, but appears to have been spike- or rod-like, and vertically oriented. The spine of the C9 of *Baryonyx* is vertically oriented and rod-like (see Fig. 26). Its anteroposterior length is enlarged by bony extensions with a rugose structure, which seem to be parts of ossified interspinal ligaments. In other, slightly more posterior positioned *Sigilmassasaurus* vertebrae, like CMN 41774 or CMN 41790, the neural spine is developed as a vertical, rod-like projection with a pointed end. The same can be seen in *Ichthyovenator* and *Suchomimus*. Although the described change in neural spine morphology can be observed in many theropods, and is likely to be functionally constrained as the anteroposterior length reduction of the spines allows for dorsoflexion at the beginning of the neck, the spines are more pointed in *Sigilmassasaurus, Ichthyovenator, Suchomimus* and *Baryonyx* than in other theropods, such as *Allosaurus* (DINO 11541, SMA 0005).

The centra of anterior dorsal vertebrae of *Baryonyx, Suchomimus* and *Ichthyovenator* share with *Sigilmassasaurus* strongly developed ventral keels. However, in *Baryonyx*, the ventral keel is straight along its course, while it is ventrally convex in NHMUK PV R 16345 and D1 of *Suchomimus* (MNN GDF500). In the latter taxon, keel morphology changes in following vertebrae, as the probable D2 has a straight keel, and D3/4 has three low and subparallel keels on the ventral side of the centrum. The hypapophyses of posterior cervicals and anterior dorsals show a distinct step anteriorly in *Sigilmassasaurus* and *Baryonyx*, whereas hypopophyses are absent in *Suchomimus*, despite a slight transverse thickening of the keel anteriorly. Rims around the anterior articular facet are strongly developed in *Sigilmassasaurus, Suchomimus* and *Baryonyx*, as well as in *Ichthyovenator*. The anteriormost dorsal vertebrae of *Baryonyx* (D1 and D2) show a reduced convexity of the anterior condyle, and in *Suchomimus*, the reduction of opisthocoely starts with the probably second dorsal vertebra. This is not the case in CMN 41858 and NHMUK PV R 16345, which are anterior dorsal vertebrae of *Sigilmassasaurus*, or in *Ichthyovenator*, which retains a hemispheral anterior condyle just like *Sigilmassasaurus*. The reduction seen in *Baryonyx* is only apparent in tentative more posterior element of *Sigilmassasaurus*, like BSPG 2013 I 95 and CMN 41850. This suggests, that the opisthocoely of presacral vertebrae levels off later in the axial series in *Sigilmassasaurus* than in most theropods, including *Baryonyx*. However, the complete loss of opisthocoely seems to happen relatively far posterior in the vertebral column in all examined spinosaurids. The central pneumatic foramina show a plastic morphology in both *Sigilmassasaurus* and *Baryonyx*; the foramina are large on one side, and much smaller on the other in NHMUK PV R 16345 and D2 of *Baryonyx*.

One of the important similarities between *Sigilmassasaurus*, *Ichthyovenator*, *Suchomimus,* and *Baryonyx* is the development of their transverse processes. The transverse processes of dorsal vertebrae taper distally in most theropods, including *Majungasaurus* (O’Connor, 2007), *Allosaurus* (Madsen, 1976, SMA 0005), or *Neovenator* (Brusatte, Benson & Hutt, 2008), so that the transverse processes are anteroposteriorly more elongate at their medial bases than at the diapophyses. *Baryonyx, Suchomimus, Ichthyovenator* and *Sigilmassasaurus*, in contrast, have distal ends of their transverse processes that are as long anteroposteriorly as the medial base of the processes. Instead of tapering distally, the processes slightly reduce their anteroposterior dimension in the mid-length of the processes, and become broader again toward the diapophyses. This is exemplified by D1 of *Baryonyx* and NHMUK PV R 16345, and best seen in dorsal view. Both specimens have exceptionally stout and elongate transverse processes, but the lamination is more delicate in *Baryonyx, Suchomimus* and *Ichthyovenator*, in which anterior and posterior centrodiapophyseal laminae are well developed, as it is also the case in *Allosaurus* (DINO 11541, SMA 0005; Madsen, 1976). The length of the transverse processes, although extreme in the dorsal vertebrae NHMUK PV R 16345, is also conspicuous for posterior cervical and anterior dorsal vertebrae of *Sigilmassasaurus, Ichthyovenator, Suchomimus,* and *Baryonyx* (e.g., BSPG 2006 I 55; NHMUK PV R 16434; C9 and C10 of *Baryonyx*; D1 *of Ichthyovenator*; D1 of *Suchomimus*). In these vertebrae, the transverse processes are about as long as the width of their respective vertebral centra. In the well-known *Allosaurus*, posterior cervical vertebrae have transverse processes of shorter length (about 65% of the respective centrum width), and they are less thick and stout (e.g., *Allosaurus* vertebrae UMNH VP 8365, 10192; BYU 725/5266; 725/13051; 725/16628).

In anterior dorsal vertebrae, *Baryonyx* and *Suchomimus* retain the rod-like neural spine of posterior cervicals. In more posteriorly positioned dorsals (D10 of *Baryonyx*; unknown positioned vertebra of MNN GDF500; MNN GDF508 (referred to *Suchomimus*; Sereno et al., 1998), the neural spine becomes blade-like, stretches over most of the anteroposterior length of the neural arch, is relatively tall and vertically oriented. In its central part, the neural spine is anteroposteriorly shorter than at its base and at its apex in D10 of *Baryonyx*, while the spine expands continuously in *Suchomimus* (MNN GDF500). Posterior to this position, like in D13 of *Baryonyx*, the spine becomes posteriorly inclined, and the anteroposterior constriction of the central part of the spine is lost. Although the neural spines of the posteriormost *Sigilmassasaurus* vertebrae known to date are only partly preserved, no posterior inclination is evident from the preserved base in CMN 41850, and a weak posterior tilt is evident in BSPG 2013 I 95. Also, the base of the spines of BSPG 2013 I 95 and CMN 41850 are broader than the dorsalmost parts preserved. Therefore, the transition of dorsal neural spine morphology observed in the isolated *Sigilmassasaurus* specimens seems to fit the patterns seen in *Baryonyx*.

**Figure 28:**
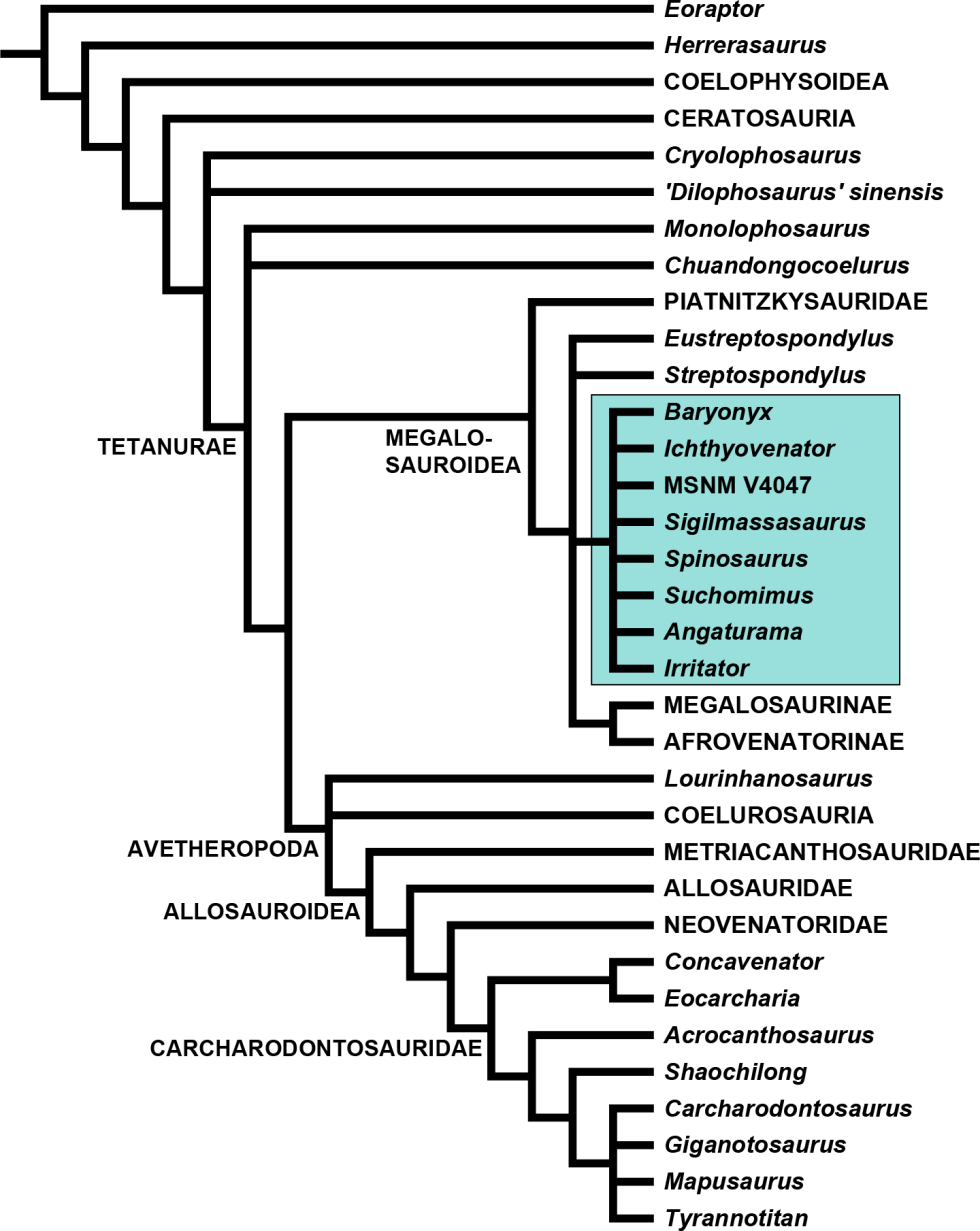
Reduced consensus tree from our phylogenetic analysis after pruning *Xuanhanosaurus* using 3,070 most parsimonious trees. Capitalised terminal clades have been summarised, but are fully shown in a version of this tree in Fig. S6. Note that Sigilmassasaurus is found in a monophyletic Spinosauridae, highlighted by a turquoise box.

In order to further test the phylogenetic affinities of *Sigilmassasaurus*, we performed a cladistics analysis based on a modified version of the Carrano, Benson & Sampson (2012) data matrix (see ‘Materials & Methods,’ as well as Data S1 for details) to evaluate the phylogenetic position of *Sigilmassasaurus*. Our analysis recovers 3.070 most parsimonious trees with a tree length of 1.041 steps from 10.000 replicates (for strict consensus tree, see Fig. S5), using TNT (Goloboff, Farris & Nixon, 2008). The topology of our phylogenetic analysis generally agrees with that of Carrano, Benson & Sampson (2012) with, for instance, a monophyletic Allosauria with the same ingroup relationships as in the tree presented by these authors, or a monophyletic Megalosauria with the same ingroup relationships except for Spinosauridae. In contrast to Carrano, Benson & Sampson (2012), we recover a large polytomy of spinosaurids, which include *Sigilmassasaurus*, *Spinosaurus aegyptiacus* (sensu Stromer, 1915), MSNM V4047, and *Ichthyovenator* in addition to those taxa found in the clade in the earlier analysis. However, the relationships within tetanurans more derived than *Chuandongocoelurus* He, 1984 and *Monolophosaurus* are not resolved in our strict consensus tree, so that we calculated a reduced consensus by pruning *Xuanhanosaurus* Dong, 1984 from the analysis, which was identified as a wildcard taxon. Figure 28 shows a reduced consensus tree of our analysis (see Fig. S6 for ingroup relationships of taxa capitalized in Fig. 28). Features of the topology and differences to the results of Carrano, Benson & Sampson (2012) are briefly discussed in Data S1, and additional trees such as the strict consensus and the ingroup relationships of larger groups generalized in Fig. 28 are also shown there. In both the strict and reduced consensus trees, *Sigilmassasaurus* and *Ichthyovenator* are found as members of a monophyletic Spinosauridae within Megalosauroidea. The Spinosauridae is supported by four unambiguous synapomorphies. Those are (i) the presence of crown striations (character 142:1; present in all spinosaurids preserving teeth, i.e., all but *Sigilmassasaurus*); (ii) anterior carina situated at the base of the crown on maxillary and dentary teeth (character 147:0; present in *Suchomimus*, *Baryonyx*, and *Ichthyovenator*. In *Angaturama*, *Irritator* Martill et al., 1996, *Spinosaurus*, and MSNM V4047, the character definition is not applicable to the morphology of the teeth, see discussion in Data S1); (iii) the presence of a ventral keel in posterior-most cervical and anterior dorsal vertebrae, which forms a straight to slighty convex ventral margin, and has an anterior end that protrudes ventrally from the anterior articular surface, so that it is separated from it by a distinct step (character 181:1; present in all spinosaurids preserving respective axial material); and (iv) the presence of pneumaticity/webbing at base of neural spines in middle to posterior dorsals (character 182:1; present in all spinosaurids preserving respective dorsal vertebrae). As only cervical and anterior dorsal vertebrae are known for *Sigilmassasaurus*, only one unambiguous spinosaur synapomorphy can currently be confirmed for this taxon. The ambiguous synapomorphies that support the Spinosauridae (those synapomorphies that have been recovered in some, but not all MPTs) are: fused premaxillary suture in adults (character 1:1); presence of a mediolateral constriction of the posterior portion of the premaxilla (character 8:1); subnarial formamen developed as an expanded channel (character 10:1); interlocking premaxillary–maxillary articulation (character 11:1); anteroposteriorly long anterior ramus of the maxilla (character 12:2); long an plate-shaped palatal process of the maxilla (character 15:1); absence of a maxillary fenestra (character 26:0); continuous dorsal and ventral portions of the antorbital fossa (character 44:1); lacrimal with angle <75° between anterior and ventral rami (character 49:1); subrectangular shape of the quadrate head in dorsal view (character 83:1); basipterygoid process of basisphenoid located anteroventrally of basal tubera, with a basisphenoid recess opening posteroventrally (character 95:1); splenial foramen developed as a large opening (character 129:1); paradental plates of mandible obscured by medial wall of the dentary, only triangular apices may be visible from medial view (character 139:1); reduced or absent curvature of teeth (character 141:1); circular mid-crown cross-sections of teeth (character 144:1); tapered root-shape of teeth (character 145:1); absence of serrations in maxillary and dentary teeth (character 146:1); absence of serration in premaxillary teeth (character 149:1); six or seven premaxillary teeth (character 150:3); Length-posterior height ratio of mid-cervical centra is 1.75–2.75 (character 179:1); presence of accessory centrodiapophyseal lamina in middle to posterior dorsal vertebrae (character 183:1); pronounced ventral keel in anterior dorsal vertebrae (character 185:1); tall neural spines in dorsal vertebrae, extending twice the height of the centrum (character 194:2). All spinosaurid taxa are found in a polytomy in our analysis, so that the distinction between Baryonychinae and Spinosaurinae is neither supported nor rejected by our analysis. Excluding *Ichthyovenator* from the analysis, however, allows retrieval of the dichotomy between Baryonychinae and Spinosaurinae, with *Sigilmassasaurus* being recovered as a member of Baryonychinae. This group is supported by five unambiguous synapomorphies (3:1; 5:0; 155:1; 177:0, 183:1), of which *Sigilmassasaurus* possesses only one, i.e., neural spines in mid-cervicals anteroposteriorly longer than dorsoventrally high. If *Sigilmassasaurus* is excluded from the analysis, *Suchomimus* and *Baryonyx* are recovered among Baryonychinae, whereas *Ichthyovenator*, *Irritator*, *Angaturama* and *Spinosaurus*, and MSNM V4047 form a polytomy with this clade. Given the morphological similarities between cervical and dorsal material in *Ichthyovenator*, *Sigilmassasaurus* and *Baryonyx* on one hand, but spinosaurine characters displayed by *Ichthyovenator* on the other hand, this indicates that *Ichthyovenator* increases the character conflict within the group. At this point, it is speculative to comment on the ingroup relationships of Spinosauridae, yet the new morphological information gained from the examination of *Ichthyovenator* suggests that the previously found distinction between Baryoninchinae and Spinosaurinae might not be as strongly supported as previously thought. In any case, our phylogenetic analysis strongly supports the hypothesis derived from the comparative anatomical analysis of our study that *Sigilmassasaurus* is a valid taxon of the Spinosauridae.

### Spinosaur diversity and ecology

Spinosauridae is a group of bizarre megalosauroid theropod dinosaurs, which have received much interest due to their extremely large body size (Dal Sasso et al., 2005; Therrien & Henderson, 2007), superficial similarities to crocodilians in terms of the elongated rostrum, conical tooth shape, and suggested semiaquatic lifestyle (e.g., Taquet, 1984; Buffetaut, 1989; Charig & Milner, 1986; Charig & Milner, 1997; Sereno et al., 1998; Taquet & Russell, 1998; Sues et al., 2002; Milner, 2003; Dal Sasso et al., 2005; Rayfield, 2007; Allain et al., 2012; Ibrahim et al., 2014a), as well as their tendency to develop elongated neural spines in at least part of their axial series (Stromer, 1915; Allain et al., 2012).

Currently, five spinosaurid taxa are considered valid, including *Baryonyx*, *Ichthyovenator*, *Suchomimus*, *Irritator*, and *Spinosaurus* (Carrano, Benson & Sampson, 2012; Allain et al., 2012), although the validity of *Suchomimus* has been doubted by some authors (Sues et al., 2002). Other taxa have been proposed, including *Siamosaurus* Buffetaut & Ingavat, 1986, *Ostafrikasaurus* Buffetaut 2013, *Suchosaurus* Owen, 1842, *Cristatusaurus* (Taquet & Russell, 1998), *Angaturama* and *Oxalaia* Kellner et al., 2011, but are based on very fragmentary remains (Owen, 1840-; Sauvage, 1897-; Buffetaut & Ingavat, 1986; Kellner & Campos, 1996; Taquet & Russell, 1998; Kellner et al., 2011; Buffetaut, 2012), the diagnostic validity of which is questionable (see e.g., Sues et al., 2002; Mateus et al., 2011; Rauhut, 2011). The most common spinosaurid occurences are by far isolated teeth (e.g., Buffetaut, 2008; Canudo et al., 2008; Hone, Xu & Wang, 2010). Despite the fact that spinosaurid teeth are highly diagnostic (Hendrickx & Mateus, 2014), the identifications of many isolated teeth have been questioned due to the danger of confusion with crocodilian teeth (Holtz, 1998) or ceratosaurid theropods (Fowler, 2007). Of the five diagnostic spinosaurid taxa mentioned above, *Baryonyx* was recovered from the oldest strata, the Barremian Wessex Formation of England (Charig & Milner, 1997), and *Spinosaurus* from the youngest strata, the Cenomanian deposits of Egypt (Stromer, 1915). The temporal range, as well as the spatial range of spinosaurs is significantly increased when more ambiguous identifications are included. Oldest possible spinosaurid occurences include the isolated tooth described as *Ostafrikasaurus* from the Late Jurassic of Tendaguru (Buffetaut, 2008; Buffetaut, 2012 though see Rauhut, 2011) and isolated teeth showing spinosaurid characteristics from the late Middle Jurassic of Niger (Serrano–Martínez et al., 2015), and youngest have been reported from the Santonian of China (Hone, Xu & Wang, 2010). Another possible spinosaur is *Chilantaisaurus* Hu, 1964, the systematic position of which has been a matter of debate (Rauhut, 2003; Benson & Xu, 2008; Allain et al., 2012). Spinosaur remains have been confirmed from northern and western Africa, Europe, Asia, and South America (e.g., Stromer, 1915; Taquet, 1984; Kellner & Campos, 1996; Martill et al., 1996; Russell, 1996; Charig & Milner, 1997; Sereno et al., 1998; Taquet & Russell, 1998; Dal Sasso et al., 2005; Mateus et al., 2011; Allain et al., 2012). Tentative spinosaur material is known from Australia (Barrett et al., 2011; Benson et al., 2012b, though see Novas et al., 2013, for a different opinion). Allain et al. (2012) consider an isolated enlarged manual ungual phalanx of the first digit, originally referred to *Torvosaurus* (Galton & Jensen, 1979), as evidence for the presence of spinosaurs in North America, but this should be seen as tentative, pending more complete material from this continent.

Spinosaur diversity is highest during the late Early and the early Late Cretaceous, when several spinosaur taxa lived contemporaneously, although usually spatially separated from one another. However, if *Sigilmassasaurus brevicollis* is indeed different from *Spinosaurus*, at least two spinosaurid taxa are known from the Baharyia–Oasis (*Spinosaurus aegyptiacus* and *Sigilmassasaurus* sp.) and also from the Kem Kem compound assemblage. Recent studies suggest the presence of at least two spinosaurid taxa from these strata based on tooth morphology (Richter, Mudroch & Buckley, 2013) and the presence of two distinct spinosaurid quadrates (Hendrickx, 2006; C Hendrickx, pers. comm. to SWE, OWMR, and RA, 2013). Here, we have presented evidence from vertebral anatomy that also corroborates this hypothesis. Although the stratigraphic position of many fossils from the Kem Kem compound assemblage is not precisely known and the temporal range covered by the respective deposits does not ultimatively necessitate co-occurrence of individual specimens (Cavin et al., 2010), the abundance and diversity of large bodied theropod dinosaurs from the Cenomanian of Northern Africa is conspicuous, yet not unique (compare with theropod faunas of the Campanian of eastern Asia, or the Late Jurassic Morrison fauna in northern America (Hone et al., 2011)). *Spinosaurus aegyptiacus*, *Sigilmassasaurus brevicollis*, *Bahariyasaurus ingens*, *Sauroniops pachytholus*, and *Carcharodontosaurus saharicus* all represent very large theropods, and remains of large abelisaurs and other theropods like *Deltadromeus agilis* (Sereno et al., 1996) suggest a high number of top predators. Direct competition could perhaps have been avoided by niche partitioning, for instance by different dietary and/or environmental preferences (see Fanti et al., 2014). Thus, the hypothesis of a predator-dominated ecosystem, derived from field occurrence data by Läng et al. (2013) is also supported by the taxonomic diversity of theropod dinosaurs from the ‘Kem Kem beds’ and the Baharyia Oasis.

Evidence for at least a partial picscivorous diet for spinosaurs comes from comparative tooth morphology (see review of Bertin, 2010), gut contents (Charig & Milner, 1997), isotope studies on spinosaur teeth (Amiot et al., 2010), and biomechanical analyses (Rayfield, 2007; Cuff & Rayfield, 2013). In *Sigilmassasaurus*, intense rugose bone surface structures can be found on the underside of the vertebrae, primarily on the anteroventral part of the centrum between the parapophyses and on the ventral side of the periphery of the posterior articular facet. These structures tend to be stronger in mid-cervicals, but also appear in more posterior positioned elements. The rugose pattern of ventral aspects of cervical centra can also be observed in other theropod dinosaurs, like *Allosaurus* (SMA 0005), but the intensity of the pattern is extreme in *Sigilmassasaurus*. Although such rugosities have not been observed in *Baryonyx*, they were present on the original *Spinosaurus aegyptiacus* material (Stromer, 1915).

Intense rugosities are usually osteological correlates for the origin or insertion of relatively strong musculature (Snively & Russell, 2007). Muscles on the ventral aspect of cervical centra in extant crocodylians and birds are associated with ventroflexion (Snively & Russell, 2007; Tsuihiji, 2007). Although this needs further testing from a biomechanical study, intense ventroflexor musculature might be advantageous for quickly snapping at fish and might thus be indicative of a piscivorous lifestyle of *Sigilmassasaurus*, as suggested for other spinosaurids.

## Conclusions

Vertebrae of *Spinosaurus maroccanus* and *Sigilmassasaurus brevicollis* represent elements from different axial positions of the same taxon, and the two taxa can therefore be synonymized under *Sigilmassasaurus brevicollis*. Although the mid-cervical and anterior dorsal vertebrae of this taxon seem to differ substantially in their morphology, observed changes along the axial series are shown to be similar to those of other tetanuran dinosaurs, including the spinosaurids *Baryonyx* and *Ichthyovenator*. Newly described vertebral material shows intermediate features between *Spinosaurus maroccanus* and *Sigilmassasaurus* and thus further supports this hypothesis. *Sigilmassasaurus brevicollis* shows several autapomorphies and a suite of unusual features otherwise known only in *Ichthyovenator*. It is retained herein as a valid taxon, and a synonymy with *Spinosaurus aegyptiacus* is rejected. A phylogenetic analysis recovers *Sigilmassasaurus* as a spinosaur. This further increases the known diversity of spinosaurs, and suggests that the Kem Kem assemblage of Morocco yields at least two taxa of spinosaurs.

## Supplemental Information

10.7717/peerj.1323/supp-1 Data S1 Supporting phylogenetic information Character descriptions, extended phylogenetic results and discussion, and character codings used for this study.

10.7717/peerj.1323/supp-2 Data S2 Phylogenetic matrix Matrix used for phylogenetic analysis in this study.

## Institutional Abbreviations

AMNH: American Museum of Natural History, New York, NY, USA
BMMS: Bürgermeister Müller Museum Solnhofen, Solnhofen, Germany
BSPG: Bayerische Staatssammlung für Paläontologie und Geologie, Munich, Germany
BYU: Brigham Young University, Provo, UT, USA
CMN: Canadian Museum of Nature, Ottawa, Canada
DINO: Dinosaur National Monument, CO-UT, USA
FASC: Faculté des Sciences Aïn Chock (University of Casablanca), Casablanca, Morocco
IVPP: Institute of Vertebrate Paleontology and Paleoanthropology, Beijing, China
MCF: Museo Carmen Funes, Paleontología de Vertebrados, Plaza Huincul, Neuquén, Argentina
MCNA: Museo de Ciencias Naturales y Antropológicas (JC Moyano) de Mendoza, Mendoza, Argentina
MDS: Dinosaur Museum, Savannakhet, Laos
MIWG: Museum of Isle of Wight Geology (now Dinosaur Isle, Isle of Wight Museum Service), UK
MNN: Musée National du Niger, Niamey, Niger
MOR: Museum of the Rockies, Bozeman, MT, USA
MPEF-PV: Museo Paleontológico ‘Egidio Feruglio’, colección Paleontología Vertebrados, Trelew, Provincia de Chubut, Argentina
NHMUK: Natural History Museum, London, UK
MSNM: Museo di Storia Naturale di Milano, Milan, Italy
P.P.: Museo di Storia delle Scienze Biomediche dell’Università degli Studi “G. d’Annunzio,” Chieti, Italy
PVL: Paleontología Vertebrados, Fundación Miguel Lillo, Tucumán, Argentina
OMNH: Oklahoma Museum of Natural History (formerly MUO, Museum of the University of Oklahoma), Norman, OK, USA
SGM: Ministerie de l’Energie et des Mines, Rabat, Morocco
SMA: Sauriermuseum Aathal, Aathal, Switzerland
ROM: Royal Ontario Museum, Toronto, Canada
UA: Université d’Antananarivo, Antananarivo, Madagascar
UMNH: Natural History Museum of Utah (formerly Utah Museum of Natural History), Salt Lake City, UT, USA
USNM: United States National Museum, Smithsonian Institution, Washington, D.C., USA

## Additional Information and Declarations

### Data Availability

The following information was supplied regarding data availability: The DICOM data of the scans is deposited at Figshare: BSPG 2006 I 54, 10.6084/m9.figshare.1471654; BSPG 2011 I 115, 10.6084/m9.figshare.1471659.
