# Supplementary material for: A reappraisal of the morphology and systematic position of the theropod dinosaur Sigilmassasaurus from the “middle” Cretaceous of Morocco
Source: PeerJ. 2015 Oct 20;3:e1323. doi: 10.7717/peerj.1323 (PMC4614847; doi:10.7717/peerj.1323)
Supplement: Data S1 — Character descriptions, extended phylogenetic results and discussion, and character codings used for this study. [file peerj-03-1323-s001.docx]

**Phylogenetic analysis**

**Character Descriptions**

The phylogenetic analysis presented in this study is based on a modified version of the matrix published by Carrano, Benson and Sampson (2012). Because our aim was not a comprehensive phylogenetic study or evaluation of the matrix, we only checked and modified characters on cervical and dorsal vertebrae in order to score *Sigilmassasaurus* for a phylogenetic analysis. Accordingly, the characters 1- 155 of Carrano, Benson and Sampson (2012), as well as characters 196 – 351 were left unchanged. Cervical and dorsal vertebral characters as used in this study, with remarks on changes relative to the definitions applied by Carrano, Benson and Sampson (2012), are listed in the following. In addition to scoring *Sigilmassasaurus* for all possible characters, we included *Ichthyovenator*. Codings based on the specimen MSNM V4047 were removed from the *Spinosaurus aegyptiacus* hypodigm, and were scored as an individual OTU. Also, codings for all theropods in the matrix were checked and/or adapted for characters 156 – 195. Note that *Sigilmassasaurus* was removed from the hypodigm of *Carcharodontosaurus*, and that vertebral codings of the latter are based on the description by Stromer (1931). Carrano and colleagues coded material for which character definitions were inadequate with artificially high character states (such as “7”, “8”, and “9”) instead of using question marks, in order to indicate that the character cannot be coded because of ambiguity rather than because the respective material is absent in the character (pers. comm. Roger Benson, 2015). We changed these numbers to question marks in our matrix, as they are recognized as autapomorphies and increase the tree length.

*Characters 1-155*: As in Carrano, Benson and Sampson (2012).

*Character 156:* ***Cervical vertebrae****, anterior face of anterior elements: flat (0), convex (1).*

Remarks: The definition for character 156 was changed from the “Presacral vertebrae” to “Cervical vertebrae”, as in tetanurans only cervical and anterior-most dorsal vertebrae have convex anterior faces of the centra, and posterior dorsals have a flat or gently concave anterior surface.

*Character 157:* ***Presacral vertebrae****, pleurocoel posterior to parapophysis (anterior pleurocoel) in anterior elements: absent (0), present (1).*

Remarks: As in Carrano, Benson and Sampson (2012).

*Character 158:* ***Presacral vertebrae****, posterior pleurocoel in anterior elements: absent (0), present (1).*

Remarks: As in Carrano, Benson and Sampson (2012).

*Character 159****: Presacral vertebrae****, extent of anterior pleurocoel: anterior dorsals only (0), to sacrum (1).*

Remarks: In the definition of the character by Carrano, Benson and Sampson (2012), character state 0 was “to D4” instead of “anterior dorsals only”. Because in isolated material definite numerical designations are difficult, but anterior dorsal vertebrae are recognizable (e.g. by parapophyses not entirely on neural arch), we changed the character to be able to score isolated material, as such of *Sigilmassasaurus*.

*Character 160:* ***Vertebrae****, internal structure of pneumatic centra: absent, ‘pleurocoels’ if present, form fossae, not foramina (0),camerate (1), camellate (2).*

Remarks: As in Carrano, Benson and Sampson (2012).

*Character 161:* ***Atlas****, length of epipophyses: moderate (0), elongate (1).*

Remarks: As in Carrano, Benson and Sampson (2012).

*Character 162:* ***Axis****, spinous process shape: dorsal end expanded transversely (0), tapers mediolaterally (1).*

Remarks: As in Carrano, Benson and Sampson (2012).

*Character 163:* ***Axis****, orientation of intercentrum ventral surface: horizontal or slightly anteroventral (0), tilted anterodorsally (1).*

Remarks: As in Carrano, Benson and Sampson (2012).

*Character 164:* ***Axis****, length of epipophyses: moderate (0), long (1), short (2).*

Remarks: As in Carrano, Benson and Sampson (2012).

*Character 165:* ***Axis****, morphology of spinopostzygapophyseal lamina: broad, well-developed (0), invaginated (1).*

Remarks: As in Carrano, Benson and Sampson (2012).

*Character 166:* ***Axis****, development of parapophyses: moderate/large (0), reduced/absent (1).*

Remarks: As in Carrano, Benson and Sampson (2012).

*Character 167:* ***Axis****, development of diapophyses: moderate (0), reduced or absent (1).*

Remarks: As in Carrano, Benson and Sampson (2012).

*Character 168:* ***Axis****, pleurocoels: absent (0), present (1).*

Remarks: As in Carrano, Benson and Sampson (2012).

*Character 169:* ***Cervical vertebrae****, morphology of anterior pleurocoel: single opening (0), two openings oriented anteroventral-posterodorsal or very plastic morphology (1).*

Remarks: As in Carrano, Benson and Sampson (2012).

*Character 170:* ***Cervical vertebrae****, middle, shape of anterior pleurocoel: round (0), anteroposteriorly elongate (1).*

Remarks: As in Carrano, Benson and Sampson (2012).

*Character 171:* ***Cervical vertebrae****, anterior, ventral keel: present (0), absent or weak ridge (1).*

Remarks: As in Carrano, Benson and Sampson (2012).

*Character 172:* ***Cervical vertebrae****, anterior, demarcation of dorsal surface of neural arch from diapophyseal surface: gently sloping (0), ridge (prominent prezygapophyseal-epipophyseal lamina) (1).*

Remarks: As in Carrano, Benson and Sampson (2012).

*Character 173:* ***Cervical vertebrae****, articular surface of prezygapophyses: planar (0), flexed (1).*

Remarks: Character number 173 of the Carrano, Benson and Sampson (2012) matrix was deleted, so that the new character 173 is identical to character 174 of Carrano, Benson and Sampson (2012).

*Character 174:* ***Cervical vertebrae****, perimeter of anterior articular surface: not rimmed by a flattened peripheral band (0), flat, forming a distinct rim (1).*

Remarks: Character 175 of Carrano, Benson and Sampson (2012).

*Character 175****: Cervical vertebrae****, anterior, transverse distance between prezygapophyses relative to width of neural canal: < (0), >, prezygapophyses situated lateral to neural canal (1).*

Remarks: Character 176 of Carrano, Benson and Sampson (2012).

*Character 176:* ***Cervical vertebrae****, anterior, morphology of epipophyses: low, wider than high, posteriorly pointed (0), transversely narrow, high (1), high, robust (2).*

Remarks: Character states for this character vary slightly from the definitions of character 177 of Carrano, Benson and Sampson (2012).


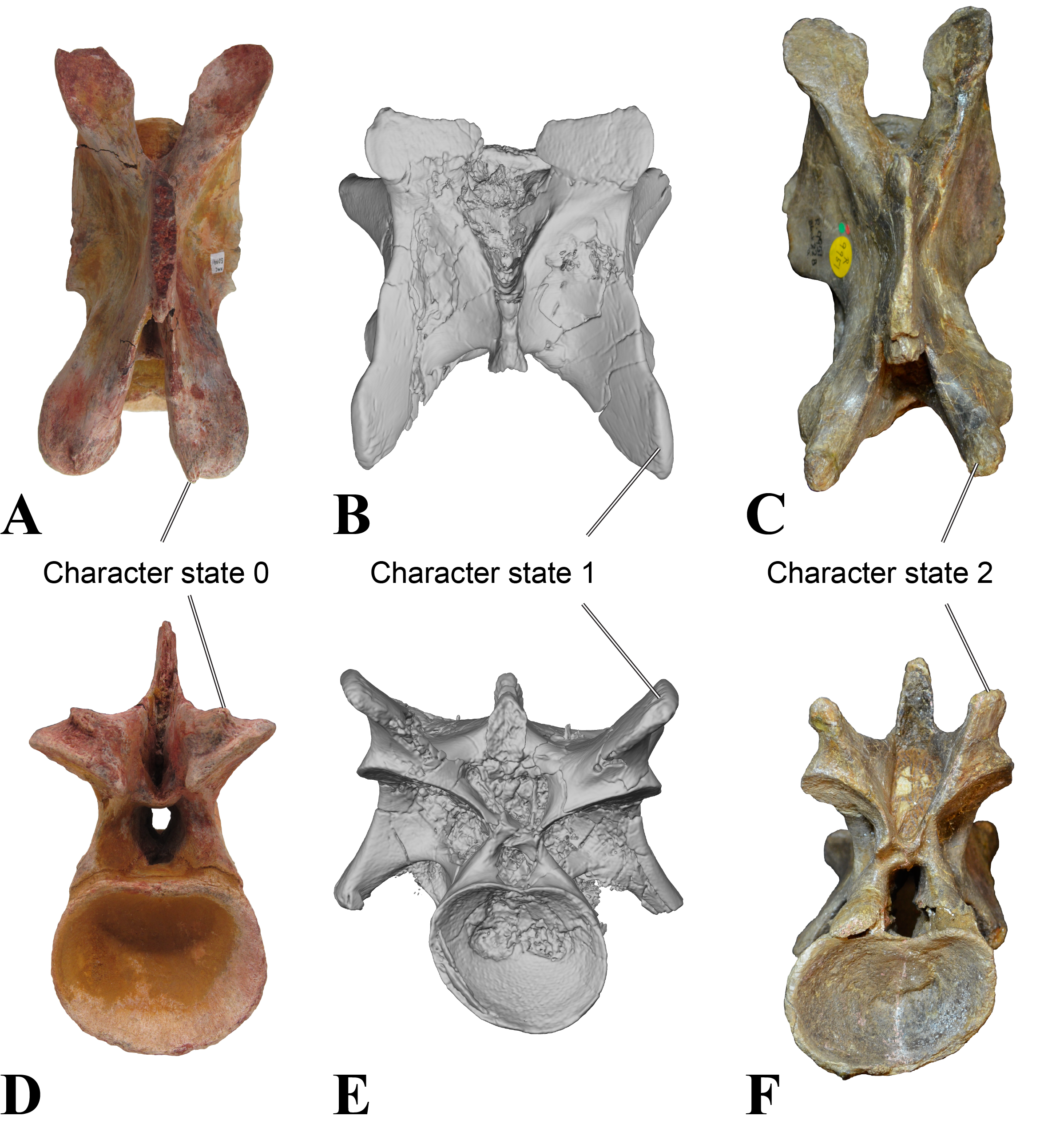


Suppl. Fig. 1. Character 176, morphology of epipophyses. A, D, C6 of *Sigilmassasaurus brevicollis*; B, E, C5 of *Majungasaurus crenatissimus*; C, F, C6 of *Baryonyx walkeri*; A – C, dorsal view; D – E, posterior view. Note that specimens are scaled to approximately same size for comparisons. B & E from O´Connor 2007, used with permission.

*Character 177: Neural spine of* ***mid-cervical vertebrae****: anteroposteriorly longer than dorsoventrally high (0), higher than long (1).*

Remarks: The character definition was changed from the according character (178) of Carrano, Benson and Sampson (2012), and the neural spine length is set in relation to its height rather than to dimensions of the centrum. The character is limited to mid-cervical vertebrae, which have sheet-like neural spines.


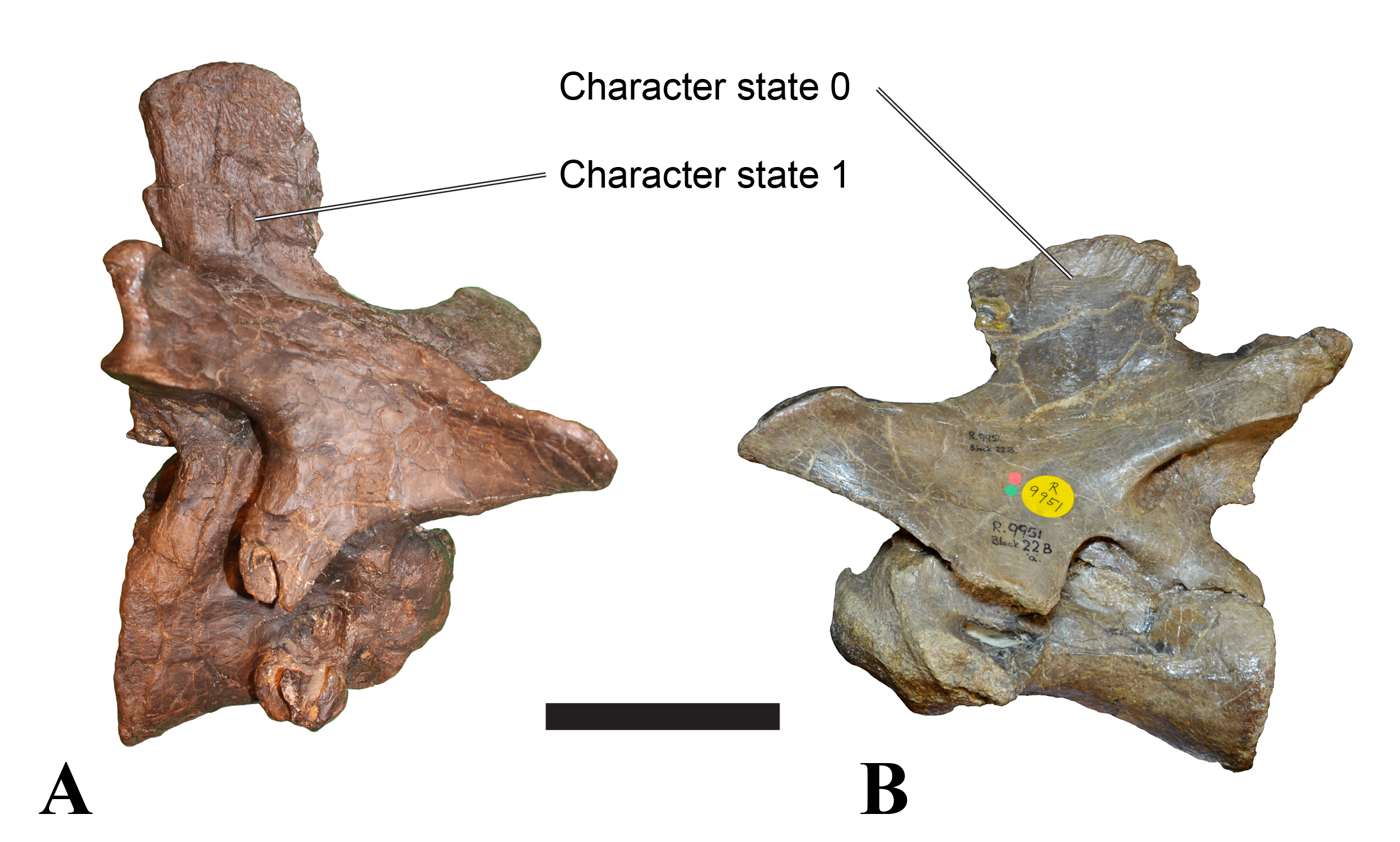


Suppl. Fig. 2. Character 177, neural spine of mid-cervical vertebrae. A, right lateral view of C6 of *Allosaurus “jimmadsoni”*; B, left lateral view of C6 of *Baryonyx walkeri*. Scale bar equals 100 mm.

*Character 178:* ***Cervical vertebrae****, longest post-axial elements: first five (0), last five (1).*

Remarks: Character 179 of Carrano, Benson and Sampson (2012).

*Character 179: Length/posterior height ratio of* ***mid-cervical centra****: less than 1.75 (0), 1.75-2.75 (1), more than 2.75 (2).*

Remarks: The character state definitions for this character were changed from the according character (180) of Carrano, Benson and Sampson (2012), and an additional range for ratio values is introduced in order to accommodate differential ranges among theropods.

*Character 180: Height of* ***anterior dorsal*** *neural spines (as measured from the dorsal margin of the postzygapophysis): less (0) or more (1) than 1.25 times the height of the neural arch (as measured from the dorsal rim of the centrum to the dorsal margin of the postzygapophysis).*

Remarks: This character has no equivalent in the matrix of Carrano, Benson and Sampson (2012), and is newly added to the character list.


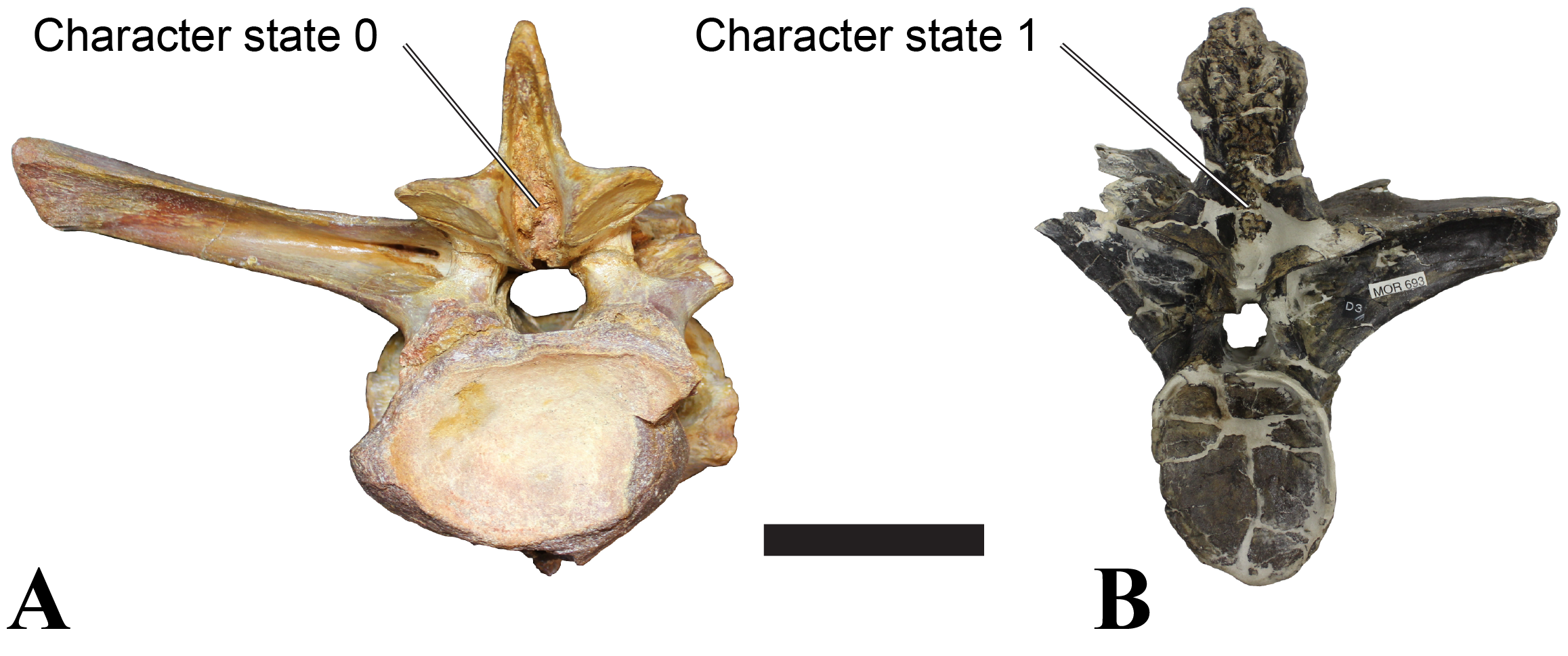


Suppl. Fig. 3. Character 180, height of neural spines in anterior dorsals. A, posterior view of anterior dorsal of *Sigilmassasaurus brevicollis*; B, posterior view of D2 of *Allosaurus “jimmadsoni”*. Scale bar equals 100 mm.

*Character 181: Ventral keel in* ***posterior-most cervicals and anterior-most dorsals****: ventrally concave, a rounded hypapophysis might be present anteriorly (0), forming a straight to slightly convex ventral margin, anterior end of keel protrudes ventrally from the anterior articular surface and is separated from the latter by a distinct step (1).*

Remarks: This character has no equivalent in the matrix of Carrano, Benson and Sampson (2012), and is newly added to the character list.


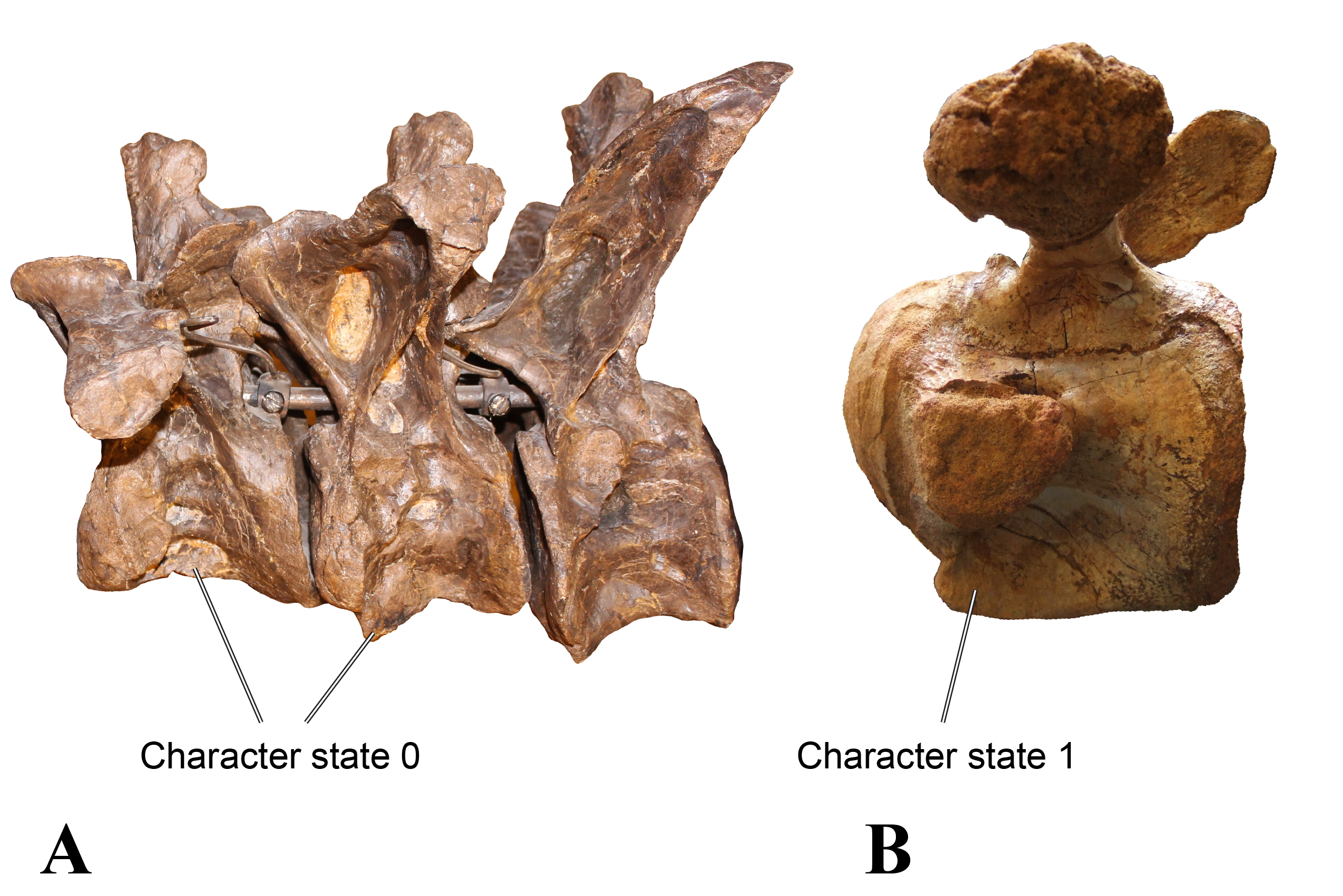


Suppl. Fig. 4. Character 181, ventral keel and hypapophysis in posterior-most cervical and anterior-most dorsal vertebrae. A, left lateral view of D1-D3 of *Allosaurus “jimmadsoni”*; B, left lateroventral view of anterior dorsal of *Sigilmassasaurus brevicollis*. Specimens are scaled to similar size for comparisons.

*Character 182:* ***Dorsal vertebrae****, pneumaticity/webbing at base of neural spines in middle to posterior dorsals: absent (0), present (1).*

Remarks: Character 181 of Carrano, Benson and Sampson (2012), but character definition is limited to middle to posterior dorsals, because even theropods exhibiting webbing (e.g. *Baryonyx*) lack this feature in anterior dorsals, and therefore the absence of webbing cannot be implied in taxa only preserving anterior dorsals.

*Character 183: Middle to posterior* ***dorsal vertebrae****, accessory centrodiapophyseal lamina: absent (0), present (1).*

Remarks: Character 182 of Carrano, Benson and Sampson (2012), but character definition is limited to middle to posterior dorsals, because even theropods exhibiting accessory centrodiapophyseal laminae (e.g. *Baryonyx*) lack this feature in anterior dorsals, and therefore the absence of the feature cannot be implied in taxa only preserving anterior dorsals.

*Character 184:* ***Dorsal vertebrae****, size of infraprezygapophyseal fossa: small (0), expanded (1).*

Remarks: Character 183 of Carrano, Benson and Sampson (2012).

*Character 185:* ***Dorsal vertebrae****, anterior, ventral keel: absent or developed as a weak ridge (0), pronounced, around 1/3 the height of centrum and inset from lateral surfaces (1).*

Remarks: Character 184 of Carrano, Benson and Sampson (2012).

*Character 186:* ***Dorsal vertebrae****, anterior, size of pneumatic foramen in centrum: small (0); enlarged (1).*

Remarks: Character 185 of Carrano, Benson and Sampson (2012).

*Character 187:* ***Dorsal vertebrae****, elevation of parapophyses: slightly elevated from centrum (0), project far laterally, more than half the diapophyseal length (1).*

Remarks: Character 186 of Carrano, Benson and Sampson (2012).

*Character 188:* ***Dorsal vertebrae****, orientation of hyposphene laminae: diverge ventrolaterally (0), parallel and sheet-like (1).*

Remarks: Character 187 of Carrano, Benson and Sampson (2012).

*Character 189:* ***Dorsal vertebrae****, position of parapophyses in posteriormost elements: on the same level as transverse process (0); distinctly below transverse process (1).*

Remarks: Character 188 of Carrano, Benson and Sampson (2012).

*Character 190:* ***Dorsal vertebrae****, distinct step-like ridge lateral to hyposphene, running posterodorsally from dorsal border of neural canal to posterior edge of postzygapophyses: absent (0); present (1); ridge present and is developed into a prominent lamina that bisects the infrapostzygapophyseal fossa in posterior dorsal vertebrae (2).*

Remarks: Character 189 of Carrano, Benson and Sampson (2012).

*Character 191:* ***Dorsal vertebrae****, middle and posterior, postzygapophyses with tab-like lateral extensions of articular facets: absent (0); present (1).*

Remarks: Character 190 of Carrano, Benson and Sampson (2012).

*Character 192:* ***Dorsal vertebrae****, morphology of neural spines: transversely compressed sheets (0), transversely broad anteriorly and posteriorly, central regions of lateral surface embayed by deep vertical troughs (1).*

Remarks: Character 191 of Carrano, Benson and Sampson (2012).

*Character 193:* ***Dorsal vertebrae****, posterior, inclination of neural spines: vertical or posterior (0), anterior (1).*

Remarks: Character 192 of Carrano, Benson and Sampson (2012).

*Character 194:* ***Dorsal vertebrae****, height of neural spines relative to centrum height: low, ≤ 1.3x (0), moderate, 1.4-1.8x (1); tall, ≥ 2.0x (2).*

Remarks: Character 193 of Carrano, Benson and Sampson (2012).

*Character 195: Middle to posterior* ***dorsal vertebrae****, centrum length relative to height: more than 2 (0), less than 2 (1).*

Remarks: Character 195 of Carrano, Benson and Sampson (2012), but character definition extended to entire dorsal series.

*Characters 196-351*: As in Carrano, Benson and Sampson (2012).

**Results**

Our strict consensus tree has the same topology as the tree found in Carrano, Benson and Sampson (2012) in all groups basal in relation to Orionides, but has little resolution in Tetanurans more derived than *Monolophosaurus* and *Chuangdongcoelurus* (Fig. S5). A monophyletic Megalosauria, Allosauria, Piatnitzkysauridae, and Coelurosauria are found in a polytomy with Methriacanthosaurids and *Lourinhanosaurus*, the latter of which is found outside of Coelurosauria.


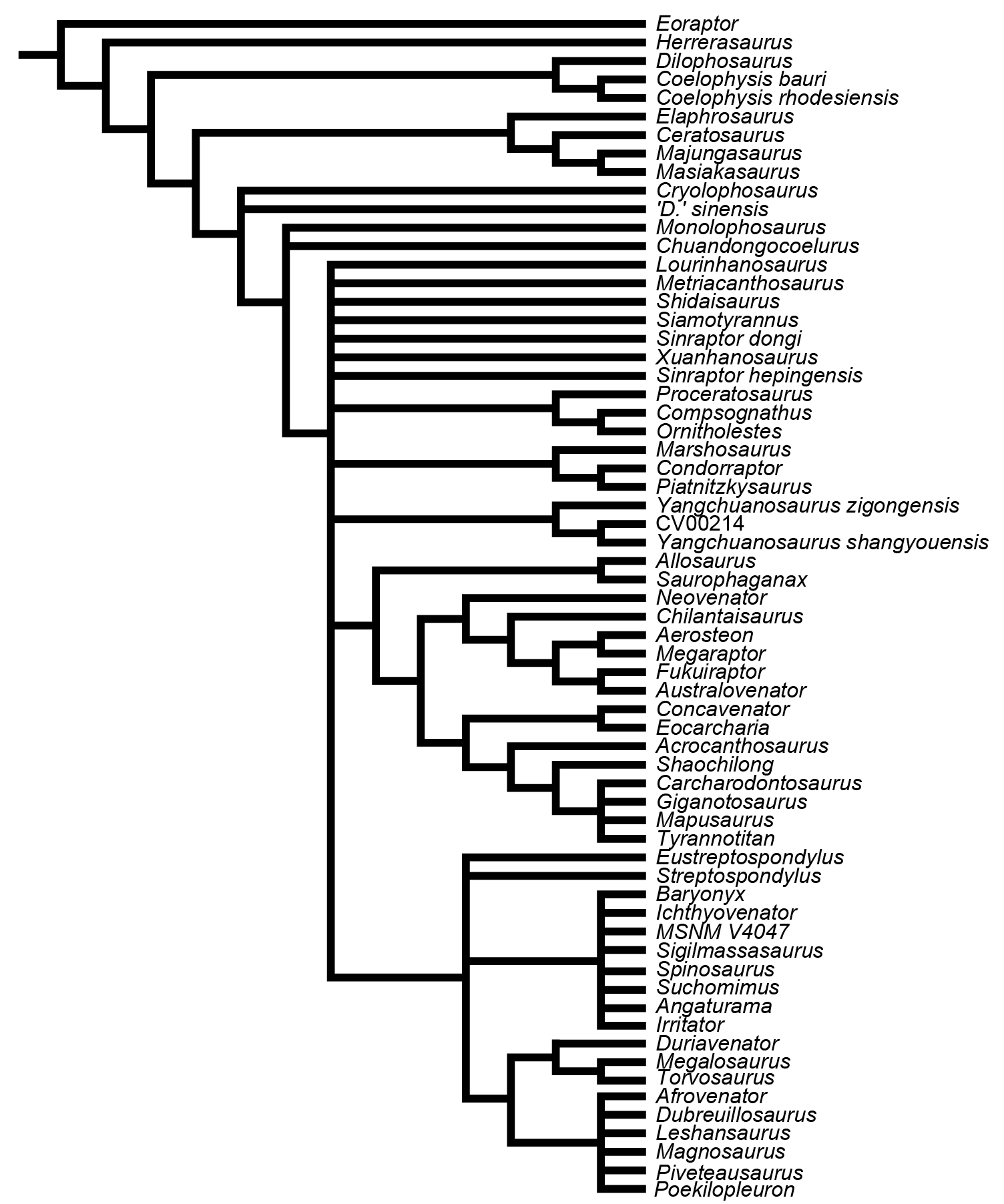


Suppl. Fig. 5. Strict consensus tree, calculated from 3.070 MPTs retained from a heuristic search with 10.000 replicates. Tree length is 1.041.

By pruning *Xuanhanosaurus* from the tree, more resolution is achieved (Fig. S6). The reduced consensus tree recovers Piatnitzkysauridae as a sister clade to Megalosauria, forming a monophyletic Megalosauroidea with the same topology as in Carrano, Benson and Sampson (2012). Megalosauroidea forms the sister clade to Avetheropoda, which comprises allosauroids, coelurosaurians, and *Lourinhanosaurus*. However, our tree rocovers *Lourinhanosaurus* in a polytomy with Allosauroidea and Coelurosauria. As our analysis aims to find the phylogenetic position of *Sigilmassasaurus*, we will not further comment on this topology and the position of *Lourinhanosaurus* in respect to Coelurosauria. Within Avetheropoda, Allosauroidea is found with the same topology as in Carrano, Benson and Sampson (2012) in our analysis.

**
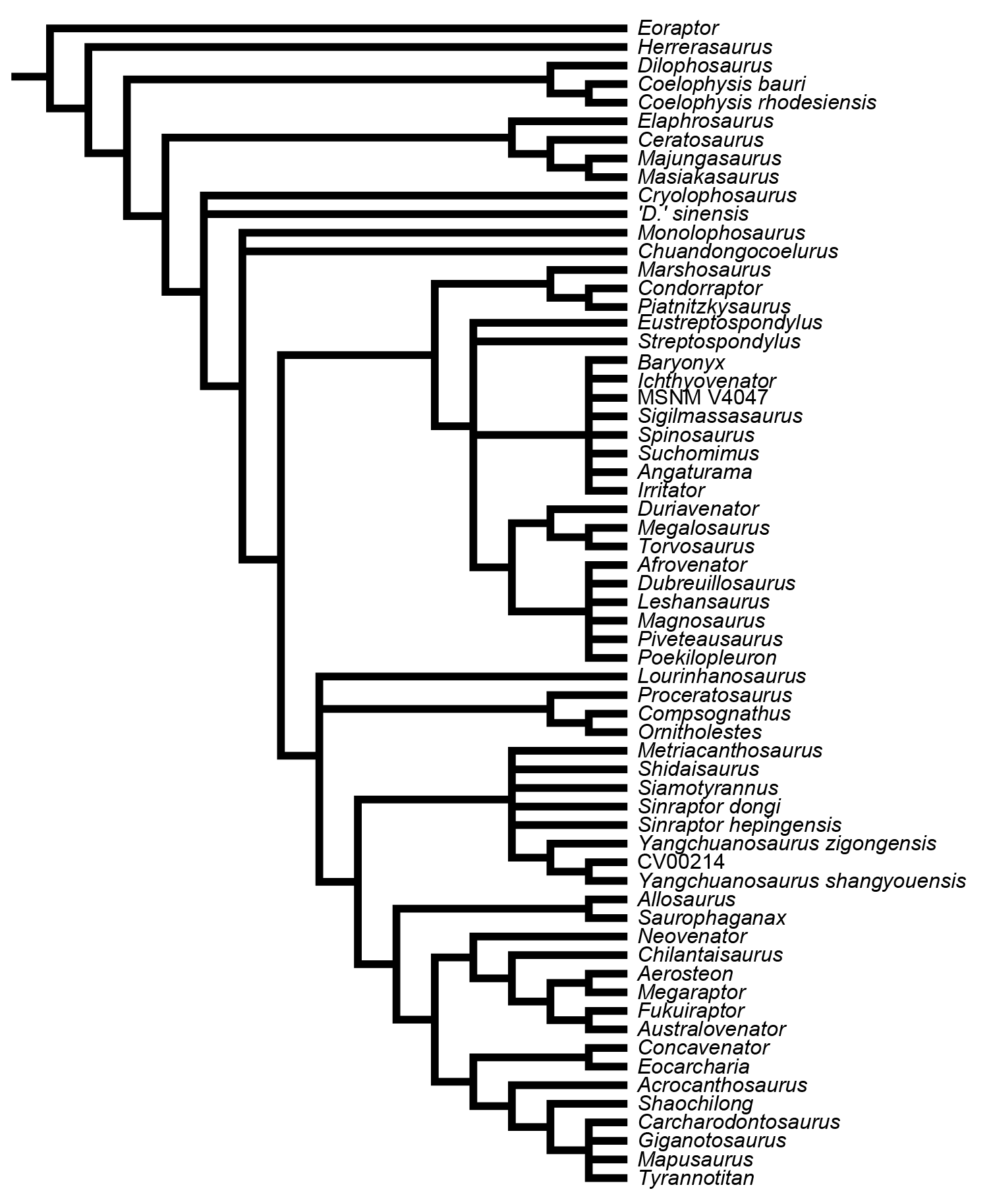
**

Suppl. Fig. 6. Reduced consensus tree with *Xuanhanosaurus* pruned from the analysis.

**Taxonomic and nomenclatural remarks to the phylogeny**

In the phylogeny presented here, *Szechuanosaurus zigongensis* is referred to as *Yangshuanosaurus zigongensis*, as ‘*Szechuanosaurus’* probably represents a *nomen dubium*, as its type species has no distinctive features and consist of teeth only (see Carrano, Benson and Sampson, 2012), and is herein recovered with other members of the genus (following and as in Carrano, Benson and Sampson, 2012).

*Yanchuanosaurus hepingensis* is referred to as *Sinraptor hepingenis*. Although our analysis does not have enough resolution among Metriacanthosaurids to support or reject the hypothesis that ‘*Y*.’ *hepingensis* is closer related to *Sinraptor* that to other species of *Yangchuanosaurus*, the hypothesized relationship of Carrano, Benson and Sampson (2012) and Currie and Zhao (1993) is followed here, pending further resolution within the Metriacanthosaurids.

*Y. magnus* is referred to as *Y. shangyouensis*, following the proposed (not formally established) synonymy by Carrano, Benson and Sampson (2012).

*Dilophosaurus* *sinensis* is referred to as ‘*D*.’ *sinensis*, as we recover the taxon as further derived than *Dilophosaurus* and other membes of the Coelophysoidea, and therefore agree with Carrano, Benson and Sampson (2012) that ‘*D*.’ *sinensis* pertains to a different genus. Some workers use the name *Sinosaurus triassicus* for *D. sinensis* (Xing et al., 2013), but this is not practiced here, pending a formal synonymization of *D. sinensis* with *S. triassicus*.

**Character codings**

*Acrocanthosaurus*

020?1100?0000001111000000211111100010?000000211101211100020212011?1121111020110?02??11012100000100110100012011111?111002100101020010?11111?000100010000010011012?00110011?0000121101{01}00100011001121110?0021000000101?1?110110120012001110?001011110000113110111111000????010?10??1110110201201?120?201111000220111011110000110010302?1010?1110?1??01210111001?1

*Aerosteon*

????????????????????????????????????????????????????????01?000?111??????????10000200???????????????????????????????????????????????????????????????????????110121???????11?000120?011001000101101011?????2?110??0?????????0101100121????????????????????????????????01011010010221110110201201?120??????????????????????????1001?402????01112021110????????????

*Afrovenator*

?????????0010101100000100120011000?????0?000201001?011001011000?????????00?001111001?????????????????????????????????????????????????????0000000001?????10?11001?0?0110001100112?10??0001?001?00001???????100000???1?01010??????????0111?????00?0?????0?31001111?100001?10?01101011?011?2012?1?00?001011100011101100001100001000020?11010110???111011?011100??1

*Allosaurus*

00101{01}000001000101000000021111110001101100002{01}2{01}0010110000000101111100011020000002001101100011010010010201201111111112000100110201111111110000100011020010011001100110010{01}100012110100010000100010111000011?0000000101?11011012001200111010110000100011131001111110000111010010211110110201201?1100200111000{12}10111011110000110010202210101111021110121011100101

*Angaturama*

11???201?1121?11???0?????????????0?????????????????????????????????????????????????????????????????????????????????????1?0??????????????????110111?01311???????????????????????????????????????????????????????????????????????????????????????????????????????????????????????????????????????????????????????????????????????????????????????????????????????

*Australovenator*

?????????????????????????????????????????????????????????????????????????????????????????????????????????????????????????00001???????????100001000110????????????????????????????????????????????????????????????0????????????????????????????00010101????01?1111??101??????????????????????????????????????2201210111100011100114022101011121211101211????010?

*Baryonyx*

111?0201011210110?0000???02???1??01????0?001202011?0??????0??0?1111100??????0?11011010001011?01??010210?011??????????001101010?{12}101???1??0100101100013001011100{12}?1001001011001120110111010001100011010?0???000?00??1??1?00??0?1?0100011111100010011010????????????10?0?11010?101?1110110{01}0?2?1?0????0011000????1????00110100????????110?0?????????????0????????

*Carcharodontosaurus*

??????0??000000111100001001101110101111000?02?1?0??111100??21211211?211????0????????110121?0??0100??01121121???1???????2?10??????????????1000020000?????10?110???????0?10?0?0?1???0????????????????????????100?0???1???1??????????????????????????????????????????????????1??1???????11?????0?????0??11?????22??1101111??01?100???????010??????????????????????

*Ceratosaurus*

0210100010000000000000000010100010120?00000020200110000000100001111110001010010010001010100000000110100001110000?100010000001??1000000100100010000010100100011021?0100010000000111010000001010000110211??10000210?0100001101011110100100110000????0??1??1?01?00010?010?11100?0000111011110110100001{01}0001001100110{01}001102100010100200211101001021??1?0001??10???

*Chilantaisaurus*

????????????????????????????????????????????????????????????????????????????????????????????????????????????????????????????????????????????????????????????????????????????????????????????????????????????????????????????????????0110110000????????????????????11??????10????2110????????????????????????22??111???1000?1100??40221????1?????????2101??0????

Chuandongocoelurus

??????????????????????????????????????????????????????????????????????????????????????????????????????????????????????????????????????????????????????????????0????????????????????????????????????????????0????????????????????????????????????????????????????????00???0011101011???1?0?????0?????????????00?101000100000?1000040?2121011???21110?100??100???

*'Coelophysis*_*bauri'*

00101211000010????0?011010100?0000000?0000101??0000000000000000?0?00000110110100000?0000000?0?2??1????0??1?0?0?0?00??00110?0?0000?00?0000000010000?0000000001100?10001100??0000001200000000?000000001000000000011?1100?01?000001002000001000000?0{01}?00101110100000000100100011000011011110100?10000001000010000110{01}00?00?000?01000100002100000010111100011000000

*'Coelophysis*_*rhodesiensis'*

00???211000010???00?01101100?00000000?020010100001{01}00000000?000100000000?0110?0000000000000000200100?000?10000000001000100000000000000000100000000100000000011011?00011001000000012000000000?000000010000?0000?11?1100001?000001002000001000?0010{01}000110110100000000100100011000111011110000?1000?011100010000111{01}001102000?0100?100002100000010111100011010000

*Compsognathus*

00???100000100????0?010002100?1??0000?0000000??00000?000?00?00????1?00????????0??0????0??00?????0??0?0??????1?1????1?0000?00??0??100??10100?0000?0??0000100?100{12}???????101?01??0012?000?000????000??1??0??000?0?00?1?0?0111?011100?0?????0???0???0??01?1310011????0?000??010?11??110011?20?201?011?11?10??002???111?????????1?0???????01011???????01??0?11?0101

*Concavenator*

?????0???????0??????001002??0?1?00?1111?10102010000?110??10??2????1?????????????????????????????????????????????????????????????????????????0020?0??????{01}0??100????????????????{12}?1????00????1???021?1?1??{01}100?000????1?111110?10?1??0?0??10??0?1010001??????????????0?0?0?10?11??111011020????????02011???1?22??1100???????????????2??0?0?1?1?????0???0?1?00???

*Condorraptor*

????????????????????????????????????????????????????????????????????????????????????????????????????????????????????????????????????????????0010001????????01001????????0000001???1?00001000?2001?11101001?0002???01??101???????????????????????????????????????????0?1??01?01010????????0????0???00001100????????0??01000001?00??01????????????????1?01???0???

*Cryolophosaurus*

???????????????????00?????????1??00?1001?01010?000?0111??0?00{01}0?1?10000?10??000000??10001000001??1?0010????0??????1??0????????????10?000000000?0?01??????0?01000????????0??0000?????0000??001000??10?0?????000200????0?01??????????????????????0?10001??????????????00???00???0?????011??0????0???0?0??10011001?0?00110100000000?2??????01001011??0????????????

CV00214

???????????????????????????????????????????????????????????????????????????????????????????????????????????????????????????????????????????????????????????1100{12}?0110001??100012110??001000???000{12}1??0?????00?1?????????????????????011010?0?00101000??0??000???????000?0010?10?????0110{12}0?{12}0??00?0?001???1?{12}1?10111?110?00???0???0???0?0?1???????01???????0?0?

*'D*_*sinensis'*

?0?0?000?0010???????000202??0?1??0??101300100??000?00?00?01?000???1??0?????1?0?????????????????????????????????????????00??0?00???1?????????0??0?0??????10?0100{12}?1?00??0001000???10??00000100?00001??000?1000020??01?010??010001?000111111000000010001???1010111?000000?0101?0000111011?{01}002010000?200010111001110000101000?1000021001010?0????0???110011100??1

*Dilophosaurus*

01?1121100001000000{01}001000100000000?100200100??00?200100001?0001001000?1101101000000000?000000200101000001??0??0?????201100010000?1000000{01}1000000010000010101101??00011101100001011000000000000001101000010000201??10000100000010010010010000001010001?111011000100010010001100001100110{01}000?10000010001?00100110{01}000000000001000201002100001010110100011000?0?

*Dubreuillosaurus*

01???100?0010101?000001001{12}0001??0??0??0??002??001?0????1011?001011?000000??????????10??2011100?0??110010??0??????010?0110111??201???????0000000?0100000100?1001??????????????????????00??0?1?????1???????1??000?10????0110?????0???????????????????????????????????????????????????????????????????????????????11????0?0???????????1?01????????????????1??????

*Duriavenator*

00???100??0101011000?0?0?120?1???0?????????????????????????????????????????????????????????????????????????????????????110101????????????0000010?0??0?00100????????????????????????????????????????????????????????????????????????????????????????????????????????????????????????????????????????????????????????????????????????????????????????????????????

*Elaphrosaurus*

???????????????????????????????????????????????????????????????????????????????????????????????????????????????????????????????????????????????????????????0110{12}????????0110000001200000001?000000002111?0000011??010???????0???11?01100100001000???????????????????000111000000?111111100?{12}??????0?0?00001100110?00110??00010000200??21?0??????111?001???1????

*Eocarcharia*

?????????00000011100101002111111?0??????????????????????01?00201111???1??????????????????????????????????????????????????????????????????1000010?01?????10?????????????????????????????????????????????????????????????????????????????????????????????????????????????????????????????????????????????????????????????????????????????????????????????????????

*Eoraptor*

00010000?00000??????0101000?0?0010000?0000100??0001?0000001000000?0000000000?10?00??0?001000?0000?0??????0??00?0?101000000?0?0000?00?0000?0?00?000100000000000?0??????????000000001000000?0?0000000000000?0000001?0010000?000001000000001000000000?00100000100000000000010000000?00000000000??000?0000000?00000?0000?000?000000000????0000000000000000000000000

*Eustreptospondylus*

?1???20000011?01100000010120?1???0???????100200001??????10?10001?1??00000???0111100010??1001000100?11000012??????????00110111????????????00000?0?010000010011001???0110101100112?10?000001001100011?100??1?0000????1????????111?0???0111210?00???????????????????????00110101100011?011300?2?1?000001010100?1111110000110000100002011101?110102111??1001??000??

*Fukuiraptor*

???????????????????0?0?0????????????????????????????????????????????????????????????????????????????????????????????????0????????????????1000010?0110????00110?{12}????????1????????????????0????????1??????????????????????????????12001111101?001?1010??????????????10?????10????????????????????????????????21012101?1100011??01??02????01112121??012?1???0????

*Giganotosaurus*

02???000??0?00?11?100001001101???10?1110100021110????????2021211??11211??0??1?00020?110121???00100?0011211?1??????111??201010?????1?1111110?0020000??00010011012?01?0??11100?012??0?00?100?11001?2?2?????2?00000???1??0?10??0???01?0????????????????????????????????00?11010010211110110201201?120020111100022011101??1?0???1001?30221010?10???????????????????

*Herrerasaurus*

00001000000000????0?0001000?0?0?00000?0000100??001201000000000000?00000100000000020?000010?00000?10??00000000?000000?10000?0?0000?000010010000?0?0100000000000?001000010??000000001?00000?0?100000?0000000000000??001??00?1?011?0??000001000000100000101000100000000000010000000?00000000000?0?001000000?00000??0000100000000000000???0100001000000000000000000

*Ichthyovenator*

????????????????????????????????????????????????????????????????????????????????????????????????????????????????????????????????????????????1120?10????????1100??00110010010011211{01}011111100110002101??00000{01}0011???????????????????????????????????????????????????0000100001021111121220020110000001001110???????????????????????????????????????????????????

*Irritator*

?????2????12?0???????01100200?100010??0000010?201110010000??00011?11000????0?0???11?10??10?10010001021000?101?10?????0????????????10?11?0???1101?1???????1???????????????????????????????????????????????????????????????????????????????????????????????????????????????????????????????????????????????????????1?????????????????????????????????????????????

*Leshansaurus*

???????????1?1?1?001??1?0{01}???1????????????????????????????????????10000?????????????000000010021001??00????0??????????????????????????????00???0????????{01}0?1100?1?0010??0?1000121?0?0000??0????00001100??????????????????????????????????????????????1????????????0?0??????0110?0111011?{12}0????????0??0??????{01}????100001???1?10???201110?0??????????????????0???

*Lourinhanosaurus*

???????????????????????????????????????????????????????????????????????????????????????????????????????????????????????????????????????????????????????????1100?????????0?????1???0??00?000?0?00??1?1?0??1100000?????0?111??????????????????????????????????????????00?01010?1011111011?2?????1???0?001100??11011101?0110?????01?20?2101???????????????????????

*Magnosaurus*

?????????????????????????????????0?????????????????????????????????????????????????????????????????????????????????????110?11????????????0000000?0????????0???0???????????????????????????????0????????????00????????????????????????????????????????????????????????0???01??1?????????????????0??0?????????1???????0?110?001000?20?????0?11???????????????????

*Majungasaurus*

0210100010000001000000010010000011120?10020000?101200000020202012?11110110110000100010100?00000010?01101?1010000010101100101001100100010010100000001000010001102100110010?110011-100000000101000001021?1111001201?01000011?1011?10?011002000010??????0????????????0??0011100000001111111??????0???1??1??????0????100110?100010100200211111111021111?0001??1101?

*Mapusaurus*

??????0???00000111100001001101?101011110100021110??1111102?2121121????????{02}01?000?0??????????????????????????????????00201010?01001??????1000020000?0???10011012???01???1??000110???0001??01?0010212?????210000?????????1?11012?0????1???10?10???????0????1????????000???0?0?102?1110110{12}?????????0?011?100022??1101111?00?1100??30221010110??2111012101??0010?

*Marshosaurus*

000??0000000010010001102011001?000010?000??????????0?1001?10000????1000000?10?0?01?0100020000001001?0?00?1?0???????????10001??0???1?011??00100100010000010011001?011111000100012110?000000001200111?1??????????????10???????0?1????????????1????????????????????????00?110100101011?0110010201?0000?00111000?????1??????????????????21???????0?????????????????

*Masiakasaurus*

0?????0???00100100000100001000???0?????????10???????????0??20101?10?????????0?001??00????000?0001??00???010??????????11101000011?0??00?000100000000???0020001102?0011001001100110?2000000010100000002011110001011?0100001?01011111001100100001????????????????????001001?1000000011?111111120100001001001??10011010011021000101002002?1111112021111?0011??1101?

*Megalosaurus*

?????????0000?01101100?00110?1?000?????????????????1?111???????????????????????????????????????????????????????????????000101?????0?011000010010?01??00?100???01????????0?????12??????0?1????1000211100001?0000???010?????01110?000001112110?000010000??????????????00101010110101110113?00{12}??????0?10??1?0011101100001?000010000211??0?0?1????1????1001??00???

*Megaraptor*

???????????????????????????????????????????????????????????????????????????????????????????????????????????????????????????????????????????????????????????11012????????11?100120?0??????????1?????????????110?0???????1?111011?0121????????????0101011?2?01011111110??????????????????????2????????????????????????????????????????????????????????2?11???????

*Metriacanthosaurus*

????????????????????????????????????????????????????????????????????????????????????????????????????????????????????????????????????????????????????????????1001??????????????1??????001??00?0001211?????1?000?0???1????1???????????????????????????????????????????00??10100101111?011??012?1?0?00?101?01?111??110?11110001??0???11???????????????????????????

*Monolophosaurus*

001?1000?00100??????000002000?100011101300100??0012{01}11000110010?11110001?021000000??1000100000010?????0????01?1??????001000010020100?0???00?0010?0??0000101110010?0010010?10001{02}11010000000?1000011?10?0?1?000?00?010???????????????????????????????????????????????0011000101010111011?00?{12}0100???0001000?0???????????????????????????????????????????????????

MSNM V4047

110?1201011210????0??0000020??1??0?????0????????????????????????????????????????????????????????????????????????????????????????????????????110111?01311?1?????????????????????????????????????????????????????????????????????????????????????????????????????????????????????????????????????????????????????????????????????????????????????????????????????

*Neovenator*

00???1000001000101010000021001???10110110????????????????????????????????????????????????????????????????????1?????????110000????????????1000010001?020010011012?1?????111100012?10?0001000110101010?0???2100001???1?10?10?1011?0120????????????????????????????????01?11010010221110110201201?12000001?1011020?1101111000111001130221010?10???1????210111?010?

*Ornitholestes*

000??000?00000??????000002100?1?00001000001010?0012?11000001000?1??10001?020?00??0??1000?00???0????0??0??????????????00000?0??0?0?0?0100111?000000??00001001100{12}??????????1010120?1?000?000100000010100??1?00010??010??11??????????00101100000000???01??11??111??1??00?0001011121111011020????????010010000021??211??0100001???????2??0?0???????????2001??0????

*Piatnitzkysaurus*

??????????000?01000010020110?1???0????????????????????????????????1???0?????????????10?0100000010?1101000?10???????????2010?1????????????0010010?01?????00001001?00111100?1000121111{01}00010001200?111100001?000?0??010?????01011?112001111101100??100?1??????????????001?10100101011?011?0002?10000000011000011?11100001000001000021121010?10???1????1001??0????

*Piveteausaurus*

????????????????????????????????????????????????????????????????011?000?????????????1000?0?1002100??1002?110???????????????????????????????????????????????????????????????????????????????????????????????????????????????????????????????????????????????????????????????????????????????????????????????????????????????????????????????????????????????????

*Poekilopleuron*

??????????????????????????????????????????????????????????????????????????????????????????????????????????????????????????????????????????????????????????????????????????????????????????????????????????100?0??1?????111??????????011??10110001000001????0?11????0????????????????????????????????????????????????????????10???2????0?01111021?10????????000?

*Proceratosaurus*

000??000?00100??????000002100?1??0????????0???????201100000????????????1??20??0??0????????????????????0??????????????00{02}00?0??0???00??1?1?110000?0??0000000????????????????????????????????????????????????????????????????????????????????????????????????????????????????????????????????????????????????????????????????????????????????????????????????????

*'S_zigongensis'*

????????????????????????????????????????????????????????????????????????????????????????????????????????????????????????????????????????????00?0??1?????00?1100{12}?????0010??0001{12}?1?00001000?1?00?01?1??0??000?100?????????01011?0???010021001000010001??2?00111111??00001010?10??1110110101{12}0?100?020011011111?1??0??110?000?00??201{12}10?0??010?????????????????

*Saurophaganax*

?????????????????????????????????????????????????????????0??????????????????????0??????????????????????????????????????????????????????????????????????????1100?1??????????0001???0??00?0??0??????1????????00??0????????10??????????011?0101100???????????????????00001?1010010?????????20?{12}01?1?00??01?????210?110??01???011001?20??????????????????00????????

*Shaochilong*

??????????000??11?10100100110111?00???1???????????????????????????1?011?????0?00020011???1?0000100?0011??120?????????????????????????????1?????????????????????????????????????????????????????????????????????????????????????????????????????????????????????????????????????????????????????????????????????????????????????????????????????????????????????

*Shidaisaurus*

???????????????????????????????????????????????????????????????0??11000?????????????110?0??????????0????????????????????????????????????????0??0?00???????????0??0110000??????1??????001?00????01210100001?00???????0???????????????????????????????????????????????00??0?1??1???111011020?{12}0??00??20??0??0????????????????????????????????????????????????????

*Siamotyrannus*

?????????????????????????????????????????????????????????????????????????????????????????????????????????????????????????????????????????????????????????????0{12}??????????????????????????????0???101000?0?000????010???????????????????????????????????????????????002010100102?11101122012110000??101101?????????????????????????????????????????????????????

*Sigilmassasaurus*

???????????????????????????????????????????????????????????????????????????????????????????????????????????????????????????????????????????????????????????110?1????????011001100?101??010?????0???????????????????????????????????????????????????????????????????????????????????????????????????????????????????????????????????????????????????????????????

*'Sinraptor_dongi'*

00001000000000000000010002111111000110100000211000011100010001001?110000112001000200110110001001001001120100111?11111000000110020011111000000010?0?1000010011001101100010010001{12}1101000110001100121010?0?1?000?000010???1?01011100??????????????????????2???????11?00020101001010111011220121110000210110101110111011110000110000211210101101021110121011100101

*Spinosaurus*

?????????????????????????????????????????????????????????????????????????????????????????????????????????????????????00110?0??011????????010110111????????01100{12}??????????100??21?1??100?00???0?0210?00????000?0??0????????????????????????????????????????????????????????????????????????????????????????????????????????????????????????????????????????????

*Streptospondylus*

???????????????????????????????????????????????????????????????????????????????????????????????????????????????????????????????????????????????????????????1100{12}????????0??001?????00000010?1?0???10???????000?0??0?0?????????????????????????????????????????????????????????????????????0??1?000????????0??????????01000?01????2???????110102111?????????????

*Suchomimus*

111102010112101?0?00000100200?1??0?????0??????????????????????????1???????????110110???????????????????????????????????110??????1????????0100101100013001011100{12}???????10??00112????111010001100021?10?0???00000???1001?0001011?0100011111100010011010????????????100011?010?1000111011000?{12}?0?00?000011?00?11??110100110100100??40211010?102021??0?1??????????

*Torvosaurus*

00???100?0010??110110001012000??00????00?101200001?011111011000?{01}??????????001111?01???????????????????????????????????010?0?????????????00000100010010010011011010210010010011211000000110011000111100001110000??010?101001110000000111211000000100?0{12}???001101101000001010110101110113000{12}?1000002001010001110?1000011000010000202110101101021110?1001??0????

*Tyrannotitan*

?????????????????????????????????1?????????????????1111????????????????????????????????????????????????????????????????20?010?0??????????1??0?20?0????????01101?1???????1???001???0?00??00?1?001?21???????000??0??????????11????01??????????????????????????????????????????????????0????0?201?????20?????0?220?1?01111?0?01???????2???????????????????????010?

*Xuanhanosaurus*

???????????????????????????????????????????????????????????????????????????????????????????????????????????????????????????????????????????????????????????1100?????????0?????1?????0??000?0??00??1???????1???????????????0?1???1?200?1111?01000010001102?001101?100???????????????????????????????????????????????????????????????????????????????????????????

*'Yangchuano_hepingensis'*

00001000000000??????010002111?1?000110110000211000011100010001001?110000112001000???11?12000?00?????01??????1?1??????0000001?00?001???1?000?00?000?1000010011001101100010010001??10??00110001?00121?1100011000100?0101?11001011100?0????????????????????????????????000010100101?1110112{12}0021110100210110?10110?1101111?00?????????????????????????????????????

*'Yangchuano_magnus'*

0000?000000000??????000002111?1?0001101100002?1000011100010?010?1?1??0?0?120???0?0?????????????????????????0111???1??00000?1?00???1???1?0???00?0?0??00001001100{12}?0110001??10001{12}11010001000?1?00011010000?0000100?010???????????????????????????????????????????????000000100101?111011010120?100?020011?11111??0101?110????100??201??0101101021??0????????????

**References**

Carrano MT, Benson RBJ, Sampson SD. 2012. The phylogeny of Tetanurae (Dinosauria: Theropoda). *Journal of Systematic Palaeontology* 10: 211–300. doi: 10.1080/14772019.2011.630927.

Currie PJ, Zhao X. 1993. A new carnosaur (Dinosauria, Theropoda) from the Jurassic of Xinjiang, People’s Republic of China. *Canadian Journal of Earth Sciences* 30: 2037–2081. doi: 10.1139/e93-179.

O’Connor PM. 2007. The postcranial axial skeleton of *Majungasaurus crenatissimus* (Theropoda: Abelisauridae) from the Late Cretaceous of Madagascar. *Journal of Vertebrate Paleontology* 27: 127–163. doi: 10.1671/0272-4634(2007)27.

Stromer E. 1931. Ergebnisse der Forschungsreisen Prof. E. Stromers in den Wüsten Ägyptens. II. Wirbeltierreste der Baharije-Stufe (unterstes Cenoman). 10. Ein Skelett-Rest von *Carcharodontosaurus* nov. gen. *Abhandlungen der Bayerischen Akademie der Wissenschaften, Mathematisch-naturwissenschaftliche Abteilung, Neue Folge* 9: 1–23.

Xing LD, Bell PR, Rothschild BM, Ran H, Zhang JP, Dong ZM, Zhang W, Currie PJ. 2013. Tooyh loss and alveolar remodeling in Sinosaurus triassicus (Dinosauria: Theropoda) from the Lower Jurassic strata of the Lufeng Basin, China. *Chinese Science Bulletin* 58 (16): 1931-1935. doi: 10.1007/s11434-013-5765-7.
